# Genome-Wide Identification of WRKY Transcription Factors in the Asteranae

**DOI:** 10.3390/plants8100393

**Published:** 2019-10-01

**Authors:** Hongyu Guo, Yantong Zhang, Zhuo Wang, Limei Lin, Minghui Cui, Yuehong Long, Zhaobin Xing

**Affiliations:** 1 College of Life Sciences, North China University of Science and Technology, Tangshan 063210, China; 18331563261@163.com (H.G.); zytheuu@126.com (Y.Z.); wangzhuoheuu@126.com (Z.W.); llm1205258836@163.com (L.L.); cmh19970722@126.com (M.C.); 2 School of Pharmacy, North China University of Science and Technology, Tangshan 063210, China

**Keywords:** the Asteranae, the Apiales, the Asterales, WRKY transcription factors, synteny

## Abstract

The WRKY transcription factors family, which participates in many physiological processes in plants, constitutes one of the largest transcription factor families. The Asterales and the Apiales are two orders of flowering plants in the superorder Asteranae. Among the members of the Asterales, globe artichoke (*Cynara cardunculus* var. *scolymus* L.), sunflower (*Helianthus annuus* L.), and lettuce (*Lactuca sativa* L.) are important economic crops worldwide. Within the Apiales, ginseng (*Panax ginseng* C. A. Meyer) and *Panax notoginseng* (Burk.) F.H. Chen are important medicinal plants, while carrot (*Daucus carota* subsp. *carota* L.) has significant economic value. Research involving genome-wide identification of WRKY transcription factors in the Asterales and the Apiales has been limited. In this study, 490 *WRKY* genes, 244 from three species of the Apiales and 246 from three species of the Asterales, were identified and categorized into three groups. Within each group, WRKY motif characteristics and gene structures were similar. *WRKY* gene promoter sequences contained light responsive elements, core regulatory elements, and 12 abiotic stress *cis*-acting elements. *WRKY* genes were evenly distributed on each chromosome. Evidence of segmental and tandem duplication events was found in all six species in the Asterales and the Apiales, with segmental duplication inferred to play a major role in *WRKY* gene evolution. Among the six species, we uncovered 54 syntenic gene pairs between globe artichoke and lettuce. The six species are thus relatively closely related, consistent with their traditional taxonomic placement in the Asterales. This study, based on traditional species classifications, was the first to identify WRKY transcription factors in six species from the Asteranae. Our results lay a foundation for further understanding of the role of WRKY transcription factors in species evolution and functional differentiation.

## 1. Introduction

WRKY transcription factors, which constitute one of the largest transcription factor families in plants, are involved in growth, development, and biotic and abiotic stress processes. WRKY transcription factors can participate in various plant hormone signaling pathways, such as the gibberellin (GA) and abscisic acid (ABA) pathways, and regulate other physiological processes, including fruit ripening and leaf senescence [1,2]. For example, *HaWRKY10* is regulated by ABA and GA, thereby reducing carbohydrate metabolism and improving lipid metabolism in sunflower seeds [3]. WRKY transcription factors encoding genes in lettuce are mainly expressed in leaves and can regulate bolting [4]. The drought tolerance of *Arabidopsis thaliana* was enhanced after *ZmWRKY106* gene overexpression with drought response was identified in monocotyledon maize [5]. *AtWRKY53* in *A. thaliana* belongs to group III. Under drought climate conditions, it can promote the metabolism of starch in the guard cells and also reduce the H_2_O_2_ content, which ultimately promotes stomatal movement [6]. Furthermore, WRKY can regulate plant secondary metabolic processes, such as artemisinin secondary metabolism. The *AaGSW1* gene, from *Artemisia annua* L. (the Asterales), is a positive regulator in artemisinin biosynthetic pathways and its overexpression can significantly increase artemisinin and dihydroartemisinic acid contents [7].

WRKY proteins have one or two WRKY domains containing the conserved heptapeptide WRKYGQK and a zinc-finger motif. WRKY proteins can be divided into groups I, II, and III according to the number of WRKY domains and the type of zinc-finger motif [8]. Group I, which contains two WRKY domains and a C_2_H_2_ zinc-finger motif, can be further divided into two subgroups: Ia with a C_2_H_2_ zinc-finger motif, and Ib with a C_2_HC zinc-finger motif [9]. Group II contains a WRKY domain and a C_2_H_2_-type zinc-finger motif and is further divided into five subgroups. Group III contains a WRKY domain and a C_2_HC-type zinc finger motif. The WRKY domain binds to a *cis*-acting element, the W box. The core sequence (TGAC/T) of the W box is necessary for binding WRKY, which reflects the conservation of the WRKY domain [8].

Little research has been conducted on genome-wide WRKY transcription factors of sequenced species from orders the Asterales and the Apiales. Ginseng (*Panax ginseng* C. A. Meyer; tetraploid), *Panax notoginseng* ([Burk.] F.H. Chen; diploid), and carrot (*Daucus carota* subsp. *carota* L.; diploid), all members of the Apiales, have important medicinal and food value and a long history of cultivation in China [10,11,12]. Within the Asterales, globe artichoke (Cynara cardunculus var. scolymus L.; diploid), sunflower (*Helianthus annuus* L.; diploid), and lettuce (*Lactuca sativa* L.; diploid) are major economic crops widely planted worldwide. These species are also rich in various phenolic compounds, vitamins, and other substances [13,14,15]. With the development of high-throughput sequencing technology, many species have been subjected to genome sequencing, including rice (*Oryza sativa* L. ssp. *indica*), bread wheat (*Triticum aestivum* L.), and grape (*Vitis vinifera* L.) [16,17,18]. Genome-wide identification and analysis of *WRKY* genes has been carried out in many plant species. *WRKY* gene is only found in land plants [19]. Among model species, 128 *WRKY* genes have identified in rice (*O. sativa* L. subsp. Japonica) and 90 identified in *A. thaliana* [20,21]. Among monocotyledons plants, 136 have been identified in maize, 95 identified in African oil palm (*Elaeis guineensis*), and 121 identified in moso bamboo (*Phyllostachys edulis*) [22,23,24]. Among dicotyledonous plants, 59 have been identified in grape, 145 identified in Chinese cabbage (*Brassica rapa* ssp. *pekinensis*), 105 identified in *Populus*, 75 identified in peanut (*Arachis ipaënsis*), and 67 and 95 identified in carrot, respectively (*Daucus carota* subsp. *sativus* L.; *Daucus carota* L. cv. Kuroda) [25,26,27,28,29,30]. Within superorder the Asteranae, genomes of six species of the Asterales and the Apiales have been sequenced. These data can be applied for in-depth research in evolutionary biology, with the goal of elucidating the origin and evolution of species and enhancing their economic value.

In this study, we identified genome-wide WRKY transcription factors from sequenced genomes of six species of the superorder Asteranae and analyzed phylogenetic relationships, gene structures, *cis*-acting elements, and *WRKY* gene duplication events. Our results establish a foundation for further studies of WRKY transcription factors in the other Asteranae species, including their evolutionary relationships and systematic taxonomy.

## 2. Results

### 2.1. Phylogenetic Analysis and Classification of WRKY Genes

**Figure 1:**
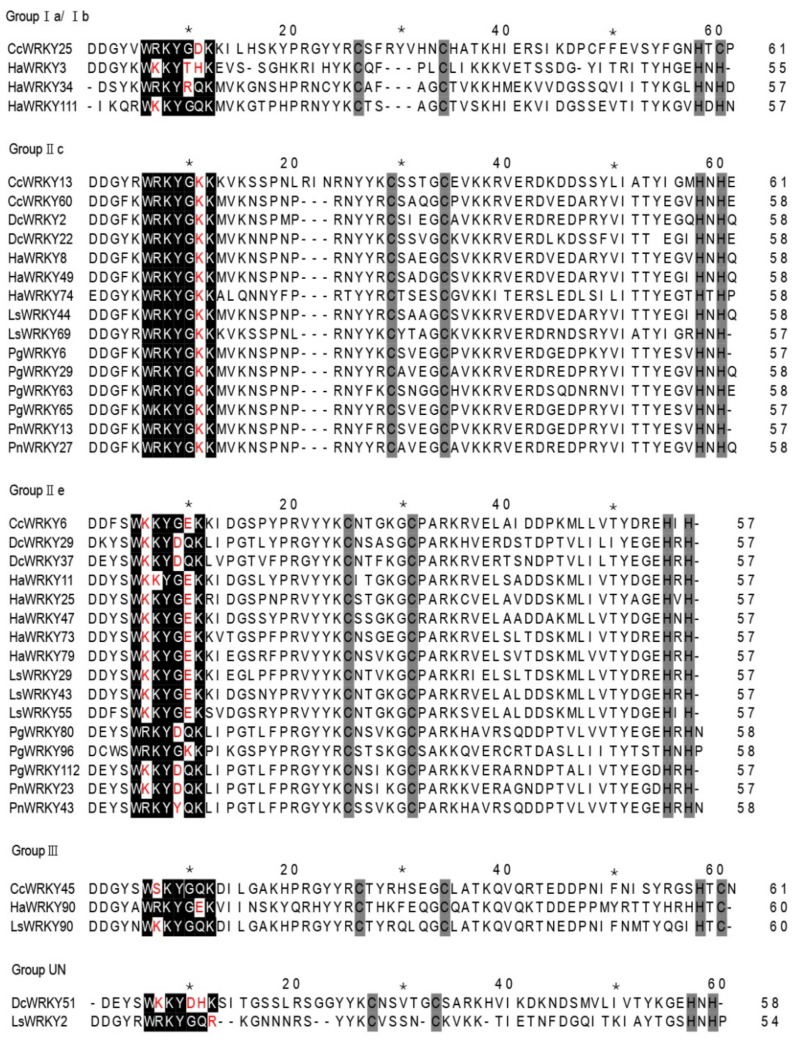
Variants of the conserved heptapeptide sequence of the WRKY domain uncovered in ginseng, *P. notoginseng*, carrot, globe artichoke, sunflower, and lettuce. Amino acids differing from the conserved WRKYGQK sequence are shown in red.

We used the HMMER program with the HMM profile of the WRKY domain (PF03106) as a query to search for *WRKY* genes in *A. thaliana* and six species of the Asteranae. We then searched for WRKY conserved domains in Pfam, CDD, and SMART databases. As a result, 490 *WRKY* genes were finally identified (Table S1) in the following species: Ginseng (123 genes), *P. notoginseng* (56), carrot (65), sunflower (112), globe artichoke (60), and lettuce (74). Moreover, we identified 72 *WRKY* genes in the reference species *A. thaliana*, using the same pipeline. According to their chromosomal distributions, these genes were designated as *PgWRKY1*–*PgWRKY123*, *PnWRKY1*–*PnWRKY56*, *HaWRKY1*–*HaWRKY113*, *CcWRKY1*–*CcWRKY60*, *LsWRKY1–LsWRKY72* and *AtWRKY1–AtWRKY72*. Multiple sequence alignment of WRKY domains from the six Asteranae species revealed the structure of the WRKY domain conserved heptapeptide to be WRKYGQK (Figure S1). A number of variants were also uncovered, including WRKYGDK, WRKYGKK, WSKYGQK, WKKYGEK, WKKYDQK, WKKYDHK, WKKYTHK, WRKYGEK, WRKYRQK, WKKYGKK, WRKYDQK and WRKYYQK (Figure 1), which were distributed in groups Ia, Ib, IIc, IIe, III and unclassified. Except in group IIc, where all variants had the sequence WRKYGKK, no uniform trend was observed in the distribution of variants among groups.

**Figure 2:**
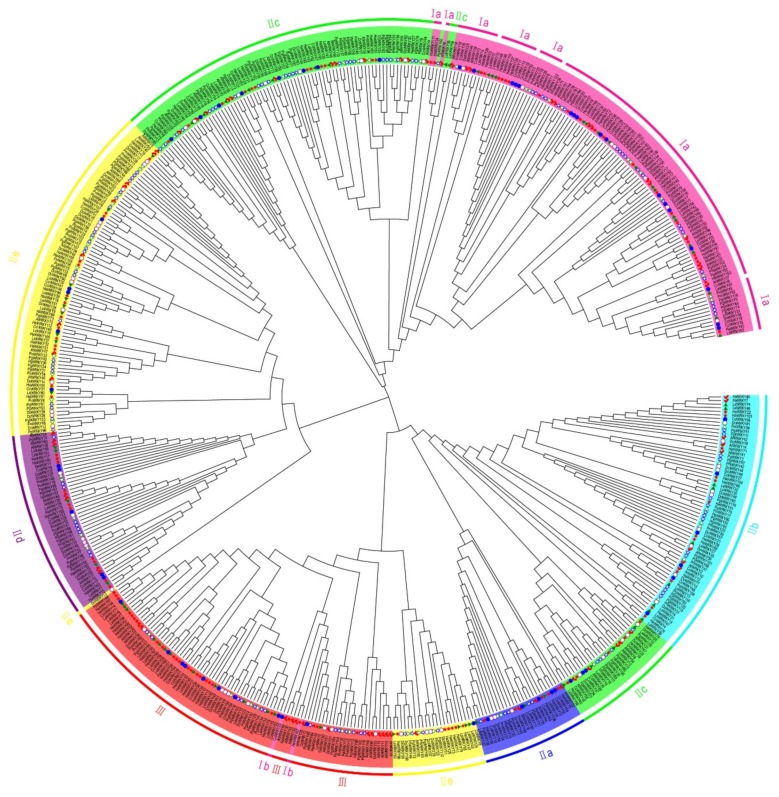
Phylogenetic tree of WRKY domains from ginseng, *P. notoginseng*, carrot, globe artichoke, sunflower, lettuce, and *A. thaliana*. The phylogenetic tree was constructed by the neighbor-joining (NJ) method with 10,000 bootstrap replicates. The different colors of the outer circle are used to denote WRKY groups and subgroups. WRKY genes at terminal nodes in the tree are labeled according to their origin as follows: Solid blue circle, globe artichoke; solid red star, sunflower; solid green triangle, lettuce; open purple circle, carrot; open blue star, ginseng; open green triangle, *P. notoginseng*; solid red check, *A. thaliana*.

The phylogenetic tree was constructed with about 60 amino acids WRKY domain. On the basis of a previous study of *A. thaliana WRKY* genes, their phylogenetic relationships and WRKY conserved domain characteristics, the *WRKY* genes of ginseng, *P. notoginseng*, carrot, sunflower, globe artichoke, lettuce, and *A. thaliana* could be divided into three groups (Figure 2) [8,21]. Group I contained 98 *WRKY* genes classified into subgroups Ia and Ib. Subgroup Ib comprised *CcWRKY25* and *HaWRKY67* genes with a C_2_HC zinc-finger motif, whereas subgroup Ib clustered in the phylogenetic tree with group III members because they shared the same type of zinc-finger motif. Group II could be divided into five subgroups and included 313 (63.8%) *WRKY* genes. Groups IIc and I were relatively closely related, and *WRKY* genes in groups IId and IIe clustered together and thus had a relatively close evolutionary relationship as well. Group III contained 74 *WRKY* genes with a C_2_HC zinc-finger motif; on the basis of their position in the phylogenetic tree, they have appeared relatively recently during WRKY evolution according to the research of the Brand et al. [19]. Because their domain characteristics diverged from those of the others, the remaining five *WRKY* genes could not be classified into the three main WRKY groups (Figure S1). Among the six species, the largest proportion of *WRKY* genes was in group II (ginseng, 69.9%; *P. notoginseng*, 66.1%; carrot, 76.9%; globe artichoke, 55%; sunflower, 57.1%; lettuce, 58.1%). In the phylogenetic tree, *DcWRKY60* was *AtWRKY53* homology gene in Group III, and *AtWRKY53* (AT4G23810) has been shown to have drought tolerance, presumably *DcWRKY60* has the same function [6]. WRKY homologs located under the same group may have similar functions.

### 2.2. Conserved Motif and Gene Structure Analysis of WRKY Genes

**Figure 3:**
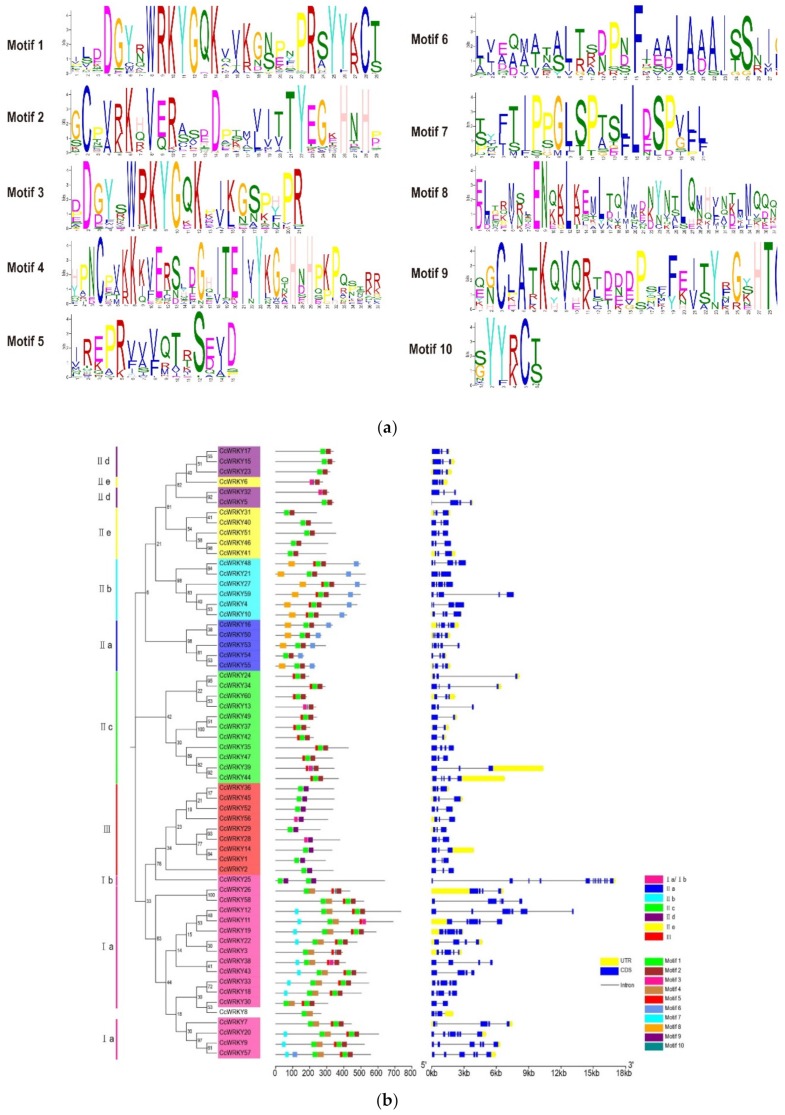
(**a**) Ten types of conserved motifs in globe artichoke. (**b**) Phylogenetic relationships, conserved motifs, and intron–exon structures of *CcWRKY* genes in globe artichoke. On the left is a phylogenetic tree of CcWRKY domains constructed by neighbor-joining with 3000 bootstrap replicates. CcWRKY conserved motifs are shown to the right of the tree. The 10 different conserved motifs are represented by different colors. *CcWRKY* gene intron–exon structures are shown on the far right.

We analyzed the conserved motifs of WRKY proteins in the six species using MEME online software. We found 10 conserved motifs (Table S2) involving the WRKY domain conserved heptapeptide (WRKYGQK), zinc-finger motifs, and the remaining WRKY conserved proteins. The motifs identified in globe artichoke included motifs 1 and 3 containing the WRKY domain conserved heptapeptide, and motifs 2 and 9 with a zinc-finger motif (Figure 3a). Motifs 1 and 3 were present in globe artichoke in group I members containing two WRKY domains. In contrast to other members of group I, CcWRKY25 (Ib) possessed motif 9. Motif 7 was only found in group I, and members of group III only contained motifs 1, 3 and 9. Motif 10, the rarest motif, was distributed in all three groups (Figure 3b). Among the motifs found in carrot were motifs 1 and 3 containing the WRKY domain conserved heptapeptide, and motifs 2 and 10 containing a zinc-finger motif (Figure S2a). Motifs 1 and 3 in carrot were found in group I. Motif 9 was only found in group IIb; this motif was the least abundant of the seven different types of motifs present in the group (Figure S2b). Motifs uncovered in sunflower included 1, 2 and 5 containing WRKY domain conserved heptapeptide, and 3 and 4 containing a zinc-finger motif (Figure S3a). HaWRKY34 (in group I) possessed six WRKY domain conserved heptapeptides. HaWRKY67 (in group Ib) contained motif 4; its zinc-finger motif was different from that of other group members, similar to CcWRKY25 mentioned above. The greatest similarity in motifs in sunflower was between groups IIa and IIb, which had a relatively close evolutionary relationship. Motif 10, the rarest motif, was distributed in HaWRKY domains (Figure S3b). In lettuce, identified motifs were WRKY domain conserved heptapeptide containing motifs 1 and 3, and motifs 2 and 4 (Figure S4a). Group I WRKY in lettuce possessed motifs 5 and 6, which also contained WRKY domain conserved heptapeptide and a zinc-finger motif. Motif 6 was only present in lettuce in group I. Group IIb, with seven motif types, possessed the greatest diversity of conserved motifs, including seven kinds. LsWRKY45 did not contain motifs 1 and 3, possibly because of the absence of a WRKY domain (Figure S4b). In ginseng, motifs 1, 2 and 5 contained WRKY domain conserved heptapeptide, and motifs 3, 4, 6 and 9 contained a zinc-finger motif (Figure S5a). Motif 7 was also mainly found in ginseng in group I, along with motifs 1, 2, 4, 5 and 6 (Figure S5b). Motifs found in *P. notoginseng* included 1 and 3 containing WRKY domain, and 2 and 4 containing a zinc-finger motif (Figure S6a). PnWRKY40 and PnWRKY51 in group I possessed a large number of copies of motif 10. Group IIb contained seven types of motifs. Motif 8 was mainly distributed in groups IId and IIe. Conserved motifs in group IIb were the most diverse of all WRKY proteins of the six studied species other than sunflower. Among the 10 motifs, motifs 9 and 10 were the least abundant of any found in the six species, and the remaining WRKY conserved protein sequences exhibited no uniformity.

*WRKY* intron–exon structures were characterized by analysis of genomic data using TBtools. The number of introns in globe artichoke *WRKY* ranged from 1 to 12, with an average of 2.9 per gene (Figure 3b). *CcWRKY25* contained 12 introns, the largest number detected. The intronic distribution of *CcWRKY* genes in group III was conserved, with each gene contained two introns. The number of introns in carrot ranged from two to nine, with an average of 2.6 introns per *DcWRKY* gene (Figure S2b). The number of introns per *HaWRKY* gene in sunflower ranged from two to nine, with an average of 2.6 (Figure S3b). *HaWRKY* genes in group IIa contained four introns, while group IIe members possessed one. Most *HaWRKY* genes in group III contained two introns. Group Ib *WRKY* genes were present in both globe artichoke and sunflower, and their gene structures were similar, with more introns than those of other group members. The number of introns in lettuce ranged from one to six, with an average of 2.6 introns per *LsWRKY* gene (Figure S4b). Group IId gene structures were similar, with all containing two introns. With one exception, all members of group III contained two introns; *LsWRKY45* contained only one. The number of introns in ginseng ranged from 1 to 36, with an average of 3.4 (Figure S5b). *PgWRKY* genes in group I contained four introns on average. *PgWRKY85* (IIb), *PgWRKY90* (IId), and *PgWRKY110* (IIe) genes, respectively, contained 21, 36, and 28 introns, and the number of introns and the sequence lengths were much larger than those of other *PgWRKY* genes. *PnWRKY* genes in *P. notoginseng* possessed one to nine introns, and the average was 3.3 introns per gene (Figure S6b). *PnWRKY49* (IIe) contained nine introns, the most in its group. The number of introns was conserved in *PnWRKY* genes in group III, which contained two introns. *WRKY* genes in group I contained at least three introns on average, the highest of any group in the six species; in globe artichoke, *WRKY* genes in group I contained an average of six introns (Figure 3b). The structure of *WRKY* genes in group Ib was different from other group members, and the number of introns was higher than that of other *WRKY* genes (Figure 3b and Figure S3b). The gene structure of group III members was the most conserved among the six species, while that of group Ib was the most distinct. Except for five *WRKY* genes in ginseng, only two introns were present in group III, and it was thus the most conserved group of *WRKY* genes (Figure S5b).

Conserved motif and intron–exon distribution patterns of *WRKY* genes in the six species were generally group specific, while *WRKY* gene structures were similar within each group, thereby verifying the phylogenetic relationships of the six species. The distribution of introns in groups I and III in the six species also exhibited similarities and thus also reflected the evolutionary relationships of the six species.

### 2.3. Analysis of WRKY Gene Promoters

To study the expression and regulation of WRKY transcription factors, we used PLANTCARE software to analyze *cis*-acting elements in *WRKY* promoters (data not shown). Our analysis revealed that carrot, globe artichoke, sunflower, and lettuce contained a large number of promoter core regulatory elements (CAAT-box), light responsive elements (GT1-motif, Sp1 and ACE), and W box elements. We also uncovered a variety of abiotic stress responsive elements, such as drought-inducibility elements (MBS), flavonoid biosynthetic gene regulators (MBS I), low-temperature responsive elements (LTR), defense and stress responsive elements (TC-rich repeates), abscisic acid responsive elements (ABRE), methyl jasmonate (MeJA) responsive elements (CGTCA-motif and TGACG-motif), auxin-responsive elements (TGA-element and AuxRR-core), gibberellin-responsive elements (GARE-motif, P-box, and TATC-box), circadian control elements (circadian), salicylic acid responsive elements (TCA-element), and wound-responsive elements (WUN-motif).

**Figure 4:**
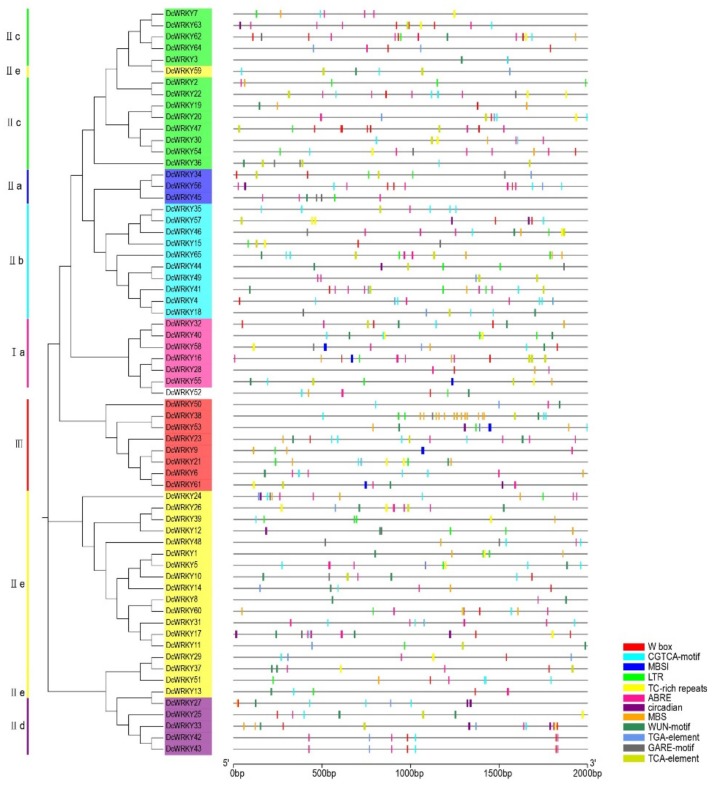
*Cis*-acting elements in carrot *DcWRKY* promoters. The distribution of 12 *cis*-acting elements in the 2000 bp upstream promoter are shown. The different types of *cis*-acting elements are represented by different colors, as shown on the right. W box, WRKY binding site; CGTCA-motif, *cis*-acting regulatory element involved in the MeJA-responsiveness; MBS I, MYB binding site involved in flavonoid biosynthetic genes regulation; LTR, *cis*-acting element involved in low-temperature responsiveness; TC-rich repeats, *cis*-acting element involved in defense and stress responsiveness; ABRE, *cis*-acting element involved in the abscisic acid responsiveness; circadian, *cis*-acting regulatory element involved in circadian control; MBS, MYB binding site involved in drought-inducibility; WUN-motif, wound-responsive element; TGA-element, auxin-responsive element; GARE-motif, gibberellin-responsive element; TCA-element, *cis*-acting element involved in salicylic acid responsiveness.

Some abiotic stress response elements and W box elements were visualized using TBtools. In carrot, W box elements were found in 33 promoters of *DcWRKY* genes (Figure 4). The most common elements were ABRE, contained in 49 *DcWRKY* promoters, followed by CGTCA motif elements; in contrast, MBS I were the least abundant elements. *DcWRKY38* genes (in group III) contained 13 MBS elements, the highest of any group in *DcWRKY* genes. W box elements were found in 41 promoters of *CcWRKY* genes from globe artichoke (Figure S7). LTR elements were present in 23 promoters of *CcWRKY*. MeJA responsive elements (CGTCA-motif), which were found in 45 *CcWRKY* promoters, accounted for the largest proportion of *cis*-acting elements, followed by ABRE elements. The rarest *cis*-acting element in *CcWRKY* promoters was MBS I. In sunflower, 76 promoters of *HaWRKY* genes contained W box elements (Figure S8). CGTCA-motif elements, which were present in 96 *HaWRKY* genes, accounted for the largest proportion of abiotic stress elements, followed by ABRE elements. The least common *cis*-acting elements in *HaWRKY* promoters were the circadian elements. In lettuce, 40 promoters of *LsWRKY* genes contained W box elements (Figure S9). A total of 55 *LsWRKY* genes possessed ABRE elements; these were the most abundant abiotic stress elements, followed by CGTCA-motif elements. In contrast, only six *LsWRKY* promoters contained MBS I elements. The *LsWRKY26* gene did not contain these abiotic stress elements or any other elements. The most common *cis*-acting elements among globe artichoke, carrot, sunflower, and lettuce were MeJA responsive elements and ABRE elements, whereas MBS I elements were the rarest in three of these species.

### 2.4. Chromosomal Distribution and Duplication of WRKY Genes

**Figure 5:**
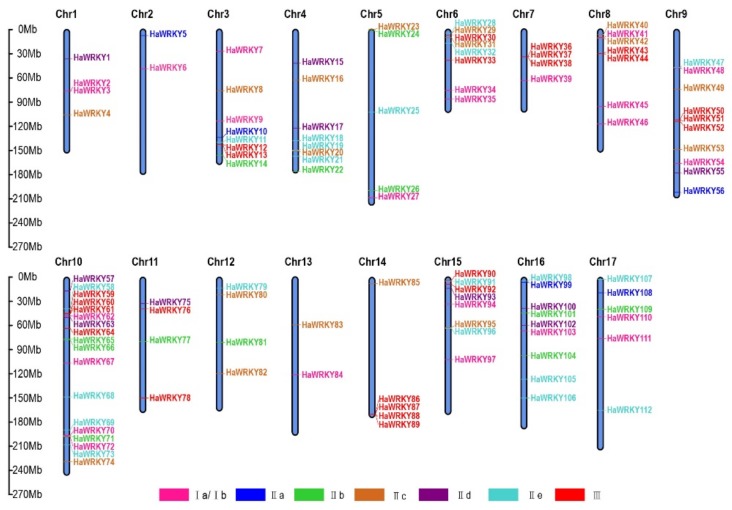
Distribution of *HaWRKY* genes on sunflower chromosomes. Vertical bars represent chromosomes. The different groups of *HaWRKY* genes are color-coded. Base-pair positions are indicated on the left.

We further studied the evolution of the *WRKY* gene family by analyzing the chromosomal distribution of *WRKY* genes. Since the ginseng and *P. notoginseng* genome assembly information is incomplete, only the other four species were analyzed at the chromosome level. In sunflower, 112 *HaWRKY* genes were distributed on 17 chromosomes (Figure 5). Chromosome 10 contained 18 *HaWRKY* genes, whereas chromosomes 2 and 13 contained only 2. The proportion of *HaWRKY* genes in group I and III was 21.4%, while those of group IIa were the least (4.4%). Five gene clusters (comprising *HaWRKY2*, *HaWRKY3*; *HaWRKY12*, *HaWRKY13*; *HaWRKY36*, *HaWRKY37*; *HaWRKY59*, *HaWRKY60*; *HaWRKY86*, *HaWRKY87*, and *HaWRKY88*) were found on chromosomes 1, 3, 7 10, and 14. A total of 60 *CcWRKY* genes were distributed on the 17 chromosomes of globe artichoke (Figure S10). Chromosome 2 had the most *CcWRKY* genes, while chromosomes 4, 5, and 10 contained only one. On the 17 chromosomes, the proportion of *CcWRKY* genes in group III was 11.4%, while groups IIa and IId were the least common (8.3%). A cluster of genes (CcWRKY53, CcWRKY54, and CcWRKY55), all belonging to group IIa, was present on chromosome 16. A total of 74 *LsWRKY* genes were distributed on the nine chromosomes of lettuce (Figure S11). Chromosome 9 harbored the most *LsWRKY* genes (nine), while chromosomes 1 and 6 had the fewest. *LsWRKY* genes in group IIa were the least (4.0%). Two clusters of genes belonging to group III (*LsWRKY5*, *LsWRKY6*, *LsWRKY7*; *LsWRKY65*, *LsWRKY66*, *LsWRKY67*, and *LsWRKY68*) were uncovered on chromosomes 2 and 9. In carrot, 65 *DcWRKY* genes were distributed across nine chromosomes (Figure S12). Chromosome 2 had the highest number of *DcWRKY* genes (16 genes), while chromosome 9 contained only three. *DcWRKY* genes in group IIe were the most widely distributed on chromosomes (29.2%), whereas group IIa was the least (4.6%). A cluster of group IIc genes (*DcWRKY42* and *DcWRKY43*) was present on chromosome 5. Among the four species, *WRKY* genes in group IIa were the least common, and these four species contained at least one gene cluster.

**Figure 6:**
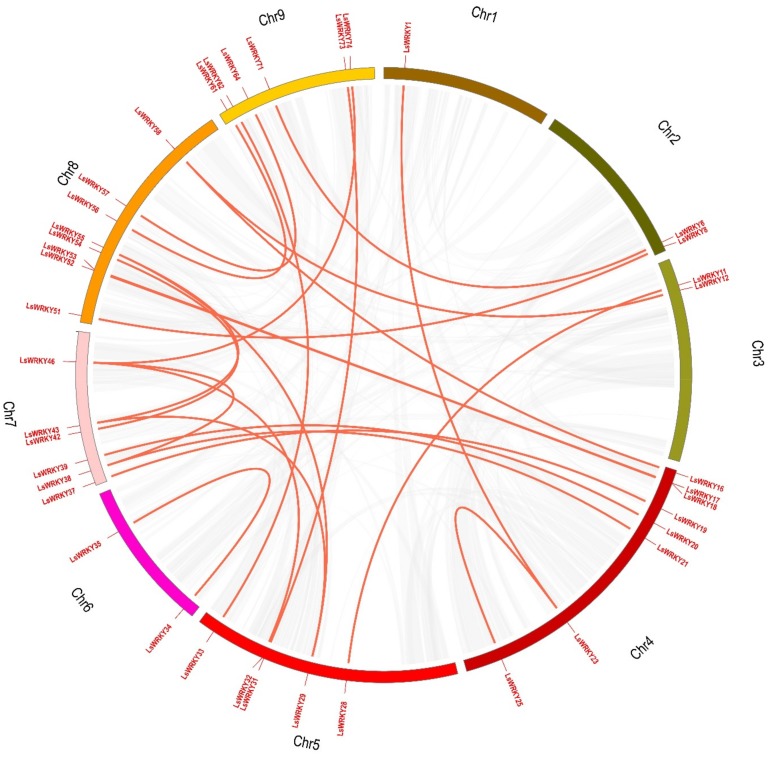
Synteny analysis of lettuce *LsWRKY* genes. Red lines indicate segmentally duplicated gene pairs, and gray lines connect syntenic blocks in the genome. Chromosomes 1–9 are represented by different colors.

In this study, we calculated syntenic relationships in the four species using MCscanX. On the nine chromosomes of lettuce, we uncovered 24 *LsWRKY* gene pairs derived from segmental duplication of 40 *LsWRKY* genes (on all chromosomes) from groups I (25. 0%), IIe (22.5%), and IIb (20%) (Figure 6). Chromosomes 4 and 8 had the highest number of *LsWRKY* genes (eight in both cases). Five tandem duplicated gene pairs were found in lettuce (*LsWRKY5*–*LsWRKY6*, *LsWRKY6*–*LsWRKY7*, *LsWRKY65*–*LsWRKY66*, *LsWRKY66*–*LsWRKY67*, and *LsWRKY67*–*LsWRKY68*). We identified 19 *CcWRKY* gene pairs derived from segmental duplication events involving 24 *CcWRKY* genes belonging to group I (50%) on the 14 chromosomes of globe artichoke (Figure S13). We did not detect any segmental duplicated *CcWRKY* genes on chromosomes 1, 10, or 14, but two tandem duplicated gene pairs (*CcWRKY53*–*CcWRKY54* and *CcWRKY54*–*CcWRKY55*) were identified. On the nine chromosomes of carrot, we found 23 *DcWRKY* segmental duplicated gene pairs involving 30 *DcWRKY* genes belonging to groups IIb (30%) and IIe (33.3%) (Figure S14). Chromosome 5 harbored the largest number of *DcWRKY* genes (eight). Only one tandem duplicated gene pair (*DcWRKY42*–*DcWRKY43*) was found in carrot. In sunflower, 11 *HaWRKY* gene pairs were identified as arising from segmental duplication events on 10 chromosomes; these pairs were composed of 21 *HaWRKY* genes belonging to groups IIe (38.1%) and IId (28.6%) (Figure S15). Chromosome 10 had the most *HaWRKY* genes (four). We did not detect any segmentally duplicated *HaWRKY* genes on chromosomes 1, 2, 3, 7, 11, 13, or 14. Six tandem duplicated gene pairs were found on 17 chromosomes (*HaWRKY2*–*HaWRKY3*, *HaWRKY12*–*HaWRKY13*, *HaWRKY36*–*HaWRKY37*, *HaWRKY59*–*HaWRKY60*, *HaWRKY86*–*HaWRKY87*, and *HaWRKY87*–*HaWRKY88*).

### 2.5. Analysis of Synteny Between Asterales and Apiales Genomes

**Figure 7:**
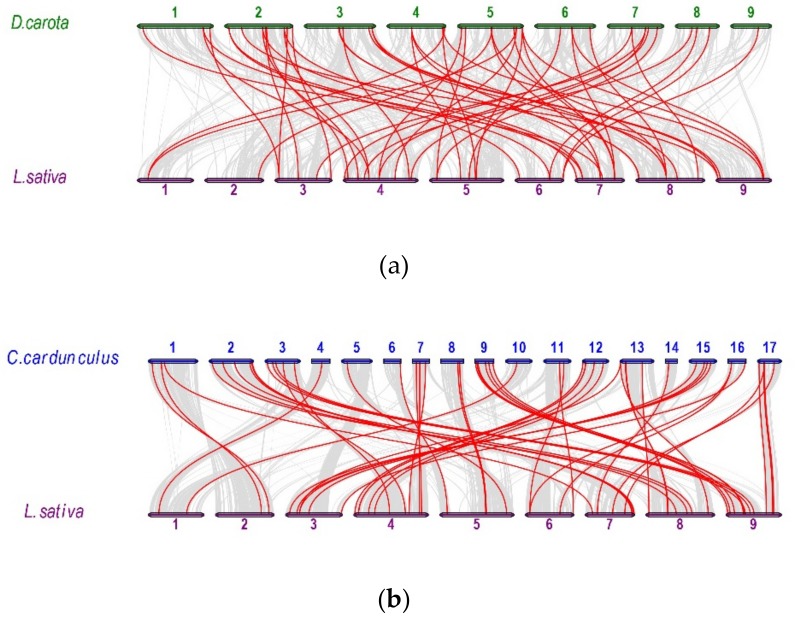
Synteny between genomes of lettuce (*Lactuca sativa*) and (**a**) carrot (*Daucus carota*) and (**b**) globe artichoke (*Cynara cardunculus*). Gray lines represent syntenic blocks between genomes, and red lines indicate syntenic *WRKY* gene pairs.

To further deduce the evolutionary relationships of *WRKY* gene family members in carrot, globe artichoke, sunflower, and lettuce, we analyzed syntenic relationships among the four species. Six groups of syntenic relationships were identified (Table S3). The number of syntenic *WRKY* gene pairs between species was as follows: 10 between carrot and globe artichoke (on six and seven chromosomes, respectively; Figure S16), 19 between carrot and sunflower (seven and eight chromosomes, respectively; Figure S17), 52 between carrot and lettuce (nine chromosomes each; Figure 7a), 31 between sunflower and lettuce (13 and 9 chromosomes, respectively; Figure S18), 23 between sunflower and globe artichoke (11 and 14 chromosomes, respectively; Figure S19), and 54 between globe artichoke and lettuce (all chromosomes; Figure 7b). Genes *DcWRKY14*, *HaWRKY28*, *HaWRKY41*, *HaWRKY57*, *HaWRKY58*, *HaWRKY99*, *CcWRKY31*, *CcWRKY35*, *CcWRKY48, LsWRKY1*, *LsWRKY9*, *LsWRKY13*, *LsWRKY19*, *LsWRKY20*, *LsWRKY22*, *LsWRKY31*, *LsWRKY34*, *LsWRKY38*, *LsWRKY55*, *LsWRKY56*, *LsWRKY58*, *LsWRKY60* and *LsWRKY61* were involved in the syntenic relationships among the six groups.

In addition, 189 pairs of orthologous genes between the four species were identified. To study evolutionary pressures acting on *WRKY* genes, we aligned coding sequences of orthologous and tandem duplicated gene pairs (paralogous gene pairs) of the four species (Table S4) and calculated the non-synonymous to synonymous substitution-rate ratio (Ka/Ks) of *WRKY* genes. A Ka/Ks > 1 was obtained for the *CcWRKY53*–*CcWRKY55* gene pair in globe artichoke, which indicates that positive selection pressure may have operated on *CcWRKY* genes. No Ka/Ks ratios were calculated for tandem duplicated genes in lettuce or carrot because these genes had low similarities. Six tandem duplicated *WRKY* gene pairs were identified in sunflower with a Ka/Ks < 1, which suggests the action of purifying selection pressure on *WRKY* genes in this species (Table 1). A total of 76.1% of orthologous genes had Ka/Ks values <1, thus indicating that *WRKY* genes in globe artichoke, sunflower, carrot, and lettuce may have been subjected to purifying selection pressure during species evolution.

## 3. Discussion

The evolution, function, abiotic stress response, and other aspects of WRKY, one of the largest plant transcription factor families, have been investigated in many species of plants. In the present study, 490 *WRKY* gene family members were identified from six species of the superorder Asteranae. We identified 65 *WRKY* genes in carrot, which compares with 67 in a previous study of the same variety [29]. This difference may be due to the fact that the E values were set differently when the domains were screened. Consequently, we identified a different number of *WRKY* genes. Furthermore, this study was reconfirmed through the database to remove the incomplete WRKY domain and zinc-finger motifs. In another study, 95 *WRKY* genes were identified in a different variety (*D. carota* L. cv. Kuroda) [30].

Among the 490 *WRKY* genes uncovered in the six species, 485 could be divided into three groups. The remaining five *WRKY* members could not be assigned to any of the three groups because of differences in their domain characteristics. Group Ib includes *CcWRKY25* and *HaWRKY67* genes. Previous studies have reported the presence of group Ib in some monocotyledonous plants; for example, eight *WRKY* genes found in rice, six in *O. officinalis* Wall ex Watt, and one (*PheWRKY61*) in moso bamboo [9,24,31]. The only *WRKY* genes in group Ib uncovered in our study were in globe artichoke and sunflower (all dicotyledonous plants). Group Ib is a new group produced by the duplication of the DNA binding domain in group III, and is currently only found in monocotyledonous plants [19]. The existence of group Ib in dicotyledonous plants can also provide a basis for studying evolution of monocotyledonous and dicotyledonous plants.

The proportion of *WRKY* genes represented by each group differed among the six species. Among the other species, the largest proportion of *WRKY* genes was also in group II, such as rice (46 genes, 56.8%) and grape (40, 69.0%). We identified 12 WRKY domain variants, including WRKYGKK, WKKYDQK, and WKKYGEK, in WRKY genes of the six species. Such variation, which has been reported in many species [9,32,33], may have an effect on the normal physiological metabolic functions of *WRKY* genes. The conserved nature of the WRKY domain heptapeptide of group IIc may be a reflection of the ancestral position of this group in the evolutionary tree. In contrast, group Ib have arisen more recently via DNA binding domain duplication, with subsequent base mutations taking place during duplication [19].

MEME analysis of WRKY protein sequences revealed obvious group specificities. For example, WRKY proteins belonging to group IIc in ginseng are relatively conserved and rarely contain other motifs. We observed similar phenomena in the remaining five species. Group Ib includes *CcWRKY25* and *HaWRKY67* genes. C_2_H_2_ zinc-finger motifs are present in N- and C-terminal regions of the proteins of these two genes, similar to the zinc-finger motifs of group III. Although *CcWRKY25* and *HaWRKY67* genes fell into group III in the phylogenetic tree, they contain two WRKY domains and thus belong to group I. Group Ib has arisen from group III via the duplication of *WRKY* genes [19]; this WRKY transcription factor’s evolutionary patterns has also been confirmed in wild rice [31]. Although *PgWRKY42* and *PgWRKY122* of ginseng cannot be classified into any of the three groups in the phylogenetic tree, they cluster with group I and may have originated through the loss of the WRKY domain during the course of evolution. Similarly, *LsWRKY2*, *DcWRKY52*, and *CcWRKY8* are also closely related to group I, but they contain only one WRKY domain.

Our examination of *WRKY* intron–exon structural characteristics in the six species indicated that the gene structure of group III is the most conserved. Moreover, the number of *WRKY* gene introns in group I is higher than that of the other two groups, possibly because of gene duplication in ancestral group IIc. The number of introns in *CcWRKY25* and *HaWRKY67* is different that of other group Ib members, with 12 and 9 introns, respectively, inserted into exons, a situation that may be due to an increase in the number of introns caused by gene duplication during evolution. The structural characteristics of *PgWRKY85*, *PgWRKY90*, and *PgWRKY110* are different from those of other members in ginseng, and the number of introns is higher. Furthermore, the functions of these three genes in plants may be different from those of other members.

*Cis*-acting elements play an important role in gene transcription and expression. We analyzed *cis*-acting elements of four species in the Asterales and the Apiales and found a large number of hormone response elements (ABRE, CGTCA-motif, TGA-element, GARE-motif, TCA-element), stress response elements (MBS, LTR, TC-rich repeates, and WUN-motif), W box, and secondary metabolic pathway-related (MBS I) elements. *DcWRKY38*, which belongs to group III in carrot with same as *AtWRKY53*, contains 13 MBS elements and may also be involved in plant drought resistance [6]. MeJA elements are one of the most abundant of the 12 types of *cis*-acting elements in globe artichoke, sunflower, carrot, and lettuce. Exogenous MeJA induces *WRKY* genes containing MeJA elements and may enhance the activity of *WRKY* gene promoters in the six species, thereby participating in the regulation of other plant physiological processes. In *Conyza blinii* H. Lév, expression of the *CbWRKY24* gene leads to increased total saponin content and subsequent upregulation of the transcription of key enzyme genes in the mevalonate pathway. In tomato, expression of *CbWRKY24* downregulates the expression of key genes in the lycopene pathway [34]. *CrWRKY1* identified in *Catharanthus roseus* was overexpressed under the induction of plant hormones such as MeJA, resulting in upregulation of key enzyme genes in the terpenoid indole alkaloids (TIAs) pathway [35]. WRKY transcription factors can specifically bind to W box elements in gene promoters and affect gene transcription [36]. The WsWRKY1 transcription factor in *Withania somnifera* can directly regulate the triterpenoid metabolic pathway by binding to the W box elements in the squalene synthase and squalene epoxidase gene promoter regions and enhance synthesis of triterpenoids [37]. The TaWRKY2 and TaWRKY19 proteins of wheat are overexpressed in *A. thaliana* and bind to their downstream gene promoter regions to regulate their gene expression and regulate plant tolerance under stress conditions [38]. *WRKY* genes are induced by flk22, in which the induced WRKY can rapidly bind to its own promoter or other *WRKY* gene to establish network [39]. In addition, WRKY transcription factors can combine with W box elements in their own gene promoters. In chickpea infected with *Fusarium*, for example, WRKY40 has been found to bind to its own promoter to regulate its gene expression [40]. Many genes in the three Asterales species examined in this study contain W box elements. For instance, 41 *CcWRKY* genes in globe artichoke include W box elements, thus suggesting that these *CcWRKY* genes are also regulated by WRKY transcription factors or themselves [36].

Gene duplication plays a key role in species evolution, genome amplification, and gene family evolution [41]. The three main types of gene duplication are whole genome duplication, tandem duplication, and segmental duplication [42]. In this study, we mainly analyzed tandem duplication and segmental duplication events in the four species. We identified 19, 23, 11 and 24 segmental duplicated *WRKY* gene pairs in globe artichoke, carrot, sunflower, and lettuce, respectively. Tandem duplicated genes were relatively less common, a finding consistent with observations in watermelon and pineapple [43,44]. This result also implies that segmental duplication is the main type of gene duplication occurring in the four species. In the four species, the tandem duplicated gene pairs are all located on the same chromosome. Two, one, five, and two tandem clusters (gene clusters) were found in globe artichoke, carrot, sunflower, and lettuce, respectively. Gene clusters have been found in radish, *O. officinalis*, and other species [31,45]. Duplicated genes may have different expressions. These genes may exhibit functional diversity, and the mechanisms underlying their expression regulation may also change. These mechanisms can be identified by sequencing [46]. 

We analyzed the synteny of *WRKY* genomes of four species: Carrot, globe artichoke, sunflower, and lettuce. The largest number of syntenic relationships was found between globe artichoke and lettuce. This result indicates that these species have a relatively close evolutionary relationship, consistent with traditional classifications in the Asterales. The smallest number of syntenic relationships was found between carrot and globe artichoke, which belong to different orders. This outcome may be due to the incomplete assembly of chromosomes in the globe artichoke genome. In the six groups of syntenic relationships, 23 *WRKY* genes were common among the four species and may reveal insights into the evolution of *WRKY* genes among different species. 

Purifying selection pressure has acted on tandem duplicated gene pairs in sunflower, which is in line with findings in peanut and pineapple [28,44]. Only one pair of tandem duplicated genes (*CcWRKY53*–*CcWRKY55* belonging to group IIa) was uncovered in globe artichoke with Ka/Ks > 1, which suggests that positive selection has acted on *CcWRKY53*–*CcWRKY55*. Most orthologous genes among the four species had a Ka/Ks < 1. Compared with paralogous pairs, *WRKY* genes may be purified by whole genome duplication or species differentiation in carrot, sunflower, lettuce, and globe artichoke. In a comparison of *WRKY* orthologous pairs of watermelon, melon, and cucumber, strong purifying selection pressure was inferred to have operated during the evolution of these three species of Cucurbitaceae [47]. A similar conclusion has been drawn for the evolution of pineapple *WRKY* genes [44].

## 4. Materials and Methods

### 4.1. Identification of WRKY Genes

In this study, six species in the superorder Asteranae with sequenced genomes (ginseng, *P. notoginseng*, carrot, sunflower, globe artichoke, and lettuce) and the model plant *A. thaliana* were analyzed [48,49,50,51,52,53,54]. Genome sequences of the selected species were, respectively, downloaded from GigaDB (http://gigadb.org/), http://www.plantkingdomgdb.com/, Ensembl Plants (http://plants.ensembl.org/index.html), NCBI (https://www.ncbi.nlm.nih.gov/), and the Lettuce Genome Resource (http://lgr.genomecenter.ucdavis.edu/). The HMM profile of the WRKY domain (PF03106) was downloaded from the Pfam database (http://pfam.xfam.org/) and used for identification of *WRKY* genes in HMMER with cut-off E-value 0.001 for domain screening [55]. The identities of all *WRKY* genes were confirmed by comparison against the Pfam, CDD (https://www.ncbi.nlm.nih.gov/cdd/), and SMART (http://smart.embl.de/smart/batch.pl) databases to contain the complete WRKY domain (containing conserved heptapeptide WRKYGQK and a zinc-finger motif).

### 4.2. Classification and Phylogenetic Analysis of WRKY Genes

WRKY domains were multiply aligned in ClustalW and displayed using GeneDoc. A neighbor-joining phylogenetic tree was then constructed in MEGA7.0 with default parameters (http://www.megasoftware.net/). Then WRKY genes were divided into three groups on the basis of domain characteristics and phylogenetic relationships.

### 4.3. WRKY Protein Conserved Motif and Gene Structure Analysis

WRKY protein conserved motifs were analyzed in MEME (http://meme-suite.org) with the following parameters: Maximum number of motifs = 10, and motif length = 6–50 residues. *WRKY* gene structure, including intron and exon information, in the six species was visualized using TBtools (https://github.com/CJ-Chen/TBtools) [56].

### 4.4. Chromosomal Localization of WRKY Genes and Cis-Acting Element Analysis

The distribution of *WRKY* genes on carrot, sunflower, globe artichoke, and lettuce chromosomes was analyzed using TBtools. *Cis*-acting elements of *WRKY* genes, analyzed using PLANTCARE (http://bioinformatics.psb.ugent.be/webtools/plantcare/html/), were visualized with TBtools. 

### 4.5. Analysis of WRKY Gene Duplication and Synteny Among Species

To analyze gene duplication events, each WRKY protein sequence from carrot, sunflower, globe artichoke, and lettuce was aligned against itself using BLASTp with an E-value <1e -10 threshold and default parameters. MCScanX was used to analyze *WRKY* gene duplication events and detect syntenic relationships among species. The *WRKY* genes were then mapped to chromosomes to illustrate segmental duplication gene pairs using Circos (http://circos.ca/). Kaks_Calculator 2.0 software was used to calculate the Ka/Ks ratio of orthologous and paralogous *WRKY* gene pairs.

## Figures and Tables

**Table 1:** Ka/Ks values of tandem duplicated *WRKY* genes in sunflower.

Tandem Duplication Gene Pairs	Ka	Ks	Ka/Ks
*HaWRKY51–HaWRKY92*	0.0678063	0.174569	0.388421
*HaWRKY51–HaWRKY52*	0.0678063	0.174569	0.388421
*HaWRKY104–HaWRKY66*	0.112547	0.468332	0.240314
*HaWRKY9–HaWRKY84*	0.088504	0.399459	0.22156
*HaWRKY91–HaWRKY58*	0.103471	0.483353	0.214069
*HaWRKY95–HaWRKY20*	0.0970665	0.371965	0.260956

## Supplementary Materials

The following are available online at https://www.mdpi.com/2223-7747/8/10/393/s1: Table S1: Identification of *WRKY* genes in globe artichoke, sunflower, lettuce, carrot, ginseng and *P. notoginseng*; Figure S1: Alignment of the WRKY domain uncovered in ginseng, ginseng, *P. notoginseng*, carrot, globe artichoke, sunflower, and lettuce; Table S2: WRKY protein sequences of globe artichoke, sunflower, lettuce, carrot, ginseng, and *P. notoginseng*; Figure S2a: Ten types of conserved motifs in carrot; Figure S2b: Phylogenetic relationships, conserved motifs, and intron–exon structures of *DcWRKY* genes in carrot; Figure S3a: Ten types of conserved motifs in sunflower; Figure S3b: Phylogenetic relationships, conserved motifs, and intron–exon structures of *HaWRKY* genes in sunflower; Figure S4a: Ten types of conserved motifs in lettuce; Figure S4b: Phylogenetic relationships, conserved motifs, and intron–exon structures of *LsWRKY* genes in lettuce; Figure S5a: Ten types of conserved motifs in ginseng; Figure S5b: Phylogenetic relationships, conserved motifs, and intron–exon structures of *PgWRKY* genes in ginseng; Figure S6a: Ten types of conserved motifs in *P. notoginseng*; Figure S6b: Phylogenetic relationships, conserved motifs, and intron–exon structures of *PnWRKY* genes in *P. notoginseng*; Figure S7: *Cis*-acting elements in globe artichoke *CcWRKY* promoters; Figure S8: *Cis*-acting elements in sunflower *HaWRKY* promoters; Figure S9: *Cis*-acting elements in lettuce *LsWRKY* promoters; Figure S10: Distribution of *CcWRKY* genes on globe artichoke chromosomes; Figure S11: Distribution of *LsWRKY* genes on lettuce chromosomes; Figure S12: Distribution of *DcWRKY* genes on carrot chromosomes; Figure S13: Synteny analysis of globe artichoke *CcWRKY* genes; Figure S14: Synteny analysis of carrot *DcWRKY* genes; Figure S15: Synteny analysis of sunflower *HaWRKY* genes; Table S3: *WRKY* synteny gene pairs between carrot, globe artichoke, sunflower, and lettuce genomes; Figure S16: Synteny between genomes of carrot (*Daucus carota*) and globe artichoke (*Cynara cardunculus*); Figure S17: Synteny between genomes of carrot (*Daucus carota*) and sunflower (*Helianthus annuus*); Figure S18: Synteny between genomes of sunflower (*Helianthus annuus*) and lettuce (*Lactuca sativa*); Figure S19: Synteny between genomes of sunflower (*Helianthus annuus*) and globe artichoke (*Cynara cardunculus*); Table S4: WRKY coding sequences of globe artichoke, sunflower, lettuce, carrot, ginseng and *P.notoginseng*.

## References

[1] Chen L.G., Xiang S.Y., Chen Y.L., Li D.B., Yu D.Q. (2017). *Arabidopsis* WRKY45 interacts with the DELLA protein RGL1 to positively regulate age-triggered leaf senescence. Mol. Plant.

[2] Xiu H., Nuruzzaman M., Guo X.Q., Cao H.Z., Huang J.J., Chen X.H., Wu K.L., Zhang R., Huang Y.Z., Luo J.L. (2016). Molecular cloning and expression analysis of eight *PgWRKY* genes in *Panax ginseng* responsive to salt and hormones. Int. J. Mol. Sci..

[3] Raineri J., Hartman M.D., Chan R.L., Iglesias A.A., Ribichich K.F. (2016). A sunflower WRKY transcription factor stimulates the mobilization of seed-stored reserves during germination and post-germination growth. Plant Cell Rep..

[4] Liu X.Y., Lv S.S., Liu R., Fan S.X., Liu C.J., Liu R.Y., Han Y.Y. (2018). Transcriptomic analysis reveals the roles of gibberellin-regulated genes and transcription factors in regulating bolting in lettuce (*Lactuca sativa* L.). PLoS ONE.

[5] Wang C.T., Ru J.N., Liu Y.W., Li M., Zhao D., Yang J.F., Fu J.D., Xu Z.S. (2018). Maize WRKY transcription factor ZmWRKY106 confers drought and heat tolerance in transgenic Plants. Int. J. Mol. Sci..

[6] Sun Y.D., Yu D.Q. (2015). Activated expression of AtWRKY53 negatively regulates drought tolerance by mediating stomatal movement. Plant Cell Rep..

[7] Chen M.H., Yan T.X., Shen Q., Lu X., Pan Q.F., Huang Y.R., Tang Y.L., Fu X.Q., Liu M., Jiang W.M. (2017). GLANDULAR TRICHOME-SPECIFIC WRKY 1 promotes artemisinin biosynthesis in *Artemisia annua*. New Phytol..

[8] Eulgem T., Rushton P.J., Robatzek S., Somssich I.E. (2000). The WRKY superfamily of plant transcription factors. Trends Plant Sci..

[9] Xie Z., Zhang Z.L., Zou X.L., Huang J., Ruas P., Thompson D., Shen Q.J. (2005). Annotations and functional analyses of the rice *WRKY* gene superfamily reveal positive and negative regulators of abscisic acid signaling in aleurone cells. Plant Physiol..

[10] Xu W.Q., Choi H.K., Huang L.F. (2017). State of *Panax ginseng* research: A global analysis. Molecules.

[11] Wang T., Guo R.X., Zhou G.H., Zhou X.D., Kou Z.Z., Sui F., Li C., Tang L.Y., Wang Z.J. (2016). Traditional uses, botany, phytochemistry, pharmacology and toxicology of *Panax notoginseng* (Burk.) F.H. Chen: A review. J. Ethnopharmacol..

[12] Que F., Hou X.L., Wang G.L., Xu Z.S., Tan G.F., Li T., Wang Y.H., Khadr A., Xiong A.S. (2019). Advances in research on the carrot, an important root vegetable in the Apiaceae. Hortic. Res..

[13] Lattanzio V., Kroon P.A., Linsalata V., Cardinali A. (2009). Globe artichoke: A functional food and source of nutraceutical ingredients. J. Funct. Foods.

[14] Guo S.S., Ge Y., Na Jom K. (2017). A review of phytochemistry, metabolite changes, and medicinal uses of the common sunflower seed and sprouts (*Helianthus annuus* L.). Chem. Cent. J..

[15] Kim M.J., Moon Y., Tou J.C., Mou B., Waterland N.L. (2016). Nutritional value, bioactive compounds and health benefits of lettuce (*Lactuca sativa* L.). J. Food Compos. Anal..

[16] Yu J., Hu S.N., Wang J., Wong G.K., Li S.G., Liu B., Deng Y.J., Dai L., Zhou Y., Zhang X.Q. (2002). A draft sequence of the rice genome (*Oryza sativa* L. ssp. indica). Science.

[17] International Wheat Genome Sequencing Consortium (IWGSC) (2014). A chromosome-based draft sequence of the hexaploid bread wheat (*Triticum aestivum*) genome. Science.

[18] Jaillon O., Aury J.M., Noel B., Policriti A., Clepet C., Casagrande A., Choisne N., Aubourg S., Vitulo N., Jubin C. (2007). The grapevine genome sequence suggests ancestral hexaploidization in major angiosperm phyla. Nature.

[19] Brand L.H., Fischer N.M., Harter K., Kohlbacher O., Wanke D. (2013). Elucidating the evolutionary conserved DNA-binding specificities of WRKY transcription factors by molecular dynamics and in vitro binding assays. Nucleic Acids Res..

[20] Rice WRKY Working Group (2012). Nomenclature report on rice WRKY’s–Conflict regarding gene names and its solution. Rice.

[21] Dong J.X., Chen C.H., Chen Z.X. (2003). Expression profiles of the *Arabidopsis* WRKY gene superfamily during plant defense response. Plant Mol. Biol..

[22] Wei K.F., Chen J., Chen Y.F., Wu L.J., Xie D.X. (2012). Molecular phylogenetic and expression analysis of the complete WRKY transcription factor family in maize. DNA Res..

[23] Xiao Y., Zhou L.X., Lei X.T., Cao H.X., Wang Y., Dou Y.J., Tang W.Q., Xia W. (2017). Genome-wide identification of WRKY genes and their expression profiles under different abiotic stresses in *Elaeis guineensis*. PLoS ONE.

[24] Li L., Mu S.H., Cheng Z.C., Cheng Y.W., Zhang Y., Miao Y., Hou C.L., Li X.P., Gao J. (2017). Characterization and expression analysis of the *WRKY* gene family in moso bamboo. Sci. Rep..

[25] Guo C.L., Guo R.R., Xu X.Z., Gao M., Li X.Q., Song J.Y., Zheng Y., Wang X.P. (2014). Evolution and expression analysis of the grape (*Vitis vinifera* L.) *WRKY* gene family. J. Exp. Bot..

[26] Tang J., Wang F., Hou X.L., Wang Z., Huang Z.N. (2013). Genome-wide fractionation and identification of WRKY transcription factors in Chinese cabbage (*Brassica rapa* ssp *pekinensis*) reveals collinearity and their expression patterns under abiotic and biotic stresses. Plant Mol. Biol. Rep..

[27] Jiang Y.Z., Duan Y.J., Yin J., Ye S.L., Zhu J.R., Zhang F.Q., Lu W.X., Fan D., Luo K.M. (2014). Genome-wide identification and characterization of the Populus WRKY transcription factor family and analysis of their expression in response to biotic and abiotic stresses. J. Exp. Bot..

[28] Song H., Wang P.F., Lin J.Y., Zhao C.Z., Bi Y.P., Wang X.J. (2016). Genome-wide identification and characterization of *WRKY* gene family in peanut. Front. Plant Sci..

[29] Nan H., Gao L.Z. (2019). Genome-wide analysis of *WRKY* genes and their response to hormone and mechanic stresses in carrot. Front. Genet..

[30] Li M.Y., Xu Z.S., Tian C., Huang Y., Wang F., Xiong A.S. (2016). Genomic identification of WRKY transcription factors in carrot (*Daucus carota*) and analysis of evolution and homologous groups for plants. Sci. Rep..

[31] Jiang C.M., Shen Q.J., Wang B., He B., Xiao S.Q., Chen L., Yu T.Q., Ke X., Zhong Q.F., Fu J. (2017). Transcriptome analysis of *WRKY* gene family in *Oryza officinalis* Wall ex Watt and *WRKY* genes involved in responses to *Xanthomonas oryzae* pv. *oryzae* stress. PLoS ONE.

[32] Silva M.D.A.D., Oliveira J.D.A.D., Del-Bem L.E., Bronze D.S.E., Santana S.R.J., Peres G.K., Vincentz M., Micheli F. (2017). Genome-wide identification and characterization of cacao WRKY transcription factors and analysis of their expression in response to witches’ broom disease. PLoS ONE.

[33] Yang Y., Zhou Y., Chi Y.J., Fan B.F., Chen Z.X. (2017). Characterization of soybean *WRKY* gene family and identification of soybean *WRKY* genes that promote resistance to soybean cyst nematode. Sci. Rep..

[34] Sun W.J., Zhan J.Y., Zheng T.R., Sun R., Wang T., Tang Z.Z., Bu T.L., Li C.L., Wu Q., Chen H. (2018). The jasmonate-responsive transcription factor *CbWRKY24* regulates terpenoid biosynthetic genes to promote saponin biosynthesis in *Conyza blinii* H. Lev. J. Genet..

[35] Suttipanta N., Pattanaik S., Kulshrestha M., Patra B., Singh S.K., Yuan L. (2011). The transcription factor CrWRKY1 positively regulates the terpenoid indole alkaloid biosynthesis in *Catharanthus roseus*. Plant Physiol..

[36] Phukan U.J., Jeena G.S., Shukla R.K. (2016). WRKY transcription factors: Molecular regulation and stress responses in plants. Front. Plant Sci..

[37] Singh A.K., Kumar S.R., Dwivedi V., Rai A., Pal S., Shasany A.K., Nagegowda D.A. (2017). A WRKY transcription factor from *Withania somnifera* regulates triterpenoid withanolide accumulation and biotic stress tolerance through modulation of phytosterol and defense pathways. New Phytol..

[38] Niu C.F., Wei W., Zhou Q.Y., Tian A.G., Hao Y.J., Zhang W.K., Ma B., Lin Q., Zhang Z.B., Zhang J.S. (2012). Wheat *WRKY* genes *TaWRKY2* and *TaWRKY19* regulate abiotic stress tolerance in transgenic *Arabidopsis* plants. PlantCell Environ..

[39] Birkenbihl R.P., Kracher B., Ross A., Kramer K., Finkemeier I., Somssich I.E. (2018). Principles and characteristics of the *Arabidopsis* WRKY regulatory network during early MAMP-triggered immunity. Plant J..

[40] Chakraborty J., Ghosh P., Sen S., Das S. (2018). Epigenetic and transcriptional control of chickpea WRKY40 promoter activity under *Fusarium* stress and its heterologous expression in *Arabidopsis* leads to enhanced resistance against bacterial pathogen. Plant Sci..

[41] Lynch M., Conery J.S. (2000). The evolutionary fate and consequences of duplicate genes. Science.

[42] Yu J., Wang J., Lin W., Li S.G., Li H., Zhou J., Ni P.X., Dong W., Hu S.N., Zeng C.Q. (2005). The genomes of *Oryza sativa*: A history of duplications. PLoS Biol..

[43] Yang X.Z., Li H., Yang Y.C., Wang Y.Q., Mo Y.L., Zhang R.M., Zhang Y., Ma J.X., Wei C.H., Zhang X. (2018). Identification and expression analyses of *WRKY* genes reveal their involvement in growth and abiotic stress response in watermelon (*Citrullus lanatus*). PLoS ONE.

[44] Xie T., Chen C.J., Li C.H., Liu J.R., Liu C.Y., He Y.H. (2018). Genome-wide investigation of *WRKY* gene family in pineapple: Evolution and expression profiles during development and stress. BMC Genom..

[45] Karanja B.K., Fan L.X., Xu L., Wang Y., Zhu X.W., Tang M.J., Wang R.H., Zhang F., Muleke E.M., Liu L.W. (2017). Genome-wide characterization of the *WRKY* gene family in radish (*Raphanus sativus* L.) reveals its critical functions under different abiotic stresses. Plant Cell Rep..

[46] Li W.H., Yang J., Gu X. (2005). Expression divergence between duplicate genes. Trends Genet..

[47] Jiao Z.G., Sun J.L., Wang C.Q., Dong Y.M., Xiao S.H., Gao X.L., Cao Q.W., Li L.B., Li W.D., Gao C. (2018). Genome-wide characterization, evolutionary analysis of *WRKY* genes in Cucurbitaceae species and assessment of its roles in resisting to powdery mildew disease. PLoS ONE.

[48] Xu J., Chu Y., Liao B.S., Xiao S.M., Yin Q.G., Bai R., Su H., Dong L.L., Li X.W., Qian J. (2017). *Panax ginseng* genome examination for ginsenoside biosynthesis. GigaScience.

[49] Chen W., Kui L., Zhang G.H., Zhu S.S., Zhang J., Wang X., Yang M., Huang H.C., Liu Y.X., Wang Y. (2017). Whole-genome sequencing and analysis of the chinese herbal plant *Panax notoginseng*. Mol. Plant.

[50] Iorizzo M., Ellison S., Senalik D., Zeng P., Satapoomin P., Huang J.Y., Bowman M., Iovene M., Sanseverino W., Cavagnaro P. (2016). A high-quality carrot genome assembly provides new insights into carotenoid accumulation and asterid genome evolution. Nat. Genet..

[51] Badouin H., Gouzy J., Grassa C.J., Murat F., Staton S.E., Cottret L., Lelandais-Brière C., Owens G.L., Carrère S., Mayjonade B. (2017). The sunflower genome provides insights into oil metabolism, flowering and Asterid evolution. Nature.

[52] Scaglione D., Reyes-Chin-Wo S., Acquadro A., Froenicke L., Portis E., Beitel C., Tirone M., Mauro R., Lo Monaco A., Mauromicale G. (2016). The genome sequence of the outbreeding globe artichoke constructed de novo incorporating a phase-aware low-pass sequencing strategy of F_1_ progeny. Sci. Rep..

[53] Reyes-Chin-Wo S., Wang Z.W., Yang X.H., Kozik A., Arikit S., Song C., Xia L.F., Froenicke L., Lavelle D.O., Truco M.J. (2017). Genome assembly with in vitro proximity ligation data and whole-genome triplication in lettuce. Nat. Commun..

[54] Cheng C.Y., Krishnakumar V., Chan A.P., Thibaud-Nissen F., Schobel S., Town C.D. (2017). Araport11: A complete reannotation of the *Arabidopsis* thaliana reference genome. Plant J..

[55] Wheeler T.J., Eddy S.R. (2013). nhmmer: DNA homology search with profile HMMs. Bioinformatics.

[56] Chen C.J., Xia R., Chen H., He Y.H. (2018). TBtools, a Toolkit for Biologists integrating various HTS-data handling tools with a user-friendly interface. BioRxiv Bioinf..

