# Supplementary material for: Genome-Wide Identification of WRKY Transcription Factors in the Asteranae
Source: Plants (Basel). 2019 Oct 1;8(10):393. doi: 10.3390/plants8100393 (PMC6843914; doi:10.3390/plants8100393)
Supplement: Supplementary file 1 [file plants-08-00393-s001.zip › Figure S1/Figure S1.pdf]

Group Ⅰa/ Ⅰb

```
                        *        20         *        40         *        60
CcWRKY3     -DGYNWRKYGQKQVKGSDYPRSYYKCTH---PNCLVKKKVERSID-GEIAEIVYKGEHNH-   55
CcWRKY7     -DGYNWRKYGQKQVKSPEGSRSYYKCTY---IECDAKK-IESCDQFNSVTKIVYKGQHKHD   56
CcWRKY9     EDGYNWRKYGQKVVKGSEFPRSYYKCTH---PNCEVKKIFERS-YTGKITEIVYKGTHDHP   57
CcWRKY11    EDGYNWRKYGQKQVKGSEYPRSYYKCTH---PNCLVKKKVERSHE-GHITEIIYKGAHNHP   57
CcWRKY12    EDGYNWRKYGQKQVKGSEYPRSYYKCTH---PNCQVKKKVERSRD-GHITEIIYKGAHNHQ   57
CcWRKY18    DDGYNWRKYGQKQVKGSENPRSYYKCTY---RNCSMRKKVETSAD-GDITEIVYKGNHDHP   57
CcWRKY19    EDGYNWRKYGQKQVKGNEYPRSYYKCTQ---PNCPVKKKVERSQD-GHITEIIYKGAHNHP   57
CcWRKY20    DDGYNWRKYGQKVVKGSEFPRSYYRCTH---PNCEAKKIFECSYT-GQITEIIYKGTHDHP   57
CcWRKY22    NDGYNWRKYGQKQVKASEHPRSYYKCTH---VNCPVKKKVGQSLD-GHISDIVYKGQHNHE   57
CcWRKY25    DDGYVWRKYGDKKILHSKYPRGYYRCSFRYVHNCHATKHIERSIKDPCFFEVSYFGNHTCP   61
CcWRKY26    -DGYNWRKYGQKLVKGNTFVRSYYKCTS---ANCPARKQVERSHD-GHITEINYLWKHEHP   56
CcWRKY30    EDGYNWRKYGQKKVKGGKRPRSYFKCSY---PDCPTRKKVEKDLN-GYITEIVYQGKHSHP   57
CcWRKY33    EDGYNWRKYGQKQVKGSENPRSYYKCTY---PNCSMKKKLETNSD-GQITEIVYKGNHNHP   57
CcWRKY38    EDGYNWRKYGQKQVKGSEYPRSYYKCTN---QNCRVKKKVERNLD-GLVTEIIYNGQHNHQ   57
CcWRKY43    DDGYNWRKYGQKQVKGSEYPRSYYKCTH---PNCPVKKQVERSVE-GLVTEIIYKGQHNHQ   57
CcWRKY57    DDGYNWRKYGQKVVKGSEHPRSYYKCTH---PNCEVKKIFERSYT-GQITEIVYKGTHDHP   57
CcWRKY58    -DGFNWRKYGQKLVRGNVFVRSYYKCTF---ANCPARKQVERSHD-GHITEINYLCKHEHP   56
HaWRKY2     NDGYNWRKYGQKQVKASENPRSYYKCTH---QNCSMRKKVETSSD-GDVTEIVYKGKHNHP   57
HaWRKY3     DDGYKWKKYTHKEVS-SGHKRIHYKCQF---PLCLIKKKVETSSDG-YITRITYHGEHNH-   55
HaWRKY6     EDGYNWRKYGQKQVKGSEYPRSYYKCTH---PSCQVKKKVERSHD-GHVTEIIYKGAHNHQ   57
HaWRKY7     -DGYNWRKYGQKQVKSPPGSRSYFKCTY---NECDAKK-IESCDQHNSVTKIVYKGQHKHE   56
HaWRKY9     DDGYNWRKYGQKVVKGSEHPRSYYKCTH---PNCDVKKIFERSYTTGQITEIVYKGTHDHP   58
HaWRKY27    EDGYNWRKYGQKQVKGSEYPRSYYKCTY---ANCPVTKKVERSHE-GHITEIIYKGAHNHP   57
HaWRKY34    -DSYKWRKYRQKMVKGNSHPRNCYKCAF---AGCTVKKHMEKVVDGSSQVIITYKGLHNHD   57
HaWRKY35    DDGYNWRKYGQKQVKGSEYPRSYYKCTH---PSCPVKKKVERSFE-GHITEIIYKGAHNHP   57
HaWRKY39    -DGYNWRKYGQKQVKGSEHPRSYYKCSN---PNCMVKKKVERSID-GKIAEIVYEGEHNH-   55
HaWRKY41    DDGYNWRKYGQKQVKGSEYPRSYYKCTH---PNCPVKKQVERSVE-GLVTEIIYKGQHNHQ   57
HaWRKY45    EDGYNWRKYGQKQVKGSENPRSYYKCTY---PNCSMKKKLETNIE-GQITEIVYKGNHNHP   57
HaWRKY46    DDGYNWRKYGQKQVKGSEFPRSYYKCTH---ANCPVKKKVERSHE-GHITEIIYKGAHNHP   57
HaWRKY48    -DGYNWRKYGQKQVKSPKGSRSYFKCTY---NNCDAKK-IEACDQYSSVTKIIYKGQHKHE   56
HaWRKY54    NDGYNWRKYGQKQVKASEHLRSYYKCTY---VNCPVKKTVGQSVD-GQITDIVFKGKHNHD   57
HaWRKY62    EDGYNWRKYGQKQVKGSNKPRSYYKCSY---PDCPSKKKVEKDLN-GYIAQVIYKGKHNHP   57
HaWRKY67    -DVYHWRKYGQKEILGSKYQREYYRCSFKHTRGCRALKQVQRTTEHPPVFEVCYIGEHTC-   59
HaWRKY70    -DGYNWRKYGQKLVKGNTFIRSYYKCTF---ASCPARKQVERTHD-GNITEINYLWKHEHP   56
HaWRKY72    -DGYNWRKYGQKLVKGNTFVRSYYKCTF---GNCPARKQVERSND-GIITEINYLWKHEHP   56
HaWRKY84    DDGYNWRKYGQKVVKGSEHPRSYYKCTH---PNCEVKKIFERSYTTGQIMEIVYKGTHDHP   58
HaWRKY94    NDGYKWRKYGQKQVKASELPRSYYKCTH---INCPATKKIGHFLD-GDVSDIIYKGQHNHE   57
HaWRKY97    -DGYNWRKYGQKQVKASEHPRSYYKCTH---PNCPVKKKVGQSLD-GHITDIVYKGQHNHE   56
HaWRKY103   EDGYNWRKYGQKQVKGSENPRSYYKCTY---PNCPTKKKVERNLE-GHITEIVYKGSHTH-   56
HaWRKY110   EDGYNWRKYGQKQVKGSEYPRSYYKCTH---SNCNVKKKVERSHE-GHITEIIYKGAHNHP   57
HaWRKY111   -IKQRWKKYGQKMVKGTPHPRNYYKCTS---AGCTVSKHIEKVIDGSSEVTITYKGVHDHN   57
LsWRKY4     -DGYNWRKYGQKQVKGSEFPRSYYKCTH---PNCVVKKKVERSIN-GEIAETVYKGEHNH-   55
LsWRKY14    -DGYNWRKYGQKQVKASELPRSYYRCTQ---VNCPVTKKIGHFLD-GNTSEIIYSGRHNHE   56
LsWRKY17    EDGYNWRKYGQKQVKGSDYPRSYYRCTH---QRCLVKKKVERSTD-GLVTEIIYKGQHNHQ   57
LsWRKY26    NDGYNWRKYGQKQVKASEHPRSYYKCTH---ANCPVKKKVGQSFD-GHISDIVYKGQHNHD   57
LsWRKY31    -DGYNWRKYGQKLVKGNTFVRSYYKCTF---GNCPARKQVEHSHD-GHITEINYLWKHEHP   56
LsWRKY33    DDGYNWRKYGQKQVKGSENPRSYYKCSD---RNCSMRKKVETSSD-GDITEIVYKGNHNHP   57
LsWRKY42    -DGYNWRKYGQKQVKSPEGSRSYYKCTY---NECEAKK-IESCDQYNSITKIVYKGQHKHE   56
LsWRKY48    EDGYNWRKYGQKQVKGSEYPRSYYKCTH---LNCPVKKKVERSHE-GHITEIIYKGAHNHP   57
LsWRKY49    -DGYNWRKYGQKVVKGSEFPRSYYKCTH---PNCEVKKIFERSCYTGKITEIVYKGTHDHP   57
LsWRKY53    DDGYNWRKYGQKQVKGSEYPRSYYKCTH---PNCPVKKQVERSVE-GQVTEIIYKGQHNHQ   57
LsWRKY54    -DGYNWRKYGQKQVKSPQGSRSYYKCTY---SNCDAKK-MESCDEYNSVTKLVYKGQHKHD   56
LsWRKY57    EDGYNWRKYGQKQVKGSENPRSYYKCTF---PNCPTKKKVERNLE-GHITEIVYKGNHSH-   56
LsWRKY62    DDGYNWRKYGQKQVKGSENPRSYYKCTY---PNCSMKKKLETNLD-GQITEIVYKGNHNHP   57
LsWRKY64    QDGYNWRKYGQKQIKRSNNPQSYYKCTY---PNCPTKKKVEKDLN-GFITKIIYKEKHNH-   56
LsWRKY70    EDGYNWRKYGQKQVKGSEYPRSYYKCTH---PNCQVKKKVERSHD-GHITEIIYKGAHNHQ   57
LsWRKY72    DDGYNWRKYGQKVVKGSEHPRSYYKCTH---PNCEVKKIFERSYT-GQITEIVYKGTHDHP   57
LsWRKY73    -DGFNWRKYGQKLVRDNAFVRSYYKCTY---AKCPARKQVEHSHD-GHIKEINYLCKHQHP   56
DcWRKY16    DDGYNWRKYGQKHVKGSEYPRSYYKCTH---SNCPVKKIVERALD-GQVTEIVYKGQHNHQ   57
DcWRKY28    -DGYNWRKYGQKHVKGSEYPRSYYKCTH---PNCPVKKKVERSLD-GQIAEIVYQGEHHH-   55
DcWRKY32    EDGYNWRKYGQKLVRGNQFVRSYYKCTF---LSCPAKKQVERTHE-GNITNINCRGNHEHP   57
DcWRKY40    DDGYNWRKYGQKLVRGNQFVRSYYKCTF---PSCPAKKQVERSHE-GYITNINCRGNHEHP   57
DcWRKY55    -DGYNWRKYGQKQVKASEYPRSYYKCTY---ANCPVKKKVECSPN-GQVTEITYKGQHNHE   56
DcWRKY58    DDGYNWRKYGQKQVKGTENPRSYYKCTV---LNCPTKKKVETTLD-GHITEIVYKGNHSHP   57
PgWRKY1     EDGYNWRKYGQKQVKGSEYPRSYYKCTH---PNCPVKKKVERSHE-GHVTEIIYKGAHTH-   56
PgWRKY8     -DGYNWRKYGQKQVKSPQGSRSYYRCTY---SQCYAKK-IECCDNSSSVIEIIYKSGHNHD   56
PgWRKY9     DDGYNWRKYGQKQVKGSEYPRSYYKCTY---INCPVKKKVERSFD-GQVTEIIYKGQHNHQ   57
PgWRKY15    -DGYNWRKYGQKQVKASEYPRSYYKCTH---LNCPVKKKVECSPS-GQITEITYKGQHNHE   56
PgWRKY19    EDGYNWRKYGQKQVKGSEYPRSYYKCTH---QNCPVKKKVERSHE-GHITEIIYKGAHNHP   57
PgWRKY28    EDGYNWRKYGQKQVKGSEYPRSYYKCTH---QNCPVKKKVERSHE-GHITEIIYKGAHNHP   57
PgWRKY30    -DGYNWRKYGQKQVKSPQGFRSYYKCTY---SECYAKK-IEFCDSSSSVIEIIYKSHHNHD   56
PgWRKY45    DDGYNWRKYGQKQVKGSENPRSYYKCTF---PNCPTKKKVERTLD-GHITEIVYKGNHNH-   56
PgWRKY50    EDGYNWRKYGQKLVRGNQFIRSYYKCTF---PNCPSKKQVERSHE-GNITNINYRGKHEHP   57
PgWRKY52    EDGFNWRKYGQKQVKGSENPRSYYKCTY---PNCPMKKKVERSIE-GQITEIVYKGSHTHP   57
PgWRKY60    -DGYNWRKYGQKQVKSPQGFRSYYKCTY---SECYAKK-IEFCDSSSSVIEIIYKSHHNHD   56
PgWRKY64    DDGYNWRKYGQKLVKGCEFPRSYYKCTH---PNCEVKKIFERSLD-GQIKEIVYKGTHDHP   57
PgWRKY71    QDGYNWRKYGQKLVRGNQFVRSYYKCTF---PNCPAKKQVERSHE-GNITNINYRGKHEHP   57
PgWRKY74    DDGYNWRKYGQKQVKGSENPRSYYKCTF---PNCPTKKKVESTLD-GHITEIVYKGNHNH-   56
PgWRKY79    DDGYNWRKYGQKQVKGSENPRSYYKCTS---PNCPTKKKVERTLD-GHITEIVYKGNHNHP   57
PgWRKY81    DDGYNWRKYGQKLVKGCEFPRSYYKCTH---PNCEVKKIFERSLD-GQIKEIVYKGTHDHP   57
PgWRKY82    EDGFNWRKYGQKQVKGSENPRSYYKCTY---PNCPMKKKVERSIE-GQITEIVYKGSHTHP   57
PgWRKY105   QDGYNWRKYGQKLVRGNQFVRSYYKCTF---PNCPAKKQVERSHE-GNITNINYRGKHEHP   57
PgWRKY109   -DGYNWRKYGQKQVKASEYPRSYYKCTH---LNCPVKKKVECSPS-GQITEITYKGQHNHE   56
PgWRKY116   DDGYNWRKYGQKQVKGSENPRSYYKCTS---PNCPTKKKVERTLD-GHITEIVYKGNHNHP   57
PgWRKY121   EDGYNWRKYGQKLVRGNQFIRSYYKCTF---PNCPSKKQVERSHE-GNITNINYRGKHEHP   57
PnWRKY1     DDGYNWRKYGQKQVKGSEYPRSYYKCTH---INCPVKKKVERSFD-GQVTEIIYKGQHNHQ   57
PnWRKY3     EDGYNWRKYGQKQVKGSEYPRSYYKCTH---PNCQVKKKVERSHD-GQITEIIYKGAHNHP   57
PnWRKY8     DDGYNWRKYGQKQVKGSENPRSYYKCTS---PNCPTKKKVERTLD-GHITEIVYKGNHNHP   57
PnWRKY18    -DGYNWRKYGQKQVKGSENPRSYYKCTH---PNCPVKKKVEGSLD-GQIAEIVYNGEHSH-   55
PnWRKY24    EDGFNWRKYGQKQVKGSENPRSYYKCTY---PNCPMKKKVERSIE-GQITEIVYKGSHTHP   57
PnWRKY25    EDGYNWRKYGQKQVKGSEYPRSYYKCTH---QNCPVKKKVERSHE-GHITEIIYKGAHNHP   57
PnWRKY34    EDGYNWRKYGQKQVKGSEYPRSYYKCTH---PNCPVKKKVERSHE-GHITEIIYKGAHTH-   56
PnWRKY38    DDGYNWRKYGQKLVKGCEFPRSYYKCTH---PNCEVKKIFERSLD-GQIKEIVYKGTHDHP   57
PnWRKY40    EDGYNWRKYGQKLVRGNQFIRSYYKCTF---PNCPSKKQVERSHE-GNITNINYRGKHEHP   57
PnWRKY44    -DGYNWRKYGQKQVKASEYPRSYYKCTH---LNCPVKKKVECSPS-GQITEITYKGQHNHE   56
PnWRKY51    QDGYNWRKYGQKLVRGNQFVRSYYKCTF---PNCPAKKQVERSHE-GNITNINYRGKHEHP   57
PnWRKY52    DDGYNWRKYGQKQVKGSENPRSYYKCTF---PNCPTKKKVERTLN-GHITEIVYKGNHNH-   56
PnWRKY55    -EGYNWRKYGQKQVKSPQGSRSYYRCTY---SQCYAKK-IECCDNSSSVIEIIYKSGHNHD   56
```

Group Ⅱa

```
                     *        20         *        40         *
CcWRKY16    DGYQWRKYGQKVTRDNPSPRAYFKCSHAPSCPVKKKVQRSVEDQSILVATYEGEHNHP   58
CcWRKY50    DDYQWRKYGQKVTRDNPSPRAYYKCSFAPSCHAKKKVQRSVDDAGVLVATYEGEHNH-   57
CcWRKY53    DGYQWRKYGQKVTRDNPSPRAYYKCSFAPSCPVKKKVQRSVDDAHLLVVTYEGEHNHQ   58
CcWRKY54    DGYQWRKYGQKVTRDNPSPRAYYKCSFAPSCTVKKKVQKSVDDAHLLVVIYEGVHNHE   58
CcWRKY55    DGYQWRKYGQKVTRDNPSPRAYYKCSFAPSCPVKKKVQRSVDNDHLLVVIYEGEHNHE   58
HaWRKY5     DGYQWRKYGQKVTRDNPSPRAYYKCSFAPTCPVKKKVQRSMDDNGVVMVTYEGEHNH-   57
HaWRKY10    DGYHWRKYGQKVTRDNPSPRAYYKCSFAPICPVKKKVQRSVDDAGLLVAVYEGEHNH-   57
HaWRKY56    DGYQWRKYGQKVTRDNPSPRAYFKCSHAPTCPVKKKVQRSVEDQSILVATYEGEHNHP   58
HaWRKY99    DGYQWRKYGQKVTRDNPSPRAYFKCSHAPSCPVKKKVQRDVADQSILVATYEGEHNHP   58
HaWRKY108   DGYQWRKYGQKVTRNNPSPRAYYKCSYAPLCPVKKKVQRSMDDDGLLVATYEGEHNH-   57
LsWRKY11    DGYQWRKYGQKVTRDNPSPRAYYKCSSSPTCPVKKKVQRSVDDPGVVVATYEGEHNH-   57
LsWRKY22    DGYQWRKYGQKVTRDNPSPRAYFKCSHAPTCPVKKKVQRSVEDQSILVATYEGEHNHP   58
LsWRKY28    DGYQWRKYGQKVTRDNPCPRAYYKCSFAPSCPVKKKVQRSVDDEGLLVAIYDGEHNHE   58
DcWRKY34    DGYQWRKYGQKVTRDNPSPRAYFKCSYAPTCPVKKKVQRSIDDQSILVATYEGEHNHP   58
DcWRKY45    DGYQWRKYGQKVTRDNPSPRAYYKCSFAPNCPVKKKVQRSVEDASIIVATYEGEHNH-   57
DcWRKY56    DGYQWRKYGQKVTRDNPCPRAYFKCSFAPTCPVKKKVQRSVEDQSILVATYEGEHNHP   58
PgWRKY22    DGYQWRKYGQKVTRDNPSPRAYFKCSFAPTCPVKKKVQRSIEDQSILVATYEGEHNHP   58
PgWRKY31    DGYHWRKYGQKVTRDNPSPRAYYKCSFAPNCPVKKKVQRSVEDCTMVVATYEGEHNH-   57
PgWRKY35    DGYHWRKYGQKVTRDNPSPRAYYKCSFAPNCPVKKKVQRSVEDCTMVVATYEGEHNH-   57
PgWRKY38    DGYQWRKYGQKVTRDNPSPRAYFKCSFAPTCPVKKKVQRSIEDQSILVATYEGEHNHP   58
PgWRKY77    DGYQWRKYGQKVTRDNPSPRAYFKCSFAPTCPVKKKVQRSIEDQSILVATYEGEHNHP   58
PnWRKY14    DGYQWRKYGQKVTRDNPSPRAYFKCSFAPTCPVKKKVQRSIEDQSILVATYEGEHNHP   58
PnWRKY21    DGYQWRKYGQKVTRDNPSPRAYFKCSFAPTCPVKKKVQRSIEDQSILVATYEGEHNHP   58
```

Group Ⅱb

```
                      *        20         *        40         *       60
CcWRKY4     -DGCQWRKYGQKMAKGNPCPRAYYRCTMA-VGCPVRKQVQRWAEDQTILITTYEGTHNHP   58
CcWRKY10    -DGCQWRKYGQKLAKGNPCPRAYYRCTMA-VGCPVRKQVQRCAEDQTILITTYEGTHNHP   58
CcWRKY21    NDGCQWRKYGQKIAKGNPCPRAYYRCTIS-PTCPVRKQVQRCPQDMSILITTYEGTHNHP   59
CcWRKY27    -DGCQWRKYGQKIAKGNPCPRAYYRCTMS-VGCPVRKQVQRCAEDQTILITTYEGNHNHP   58
CcWRKY48    NDGCQWRKYGQKIAKGNPCPRAYYRCTMS-PSCPVRKHVQRCAEDRSVLITTYEGTHNHP   59
CcWRKY59    -DGCQWRKYGQKMAKGSPFPRAYYRCTMA-VGCPVRKQVQRCAEDKTILITTYEGNHNHP   58
HaWRKY14    -DGCQWRKYGQKMAKGNPCPRAYYRCTMA-VGCPVRKQVQRCVDDQTILITTYEGTHTHP   58
HaWRKY22    -DGCQWRKYGQKMAKGNPCPRAYYRCTMA-TGCPVRKQVQRCAEDRTILITTYEGNHNHP   58
HaWRKY24    -DGCQWRKYGQKMAKGNPCPRAYYRCTMA-VGCPVRKQVQRLAEDQTILVTTYEGTHSHP   58
HaWRKY26    NDGCQWRKYGQKIAKGNPCPRAYYRCTVS-PTCPVRKQVQRCPQDMSILITTYEGTHNHQ   59
HaWRKY65    NDGCQWRKYGQKIAKGNPCPRAYYRCTVS-TNCPVRKQVQRCPQDMSILITTYEGTHNHP   59
HaWRKY66    NDGCQWRKYGQKIAKGNPCPRAYYRCTVA-PGCPVRKQVQRCLEDMSILITTYEGNHNHP   59
HaWRKY71    -DGCQWRKYGQKMAKGNPCPRAYYRCTMA-VGCPVRKQVQRCAEDKTILTTTYEGNHNHP   58
HaWRKY77    -DGCQWRKYGQKMAKGNPCPRAYYRCTMA-VGCPVRKQVQRCAEDRTVLTTTYEGTHNHP   58
HaWRKY81    -DGCQWRKYGQKISKGNPCPRAYYRCTMA-VGCPVKKQVQRCIQDKTILITTYEGNHNHP   58
HaWRKY101   -DGCQWRKYGQKMAKGNPCPRAYYRCTMA-TGCPVRKQVQRCAEDRTILITTYEGNHNHP   58
HaWRKY104   NDGCQWRKYGQKIAKGNPCPRAYYRCTVS-AGCPVRKQVQRCLEDMSILITTYEGNHNHP   59
HaWRKY109   NDGCQWRKYGQKIAKGNPCPRAYYRCTLS-PSCPVRKHVQRCAENRSVLITTYEGTHNHP   59
LsWRKY13    NDGCQWRKYGQKIAKGNPCPRAYYRCTVS-SSCPVRKHVQRCAEDRSVLITTYEGTHNHP   59
LsWRKY21    -DGCQWRKYGQKMAKGNPCPRAYYRCTMA-VGCPVRKQVQRCVDDQTILITTYEGTHNHP   58
LsWRKY27    NDGCQWRKYGQKIAKGNPCPRAYYRCTVS-PTCPVRKQVQRCPQDMSILITTYEGTHNHP   59
LsWRKY30    NDGCQWRKYGQKIAKGNPCPRAYYRCTVA-PGCPVRKQVQRCLEDMSILITTYEGNHNHP   59
LsWRKY32    -DGCQWRKYGQKIAKGNPCPRAYYRCTMA-AGCLVRKQVQRCAQDRSILITTYEGNHNHP   58
LsWRKY38    -DGCQWRKYGQKMAKGNPCPRAYYRCTMA-VGCPVRKQVQRWADDQTILVTTYEGTHNHP   58
LsWRKY46    -DGCQWRKYGQKMAKGNPCPRAYYRCTMA-AGCPVRKQVQRCAEDRTILITTYEGNHNHP   58
LsWRKY47    -DGCQWRKYGQKMAKGNPCPRAYYRCTMV-VGCPVRKQVQRSMDDQTILITTYEGTHNHP   58
LsWRKY74    -DGCQWRKYGQKMAKGNPCPRAYYRCTMA-VGCPVRKQVQRCAEDRTILITTYEGNHNHP   58
DcWRKY4     -DGCQWRKYGQKMAKGNPCPRAYYRCTMA-VGCPVRKQVQRCAEDRTILITTYEGTHNHP   58
DcWRKY15    NDGCQWRKYGQKKAKGNPCPRAYYRCTMS-PSCPV----QRCHENMSVLITTYEGTHNHQ   55
DcWRKY18    -DGCQWRKYGQKMAKGNPCPRAYYRCTMA-GGCPVRKQVQRCAEDRTILITTYEGAHNHP   58
DcWRKY35    NDGCQWRKYGQKIAKGNPCPRAYYRCTVA-PSCPVRKQVQRCSQDMSILISTYEGTHSHP   59
DcWRKY41    -DGCQWRKYGQKMAKGNPCPRAYYRCTMA-AGCPVRKQVQRCAEDRTILITTYEGNHNHP   58
DcWRKY44    -DGCQWRKYGQKIAKGNPCPRAYYRCTMA-TGCPVRKQVQRCADDRSILITTYEGNHNHP   58
DcWRKY46    NDGCQWRKYGQKIAKGNPCPRAYYRCTVA-PGCPVRKQVQRCLEDMSILITTYEGSHNHP   59
DcWRKY49    -DGCQWRKYGQKIAKGNPCPRAYYRCTMA-AGCPVRKQVQRCAEDRSILITTYEGNHNHP   58
DcWRKY57    -DGCQWRKYGQKIAKGNPCPRAYYRCSVT-PSCPVRKQVQRCSQDMSILITTYEGTHNH-   57
DcWRKY65    NDGCQWRKYGQKIAKGNPCPRAYYRCTMS-TSCPVRKHVQRCQEDLSILLTTYEGSHNH-   58
PgWRKY3     -DGCQWRKYGQKIAKGNPCPRAYYRCTMA-AGCPVRKQVQRCAEDRTILITTYEGNHNHP   58
PgWRKY11    -DGCQWRKYGQKMAKGNPCPRAYYRCTMA-AGCPVRKQVQRCAEDRTILITTYEGNHNHP   58
PgWRKY32    NDGCQWRKYGQKIAKENPCPRTYYRCSVA-SSCPVRKQIQRCSDDMSILITTYEGTHNH-   58
PgWRKY51    -DGCQWRKYGQKMAKGNPCPRAYYRCTMA-AGCPVRKQVQRCAEDRTILITTYEGNHNHP   58
PgWRKY56    NDGCQWRKYGQKIAKGNPCPRAYYRCTVA-PTCPVRKQVQRCSDDMSILISTYEGTHNHP   59
PgWRKY75    NDGCQWRKYGQKISKGNPCPRAYYRCTVS-PSCPVRKHVQRCAEDMSILVTTYEGAHNH-   58
PgWRKY83    -DGCQWRKYGQKMAKGNPCPRAYYRCTMA-VGCPVRKQVQRCAEDRTILITTYEGTHNHP   58
PgWRKY90    NDGCQWRKYGQKIAKGNPCPRAYYRCTVA-PACPVRKQVQRCAEDMSILITTYEGTHNHP   59
PgWRKY95    NDGCQWRKYGQKIAKGNPCPRAYYRCTVA-PACPVRKQVQRCAEDMSILITTYEGTHNHP   59
PgWRKY97    -DGCQWRKYGQKIAKGNPCPRAYYRCTMA-AGCPVRKQVQRCAEDRTILITTYEGNHNHP   58
PgWRKY101   NDGCQWRKYGQKIAKGNPCPRAYYRCTVA-PTCPVRKQVQRCSDDMSILISTYEGTHNHP   59
PgWRKY113   -DGCQWRKYGQKMAKGNPCPRAYYRCTMAAVGCPVRKQVQRCSEDRTILITTYEGTHNHP   59
PgWRKY115   -DGCQWRKYGQKMAKGNPCPRAYYRCTMAAVGCPVRKQVQRCAEDRTILITTYEGTHNHP   59
PgWRKY118   NDGCQWRKYGQKIAKGNPCPRAYYRCTVA-PGCPVRKQVQRCLEDMSILVTTYEGTHNHP   59
PgWRKY120   -DGCQWRKYGQKMAKGNPCPRAYYRCTMA-VGCPVRKQVQRCAEDRTILITTYEGTHNHP   58
PnWRKY17    NDGCQWRKYGQKISKGNPCPRAYYRCTVS-PSCPVRKHVQRCAEDMSILVTTYEGAHNH-   58
PnWRKY20    NDGCQWRKYGQKIAKGNPCPRAYYRCSVA-PSCPVRKQIQRCSDDMSILITTYEGTHNHP   59
PnWRKY30    NDGCQWRKYGQKIAKGNPCPRAYYRCTVA-PACPVRKQVQRCAEDMSILITTYEGTHNHP   59
PnWRKY31    -DGCQWRKYGQKMAKGNPCPRAYYRCTMA-VGCPVRKQVQRCAEDRTILITTYEGTHNHP   58
PnWRKY39    -DGCQWRKYGQKMAKGNPCPRAYYRCTMA-AGCPVRKQVQRCAEDRTILITTYEGNHNHP   58
PnWRKY42    -DGCQWRKYGQKIAKGNPCPRAYYRCTMA-AGCPVRKQVQRCAEDRTILITTYEGNHNHP   58
PnWRKY47    NDGCQWRKYGQKIAKGNPCPRAYYRCTVA-PTCPVRKQVQRCSDDMSILISTYEGTHNHP   59
```

Group Ⅱc

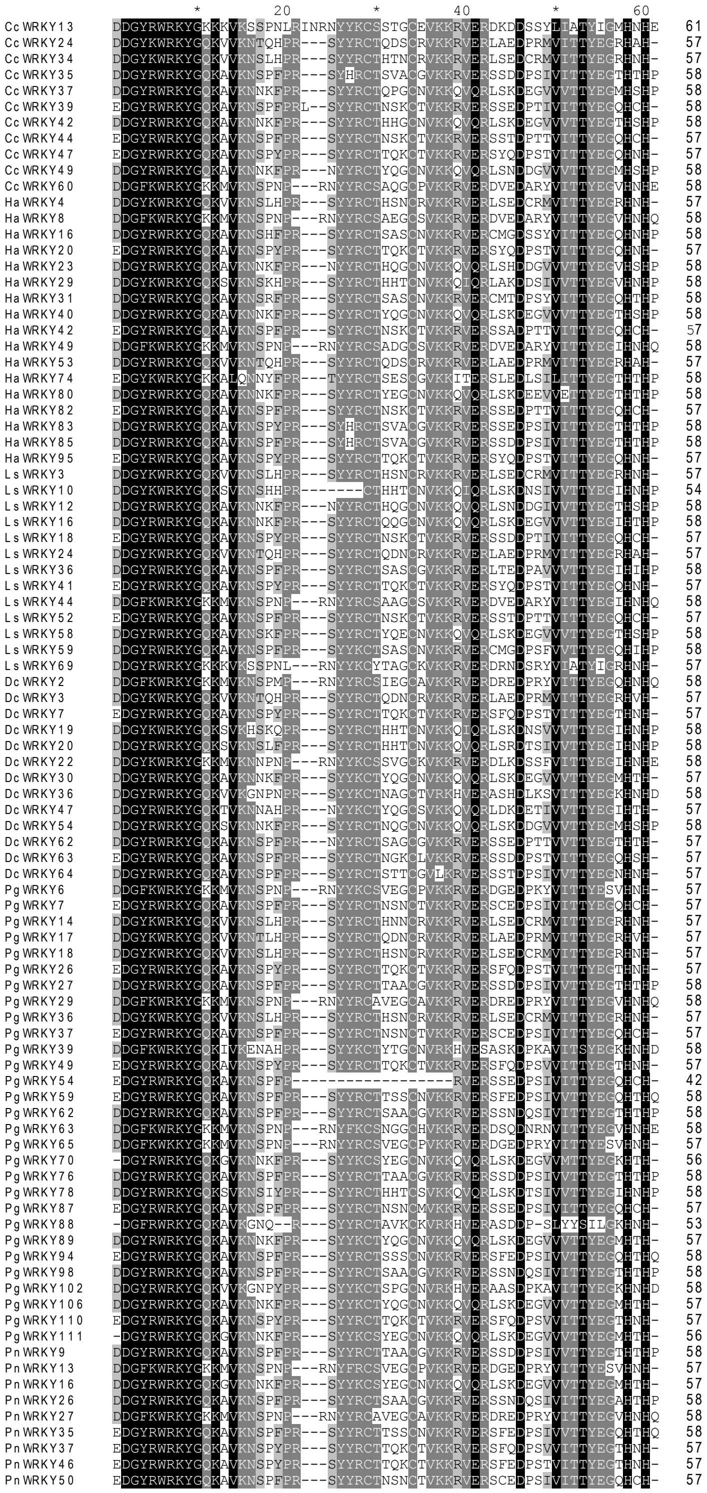

Group Ⅱd

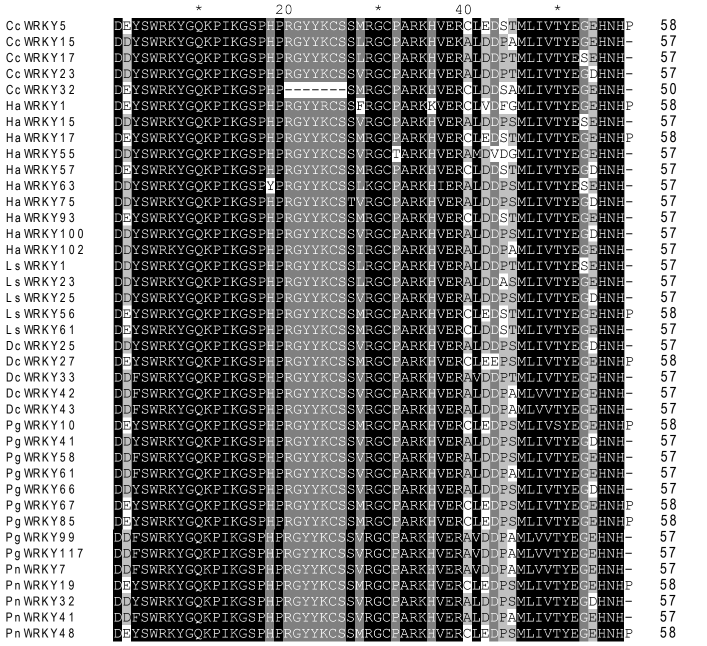

Group III

```
                         *        20         *        40         *        60
CcWRKY1     -DDYEWRKYGQKEILNSQFPRCYFRCTHKDGHGCKALKQVQRLEEELSSKFQITYFGLHTCP   61
CcWRKY2     EDGYTWRKYGQKEILGSKFPRGYFRCTHQKLYNCPAKKQVQRLDNDP-YTFEVTYRGEHTC-   60
CcWRKY14    EDGFAWRKYGQKEILNAKAPRCYFRCTHKNE-GCKALKQVQRLED-GSQMFHITYFGYHTCQ   60
CcWRKY28    DDGHAWRKYGQKKIL----------ECTYKLDQGCLATKQVQKIDNEP-PLYKITYMRSHTC-   51
CcWRKY29    DDGYAWRKYGQKAIHHTNHQRSYYRCTYKFDQGCEATKQVQKTDDEP-SKYKITYNGYHTC-   60
CcWRKY36    DDGYNWRKYGQKLIIGSKYPRSYYRCTYRKAKNCLATKQVQRTDEVP-AVFEIAYKGKHTCN   61
CcWRKY45    DDGYSWSKYGQKDILGAKHPRGYYRCTYRHSEGCLATKQVQRTEDDP-NIFNISYRGSHTCN   61
CcWRKY52    DDGYSWRKYGQKDILGAKFPRSYYRCTYRYVHNCMARKQVQRTDEDP-TVFEITYRGKHAC-   60
CcWRKY56    -DGYSWRKYGQKDILGANHP----RCTHRNFQGCLATKQVQRSDEDS-SVFEVTYKGRHTC-   55
HaWRKY12    DDGYGWRKYGQKTILGAKFPRSYYRCTYRHAQNCMARKQVQRTDEDP-TVFDISYKGQHTC-   60
HaWRKY13    DDGYSWRKYGQKDILGSKFPRSYYRCTYRYIHHCMARKQVQRTNEDP-TVFEITYKGQHSC-   60
HaWRKY30    NDGYSWRKYGQKDILGASHPRAYYRCTHRNVQGCLATKQVQRSDEDS-SVFEVSYRGRHTC-   60
HaWRKY33    NDGYMWRKYGQKEILNAKYHREYYRCTYRNTHGCCATKQVQRSSEDP-STFDITYLGKHTCP   61
HaWRKY36    DDGYNWRKYGQKEIQGAKFPRSYYRCSYRNSQKCFATKQVQRKDEDP-AGFDIVYKGKHTC-   60
HaWRKY37    DDGYNWRKYGQKDIQGAKFPRSYYRCSYRNSQKCFATKQVQRKDEDP-TEFEIAYKGKHTC-   60
HaWRKY38    -DGYNWRKYGQKDILGARFPRSYYRCSYRNAQKCFATKYVQRTDKDP-AEFEIVYKGKHTC-   59
HaWRKY43    EDGYTWRKYGQKDILGSRFPRGYYRCTHQKLYNCPAKKQVQRLDNDP-FTFEVTYRGDHTC-   60
HaWRKY44    EDGYAWRKYGQKEILNSKFPRCYFRCTHKHFHGCKALKQVQKLEEGESNMFHITYFGKHTCP   62
HaWRKY50    EDGYVWRKYGQKEILHAKFPRCYYRCTHKDE-GCKALRQVQQLEEYGSEKYRITYIGSHTC-   60
HaWRKY51    EDGFAWRKYGQKVILDAKFPRCYYRCTHKSE-GCKALKQVQKLED-GSERFHITYFGSHTC-   59
HaWRKY52    EDGFAWRKYGQKVILDAKFPRCYYRCTHKNE-GCKALKQVQKLED-GSERFHITYYGSHTC-   59
HaWRKY59    DDGYAWRKYGQKVILNSKHQRNYFRCSHKFEQGCKATKQVQKTDDEP-SKYKITYIGVHTC-   60
HaWRKY60    DDGYAWRKYGQKVILNSKYQRNYYRCSYKFEQGCQATKQVQMIDDEL-PKYKITYCGHHTC-   60
HaWRKY61    DDGYAWRKYGQKTILNSKHQRNYYRCSHKFEQGCQATKQVQKTDSKP-SNYKITYYGLHTC-   60
HaWRKY64    DDGYAWRKYGQKTILNSKHQRNYFRCSHKFEQGCQATKQVQKINDKP-SKYKITYNGRHTC-   60
HaWRKY76    NDGHMWRKYGQKEILNAKYPREYYRCTYRNTHGCCATKQVQRSSQDP-STFDITYLGKHTCP   61
HaWRKY78    DDGYSWRKYGQKDILGAKFPRSYYRCTYRYIHQCMARKQVQRTDEDP-MVFEITYRGQHTC-   60
HaWRKY86    EDGFAWRKYGQKEILNSKSPRCYFRCTHKHVHGCKAQKQVQKLEDG-SNMYHITYLGKHTCP   61
HaWRKY87    EDGYAWRKYGQKEILNSDFPRCYFRCTHKQVYGCKALKQVQKLKDG-SNMFQIIYFGHHTCP   61
HaWRKY88    EDGYAWRKYGQKEILNSNFPRCYFRCTHKQVHGCKALKQVQKLEDG-SNMFHIIYFGHHTCP   61
HaWRKY89    EDGYSWRKYGQKEILGSKFPRGYFRCTHQKLNNCPAKKQVQRLDNDP-YTFEVTYRGDHTC-   60
HaWRKY90    DDGYAWRKYGEKVIINSKYQRHYYRCTHKFEQGCQATKQVQKTDDEP-PMYRTTYHRHHTC-   60
HaWRKY92    EDGFAWRKYGQKVILDAKFPRCYYRCTHKNE-GCKALKQVQKLED-GSERFHITYYGSHTC-   59
LsWRKY5     EDGYTWRKYGQKEILGSRFPRGYYRCTHQKLYNCPAKKQVQKLDNDP-NTFEVTYRGDHTC-   60
LsWRKY6     EDGYSWRKYGQKEILNSKFPRCYFRCTHKHVLGCKALKQVQKLEDE-TNMLHITYFGYHTC-   60
LsWRKY7     EDGYAWRKYGQKMILNSQSPRCYYRCTHKPDHGCKAQKQVQKLEDE-SNMYHITYFGHHTC-   60
LsWRKY15    -DGYSWRKYGQKDILGANHPRAYYRCTHRHVQGCLATKQVQRSDEDS-SVFEITYRGRHTC-   59
LsWRKY34    NDGYSWRKYGQKDILGAKFPRSYYKCSYRKVQKCLATKQVQKTDEDP-TVFEITYRGIHTC-   60
LsWRKY35    DDGYSWRKYGQKDILGAKFPRSYYRCTYRYVHNCMARKQVQRTDEDP-TVFEITYRGKHSC-   60
LsWRKY45    -----WRKYGQKEILNAKYPREYYRCTYRNTHGCCATKQVQRSSDDS-SIFEITYLGKHTC-   55
LsWRKY50    DDGYNWKKYGQKDILGAKHPRGYYRCTYRQLQGCLATKQVQRTNEDP-NIFNMTYQGIHTC-   60
LsWRKY65    -DGHAWRKYGQKEILNANHQRSYYRCTYKSDQGCLATKQVQMIEDKP-PKYRITYFGNHTC-   59
LsWRKY66    -DGHVWRKYGQKEIINAKHKRNYYRCTHKFDQGCQATKQVQQTEHEP-TKYKITYNRHHIC-   59
LsWRKY67    DDGHAWRKYGQKVILNAKHQRNYYRCTHKTDQGCRATKQVQMTEDEP-PQYKITYSGHHTC-   60
LsWRKY68    -DGHAWRKYGQKHINNTNHQRSYYRCTYKFDQGCLAAKQVQKIQDKP-PKYKTTYMRNHTC-   59
LsWRKY71    EDGYAWRKYGQKEILNAKFPRCYFRCTHKTE-GCKALRQVQKLED-GSQMFHITYYGNHTC-   59
DcWRKY6     -DGYSWRKYGQKDILGAAHPRSYYRCTYRKTRSCQATKQVQRSDNDS-AIFEITYKGKHTC-   59
DcWRKY9     DDGYSWRKYGQKDILGATFPRAYYRCTHRHTQGCLATKQVQQSDEDQ-SVFHITCKGRHTC-   60
DcWRKY21    -DGHSWRKYGQKDILGATFPRAYYRCTYRHSQGCLATKQVQKSDDDS-SLVQVTYKGSHTC-   59
DcWRKY23    DDKYNWRKYGQKDILGAKYPRGYYRCTHRHAQGCLATKQVQRSDNDP-TLFEITYRGRHTC-   60
DcWRKY38    EDGYSWRKYGQKGILNSKYPRCYFRCTYKHEQGCQALKQVQQLEDD-KRMYNITYFGQHTC-   60
DcWRKY50    EDGFTWRKYGQKEILGSRFPRAYYRCTHQKLYNCPAKKQVQRLADDP-FTFEVIYRGDHTC-   60
DcWRKY53    -DGHAWRKYGQKSILNAKYPRHYYRCTHKFEQGCLATKQVQKTEEDPAPMYRTTYYGRHTC-   60
DcWRKY61    EDGYSWRKYGQKDILGATHPRSYYRCTYRNTQMCYATKQVQRSDNDP-TIFEITYKGKHTC-   60
PgWRKY12    -DGYSWRKYGQKDILGARFPRAYYRCTHRHAQGCLATKQVQKSDEDP-SILGITYRGRHTC-   59
PgWRKY16    DDGFSWRKYGQKDILGAKYPRSYYRCTYRNTQTCWATKQVQRSDEDP-ALFEITYKGRHTC-   60
PgWRKY25    DDGFSWRKYGQKDILGAKYPRSYYRCTYRNTQTCWATKQVQRSDEDP-ALFEITYKGRHTC-   60
PgWRKY34    DDGYSWRKYGQKDILGATYPRSYYRCTYRNTQTCCATKQVQRSDDDP-TVFEITYRGKHTC-   60
PgWRKY43    DDGYSWRKYGQKDILGAKYPRGYYRCTNRHIQGCLATKQVQRSDSDP-TIFEITYRGRHTC-   60
PgWRKY46    EDGYTWRKYGQKEILGSRFPRSYYRCTHQKLYQCPAKKQVQRLDHDP-FTFEVNYRGHHKC-   60
PgWRKY47    DDEYAWRKYGQKGILNSKYPRCYFRCTHKHDQGCQALKQVQQFEND-TKMFNITYFGRHTC-   60
PgWRKY72    -DGYSWRKYGQKDILGARFPRAYYRCTYRHAQGCLATKQVQKSDEDP-SILGITYRGRHTC-   59
PgWRKY84    -EGYSWRKYGQKDILGASFPRAYYRCTHRHAQGCLATKQVQKSEENP-SLLEVTYRGRHTC-   59
PgWRKY92    DDEYAWRKYGQKGILNSKYPRCYFRCTHKHDQGCQALKQVQQLEND-TKMFNITYFGRHTC-   60
PgWRKY100   EDGHAWRKYGQKVILNAKYPRNYFRCTHKFDQGCHATKQVQRTGEDP-PIYRTTYHDHHTC-   60
PgWRKY104   -DGYSWRKYGQKDILGASFPRAYYRCTHRHAQGCLATKQVQKSEENP-SLLEVTYRGRHTC-   59
PgWRKY114   DDGYSWRKYGQKDILGATYPRSYYRCTYRNTQTCCATKQVQRSDDDP-TVFEITYRGKHTC-   60
PgWRKY119   EDGYAWRKYGQKVILNAKYPRNYFRCTHKFDQGCHATKQVQRTGEEP-PMYRTTYHGHHTC-   60
PnWRKY4     DDGYSWRKYGQKDILGAKYPRGYYRCTNRHIQGCLATKQVQRSDNDP-TIFEITYRGRHTC-   60
PnWRKY5     DDEYAWRKYGQKGILNSKYPRCYFRCTHKHDQGCQALKQVQQFEND-TKMFNITYFGRHTC-   60
PnWRKY10    -DGYSWRKYGQKDILGASFPRAYYRCTHRHAQGCLATKQVQKSEENP-SLLEATYRGRHTC-   59
PnWRKY12    -DGYSWRKYGQKDILGARFPRAYYRCTHRHAQGCLATKQVQKSDEDP-SILGITYRGRHTC-   59
PnWRKY29    DDGYSWRKYGQKDILGATYPRSYYRCTYRNTQTCCATKQVQRSDDDP-TVFEITYRGKHTC-   60
PnWRKY56    DDGFSWRKYGQKDILGAKYPRSYYRCTYRNTQTCWATKQVQRSDEDP-AVFEITYKGRHTC-   60
```

Group UN

```
                          *        20         *        40         *
CcWRKY8     EDGYNWRKYGQKQVKGSENPRSYYKCTYPNCPTKKKVERNLEGHITEIVYKGNHSH-   56
LsWRKY2     DDGYRWRKYGQRKGNNNRSYYKCVSSNCKVKKTIETNFDGQITKIAYTGSHNHP---   54
DcWRKY52    EDGYNWRKYGQKQVKGSEYPRSYYKCTQQNCQVKKKVERSLDGQITEIIYKGAHNHP   57
PgWRKY42    DGYRWRKYGQKMVKGTPHPRNYYRCTSAGCPVRKHIETANDSRSSVVITYKGIHDH-   56
PgWRKY122   EDGYNWRKYGQKQVKGSEYPRSYYKCTYPNCPVKKKVEGSQEGHITEIIYKGAHTH-   56
```

Group Ⅱe

```
                        *        20         *        40         *        60
CcWRKY6     -DDFSWKKYGEKKIDG-SPYPRVYYKCNTGKGCPARKRVELAIDDPKMLLVTY-DREHIH-   57
CcWRKY31    -DCWSWRKYGQKPIKG-SPYPRGYYRCSTSKSCSAKKQVEICRTDASMLIITY-TSAHNHP   58
CcWRKY40    -DLWSWRKYGQKPIKG-SPYPRGYYKCSTSKGCLARKQVERNRSDPAMLIITY-TGEHTHP   58
CcWRKY41    -DSWAWRKYGQKPIKG-SPYPRGYYRCSSSKGCPARKQVERSRADPTMVMVTY-SCDHNHP   58
CcWRKY46    -DSWAWRKYGQKPIKG-SPYPRGYYRCSSSKGCPARKQVERSRTDPTMVMVTY-SCEHNHP   58
CcWRKY51    -DPWAWRKYGQKPIKG-SIYPRSYYRCSSSKGCMARRQVEQSCTDPSIYILTY-TADHNH-   57
HaWRKY11    -DDYSWKKYGEKKIDG-SLYPRVYYKCITGKGCPARKRVELSADDSKMLIVTY-DGEHRH-   57
HaWRKY18    -DSWAWRKYGQKPIKG-SPYPRGYYRCSSSKGCPARKQVERSRADPTMVMVTY-SCEHNHP   58
HaWRKY19    -DLWAWRKYGQKPIKG-SPYPRGYYRCSSSKGCSARKQVERSRTDPNMLVITY-TSEHNHP   58
HaWRKY21    -DVWSWRKYGQKPIKG-SPYPRGYYRCSTSKGCLARKQVERNRSDPDMFIVTY-TGDHSHP   58
HaWRKY25    -DDYSWKKYGEKRIDG-SPNPRVYYKCSTGKGCPARKCVELAVDDSKMLIVTY-AGEHVH-   57
HaWRKY28    -DPWAWRKYGQKPIKG-SIYPRSYYRCSSSKGCMARRQVEQSCTDSSIYILTY-TADHNH-   57
HaWRKY32    -DVWSWRKYGQKPIKG-SPYPRGYYRCSTSKGCLARKQVERNRSDPDMFIVTY-TGEHSHP   58
HaWRKY47    -DDYSWKKYGEKKIDG-SSYPRVYYKCSSGKGCRARKRVELAADDAKMLLVTY-DGEHNH-   57
HaWRKY58    -DCWSWRKYGQKPIKG-SPYPRGYYKCSTSKSCSAKKQVEICRTDSTMLIITY-TSTHNHP   58
HaWRKY68    -DSWAWRKYGQKPIKG-SPYPRGYYRCSSSKGCPARKQVERNRADPTMVIVTY-SCDHNHP   58
HaWRKY69    NDGWAWRKYGQKPIKG-SPYPRNYYRCSTTKDCSARKQVERSPVNPSSFIVSY-SGDHLHP   59
HaWRKY73    -DDYSWKKYGEKKVTG-SPFPRVYYKCNSGEGCPARKRVELSLTDSKMLIVTY-DREHRH-   57
HaWRKY79    -DDYSWKKYGEKKIEG-SRFPRVYYKCNSVKGCPARKRVELSVTDSKMLVVTY-DGEHRH-   57
HaWRKY91    -DCWSWRKYGQKPIKG-SPHPRGYYKCSTSKSCLAKKQVEICRTDASMLIVTY-TSTHNHP   58
HaWRKY96    -DSWAWRKYGQKPIKG-SPYPRGYYRCSSSKGCPARKQVERSRADPTMVMVTY-SCEHNHP   58
HaWRKY98    DDGYKWRKYGQKSIKN-SANPRNYYKCTNPR-CGAKKQVERSNDDPDTLIITY-EGLHLH-   57
HaWRKY105   -DSWAWRKYGQKPIKG-SPYPRGYYRCSSSKGCPARKQVERNRADPTMVIVTY-SCDHNH-   57
HaWRKY106   -DLWSWRKYGQKPIKG-SPYPRGYYKCSTSKGCLARKQVERNKSDPGMLIITY-TGEHNHP   58
HaWRKY107   -DPWAWRKYGQKPIKG-SIYPRSYYRCSSSKACMARRQVEQSCTDSSTYILTY-TAEHNHP   58
HaWRKY112   -DLWSWRKYGQKPIKG-SPYPRGYYKCSTLKGCSARKQVERNRSDPGMLIITY-TGEHTHP   58
LsWRKY8     NDAWAWRKYGQKPIKG-SPFPR----CSTTKACGAKKQVERSPVDATIFIVSY-SGEHIHP   55
LsWRKY9     -DPWAWRKYGQKPIKG-SIYPRSYYRCSSSKGCMARRQVEQSCTDPSIYILTY-TAEHNHP   58
LsWRKY19    -DLWSWRKYGQKPIKG-SPYPRGYYRCSTSKGCMARKQVERNRSDPGMLIITY-TGEHSH-   57
LsWRKY20    -DSWAWRKYGQKPIKG-SPYPRGYYRCSSSKGCPARKQVERSRTDPTMVMVTY-ACEHNHP   58
LsWRKY29    -DDYSWKKYGEKKIEG-LPFPRVYYKCNTVKGCPARKRIELSLTDSKMLLVTY-DREHRH-   57
LsWRKY37    -DIWSWRKYGQKPIKG-SPYPRGYYRCSTSKGCLARKQVERNRSDPDMFIVTY-TGEHSHP   58
LsWRKY39    -DSWAWRKYGQKPIKG-SPYPRGYYRCSSSKGCPARKQVERSRTDPTMVMVTY-SCEHNH-   57
LsWRKY40    -DLWAWRKYGQKPIKG-SPYPRGYYRCSSSKGCSARKQVERSRTDPNMLVITY-TSEHNHP   58
LsWRKY43    -DDYSWKKYGEKKIDG-SNYPRVYYKCNTGKGCPARKRVELALDDSKMLLVTY-DGEHRH-   57
LsWRKY51    -DTWAWRKYGQKPIKG-SPFP------STTKACGARKQVEQSHVDPTVFIVSY-SGEHMHP   52
LsWRKY55    -DDFSWKKYGEKSVDG-SRYPRVYYKCNTGKGCPARKSVELALDDSKMLLVTY-DGEHIH-   57
LsWRKY60    -DCWSWRKYGQKPIKG-SPYPRGYYRCSTSKSCSAKKQVELCRTDASMLIITY-TSTHNHP   58
LsWRKY63    DDGYKWRKYGQKSIKN-SSNPRSYYKCTNPR-CGAKKQVERSNDDPDTLIITY-EGLHLH-   57
DcWRKY1     -DLWAWRKYGQKPIKG-SPYPRSYYRCSSSKGCPARRQVEESCSDPGMYIITY-SAEHNH-   57
DcWRKY5     -DLWAWRKYGQKPIKG-SPYPRSYYRCSSSKGCLARKQVEESCSSPGTFIMTY-SAEHNH-   57
DcWRKY8     -DSWAWRKYGQKPIKG-SPYPRAYYRCSSSKGCLARKQVERNRADPTMLMVTY-SCEHNHP   58
DcWRKY10    -DCWAWRKYGQKPIKG-SPYPRSYYKCSSSKGCLARKQVEQSCTDPGMFIITYNSAEHNH-   58
DcWRKY11    -DLWAWRKYGQKPIKG-SPYPRGYYRCSSSKGCSARKQVERSRTDPNMLVITY-TSEHNHP   58
DcWRKY12    -DCWSWRKYGQKPIKG-SPYPRGYYKCSTSKSCSAKKQVERCKTDASLLIITY-TSTHNHQ   58
DcWRKY13    -DEYSWRKYGQKPVKG-SPHPRAYYKCTSMRNCPARKKVERCLEEPSMLTITY-ENEHNHP   58
DcWRKY14    -DMWSWRKYGQKPIKG-SPYPRGYYRCSTSKGCLARKQVERNRSDPTMFIVTY-TAEHNHP   58
DcWRKY17    -DLWAWRKYGQKPIKG-SPYPRGYYRCSSSKGCSARKQVERSRTDPNMLVITY-TSEHNHP   58
DcWRKY24    -DTWAWRKYGQKPIKG-SPYPRNYYRCSTLKGCPARKQVEGSPTDATIFIVSY-TGEHAHP   58
DcWRKY26    -DSWSWRKYGQKPIKG-SPYPRGYYRCSTSKGCLAKKQVERCRTDASLLIITY-TSSHNHP   58
DcWRKY29    -DKYSWKKYDQKLIPG-TLYPRGYYKCNSASGCPARKHVERDSTDPTVLILIY-EGEHRH-   57
DcWRKY31    -DSWAWRKYGQKPIKG-SPYPRGYYRCSSSKGCPARKQVERSRKDPTVVVITY-VSEHNH-   57
DcWRKY37    -DEYSWKKYDQKLVPG-TVFPRGYYKCNTFKGCPARKRVERTSNDPTVLILTY-EGEHRH-   57
DcWRKY39    -DAWTWRKYGQKPIKG-SPYPRGYYRCSTSKGCLAKKQVERCRTDASLLIITY-TSSHNH-   57
DcWRKY48    -DSWTWRKYGQKPIKG-SPFPRGYYRCSSSKGCPARKQVERSLKDPTFIVITY-ACDHNH-   57
DcWRKY51    -DEYSWKKYDHKSITGSSLRSGGYYKCNSVTGCSARKHVIKDKNDSMVLIVTY-KGEHNH-   58
DcWRKY59    DDGYKWRKYGQKSIKN-SSNPRSYYRCTNPH-CSAKKQVERSTDDPDTIIITY-EGLHLH-   57
DcWRKY60    -DSWAWRKYGQKPIKG-SPYPRGYYRCSSSKGCPARKQVERSRADPTMLMVTY-SSEHNHP   58
PgWRKY2     EDGFEWRKYGQKFIKN-IGKTRSYFKCRKSN-CIAKKKVEWSVSEPGNLKIVY-EAEHNH-   57
PgWRKY4     -DLWAWRKYGQKPIKG-SPYPRSYYRCSSLKGCLARKQVEQSCNDPGMFIITY-SGEHSH-   57
PgWRKY5     -DSWAWRKYGQKPIKG-SPYPRGYYRCSSSKGCPARKQVERSRADPTMLMVTY-SCEHNHP   58
PgWRKY13    DDGYKWRKYGQKSIKN-SSNPRSYYKCTNPR-CSAKKQVQRSTDDPDTLIITY-EGLHLH-   57
PgWRKY20    -DMWSWRKYGQKPIKG-SPYPRSYYRCSTSKSCLARKKVERNRSDPNMFIVTY-SQEHNHP   58
PgWRKY21    -DMWSWRKYGQKPIKG-SPYPRGYYRCSTSKGCLARKQVERNRSDPNMFIVTY-TAEHNHP   58
PgWRKY23    -DLWAWRKYGQKPIKG-SPYPRGYYRCSSSKGCSARKQVERSRTDPNMLVITY-TSEHNHP   58
PgWRKY24    -DMWSWRKYGQKPIKG-SPYPRGYYRCSTSKGCLARKQVERNRSDPNMFIVTY-TAEHNHP   58
PgWRKY33    -DSWAWRKYGQKPIKG-SPYPRGYYRCSSSKGCPARKQVERSRADPTMLMVTY-SCEHNHQ   58
PgWRKY40    -DMWSWRKYGQKPIKG-SPYPRSYYRCSTSKSCLARKKVERNRSDPNMFIVTY-SQEHNHP   58
PgWRKY44    -DLWAWRKYGQKPIKG-SPYPRGYYRCSSSKGCSARKQVERSRTDPNMLVITY-TSEHNHP   58
PgWRKY48    -DSWAWRKYGQKPIKG-SPNPRGYYRCSSSKGCPARKQVERSRKDPTVVVITY-ACEHNH-   57
PgWRKY53    -DSWAWRKYGQKPIKG-SPNPRGYYRCSSSKGCPARKQVERSRKDPTVVVITY-ACEHNH-   57
PgWRKY57    -DSWAWRKYGQKPIKG-SPYPRGYYRCSSSKGCPARKQVERSRADPTTLLVTY-SCDHNHP   58
PgWRKY68    -DLWAWRKYGQKPIKG-SPYPRGYYRCSSSKGCSARKQVERSRTDPNMLVITY-TSEHNHP   58
PgWRKY69    -DLWAWRKYGQKPIKG-SPYPRGYYRCSSSKGCSARKQVERSRTDPNMLVITY-TSEHNHP   58
PgWRKY73    -DSWAWRKYGQKPIKG-SPYPRNYYRCSTSKGCAARRQVERSPIDGTILIVSY-TGEHTHP   58
PgWRKY80    -DEYSWRKYDQKLIPG-TLFPRGYYKCNSVKGCPARKHAVRSQDDPTVLVVTY-EGEHRHN   58
PgWRKY86    -DLWAWRKYGQKPIKG-SPYPRSYYRCSSSKGCLARKQVEQSCTDPGMFIINY-SGEHNH-   57
PgWRKY91    -DCWSWRKYGQKPIKG-SPYPRGYYKCSTSKGCSAKKQVERCRTDASLLIVTY-TSTHNHP   58
PgWRKY93    -DCWSWRKYGQKPIKG-SPYPRGYYKCSTSKGCSAKKQVERCRTDASLLIVTY-TSTHNHP   58
PgWRKY96    -DCWSWRKYGKKPIKG-SPYPRGYYRCSTSKGCSAKKQVERCRTDASLLIITY-TSTHNHP   58
PgWRKY103   -DLWAWRKYGQKPIKG-SPYPRSYYRCSSLKGCLARKQVEQSCNDPGMFIITY-SGEHSH-   57
PgWRKY107   -DSWAWRKYGQKPIKG-SPYPRGYYRCSSSKGCPARKQVERSRADPTTLLVTY-SCDHNHP   58
PgWRKY108   DDGYKWRKYGQKSIKN-SSNPRSYYKCTNPR-CSAKKQVQRSTDDPDTLIITY-EGLHLH-   57
PgWRKY112   -DEYSWKKYDQKLIPG-TLFPRGYYKCNSIKGCPARKKVERARNDPTALIVTY-EGDHRH-   57
PgWRKY123   -DCWSWRKYGQKPIKG-SPYPRGYYRCSTSKGCSAKKQVERCRTDASLLIITY-TSTHNHP   58
PnWRKY2     EDGFEWRKYGQKFIKN-IGKTRSYFKCQKSN-CIAKKKVEWSVSEPGNLKIVY-EAEHNH-   57
PnWRKY6     -DCWSWRKYGQKPIKG-SPYPRGYYKCSTSKGCSAKKQVERCRTDASLLIVTY-TSTHNHP   58
PnWRKY11    -DLWAWRKYGQKPIKG-SPYPRGYYRCSSSKGCSARKQVERSRTDPNMLVITY-TSEHNHP   58
PnWRKY15    -DSWAWRKYGQKPIKG-SPYPRGYYRCSSSKGCPARKQVERSRADPNTLLVTY-SCDHNHP   58
PnWRKY22    -DMWSWRKYGQKPIKG-SPYPRSYYRCSTSKSCLARKKVERNRSDPNMFIVTY-SQEHNHP   58
PnWRKY23    -DEYSWKKYDQKLIPG-TLFPRGYYKCNSIKGCPARKKVERAGNDPTVLIVTY-EGDHRH-   57
PnWRKY28    -DLWAWRKYGQKPIKG-SPYPRGYYRCSSSKGCSARKQVERSRTDPNMLVITY-TSEHNHP   58
PnWRKY33    -DLWSWRKYGQKPIKG-SPYPRSYYRCSSLKGCLARKQVEQSCNDPGMFIITY-SGEHSHN   58
PnWRKY36    -DSWAWRKYGQKPIKG-SPYPRGYYRCSSSKGCPARKQVERSRADPTMLMVTY-SCEHNHP   58
PnWRKY43    -DEYSWRKYYQKLIPG-TLFPRGYYKCSSVKGCPARKHAVRSQDDPTVLVVTY-EGEHRHN   58
PnWRKY45    -DCWSWRKYGQKPIKG-SPYPRGYYKCSTSKGCSAKKQVERCRTDASLLIITY-TSTHNHP   58
PnWRKY49    DDGYKWRKYGQKSIKN-SSNPRSYYKCTNPR-CSAKKQVQRSTDDPDTLIITY-EGLHLH-   57
PnWRKY53    -DSWAWRKYGQKPIKG-SPNPRGYYRCSSSKGCPARKQVERSRKDPTVVVITY-ACEHNH-   57
PnWRKY54    -DMWSWRKYGQKPIKG-SPYPRGYYRCSTSKGCLARKQVERNRSDPNMFIVTY-TAEHNHP   58
```
