# Supplementary figures and images for: Genome-Wide Identification of WRKY Transcription Factors in the Asteranae
Source: Plants (Basel). 2019 Oct 1;8(10):393. doi: 10.3390/plants8100393 (PMC6843914; doi:10.3390/plants8100393)

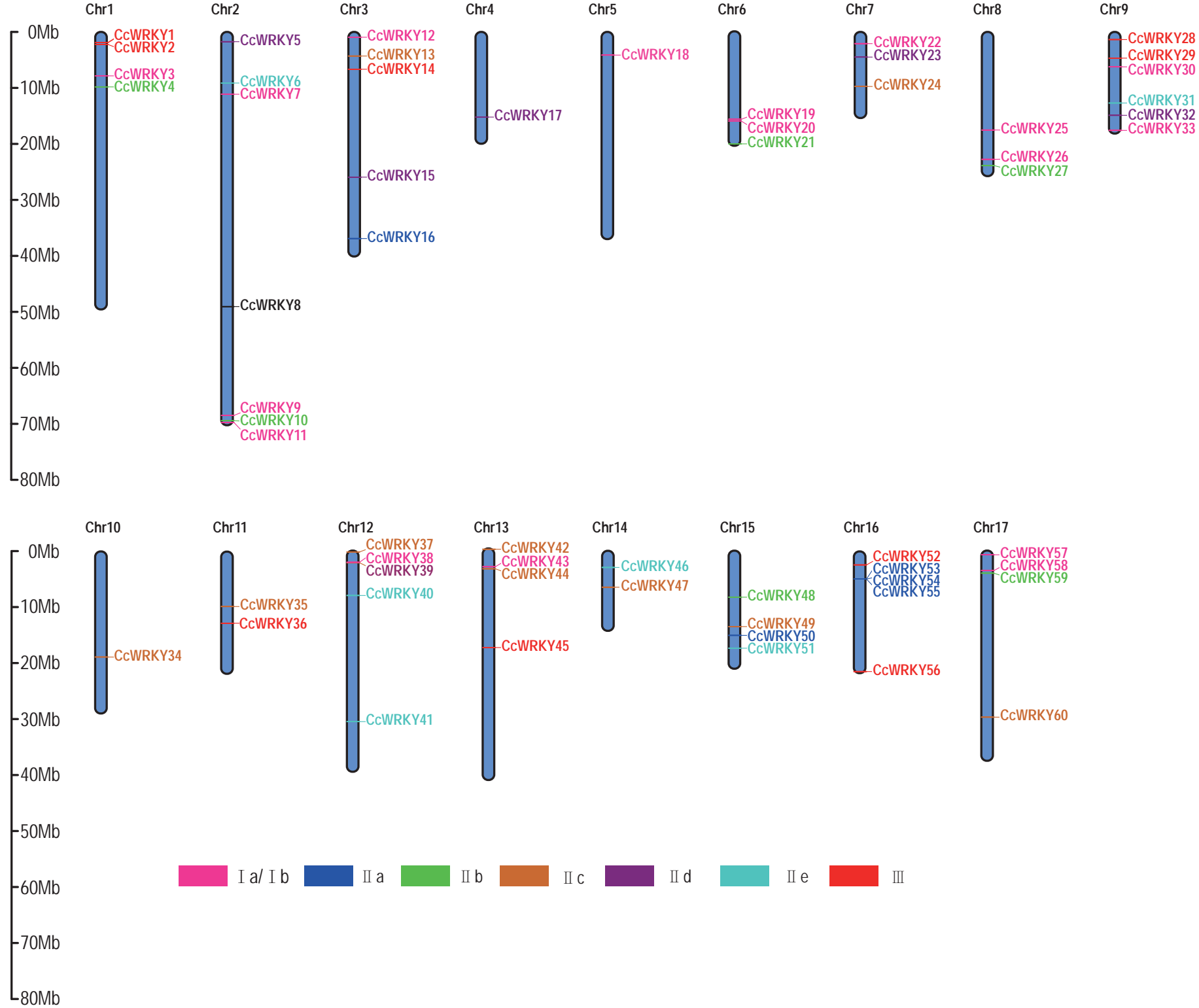

Supplement: Supplementary file 1 [file plants-08-00393-s001.zip › Figure S10/Figure S10.pdf]

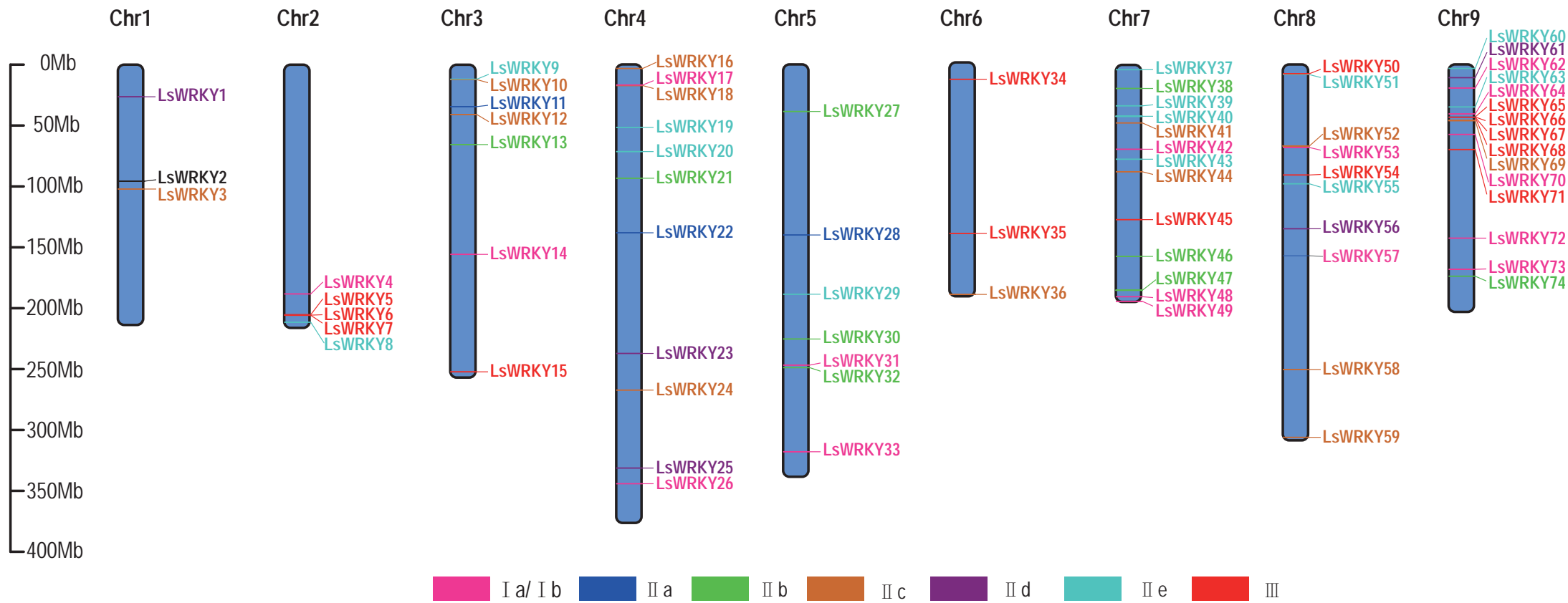

Supplement: Supplementary file 1 [file plants-08-00393-s001.zip › Figure S11/Figure S11.pdf]

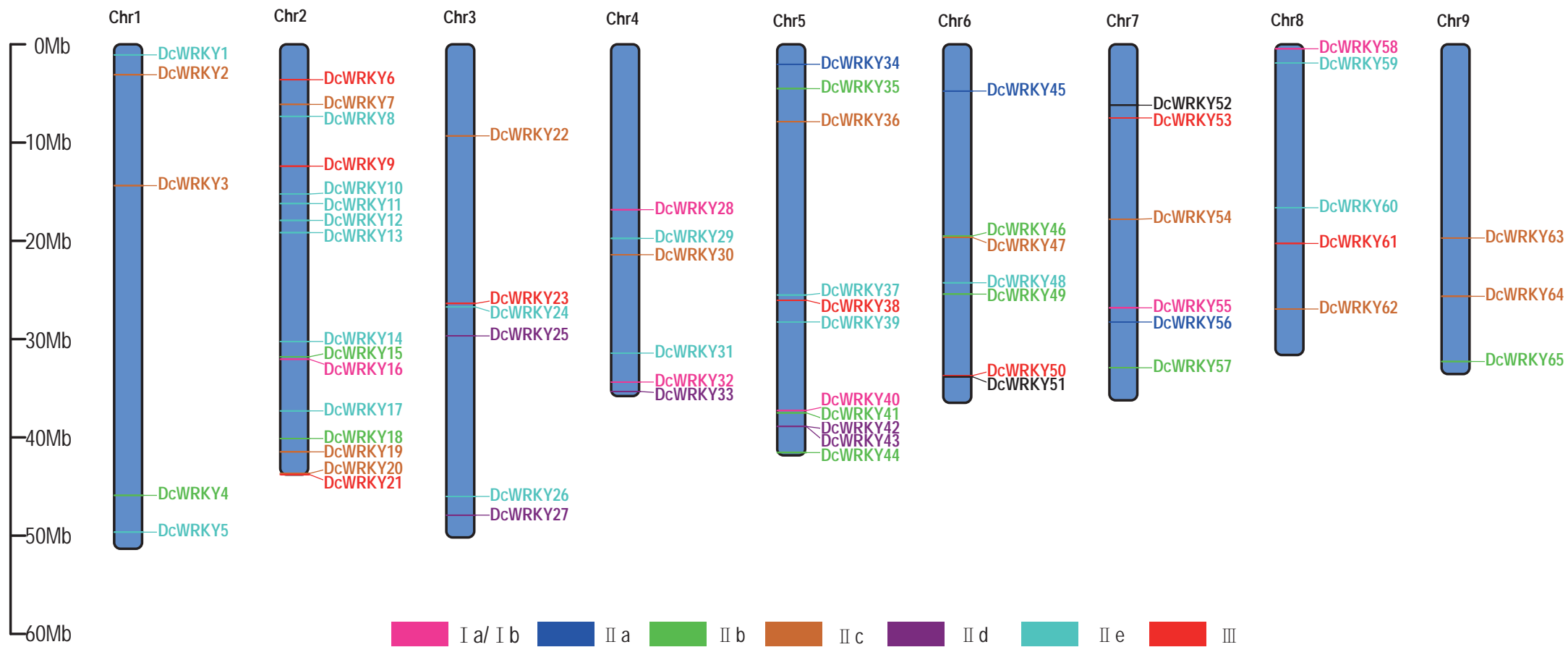

Supplement: Supplementary file 1 [file plants-08-00393-s001.zip › Figure S12/Figure S12.pdf]

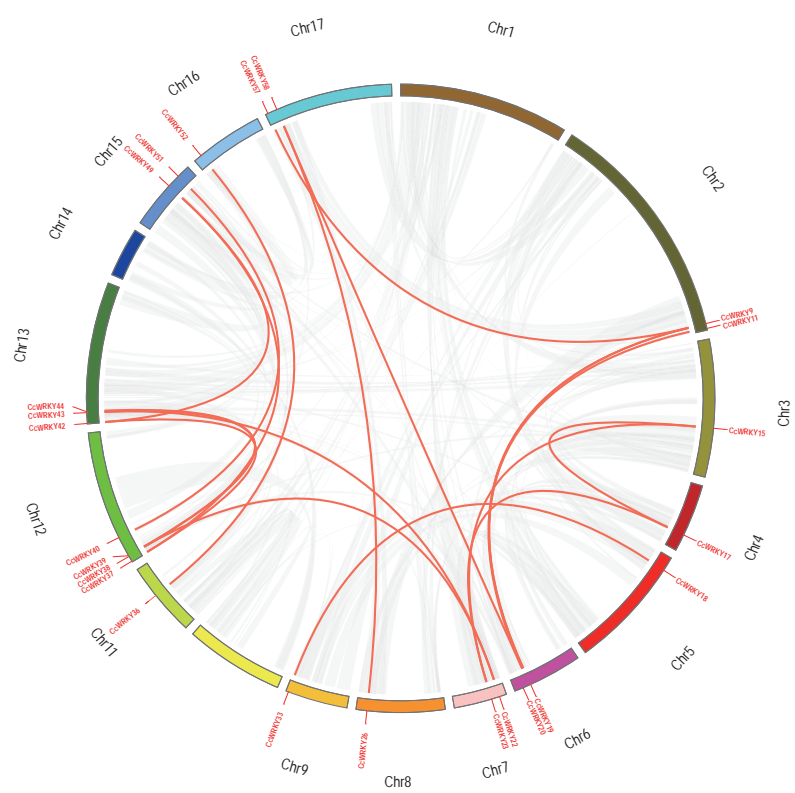

Supplement: Supplementary file 1 [file plants-08-00393-s001.zip › Figure S13/Figure S13.pdf]

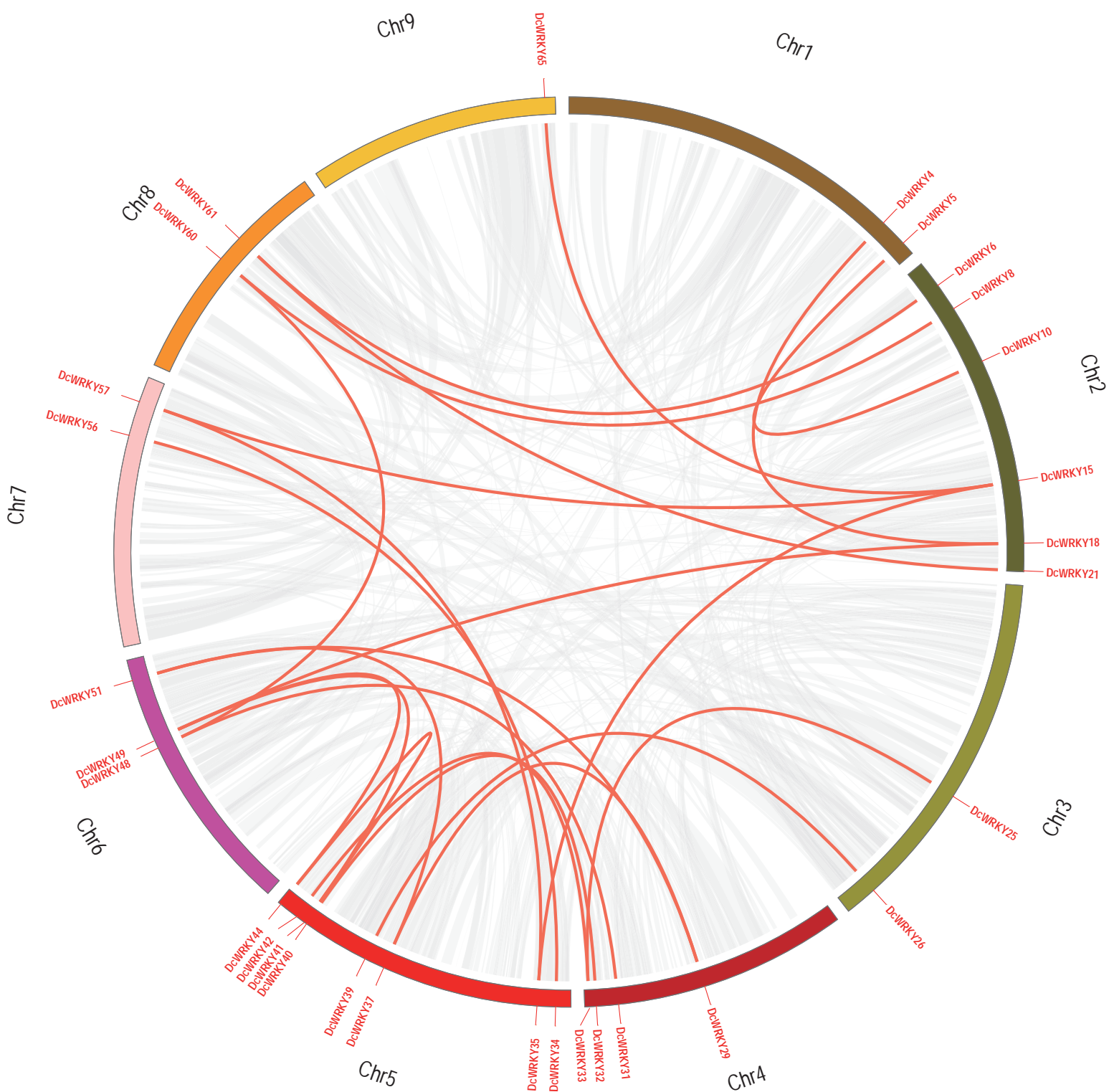

Supplement: Supplementary file 1 [file plants-08-00393-s001.zip › Figure S14/Figure S14.pdf]

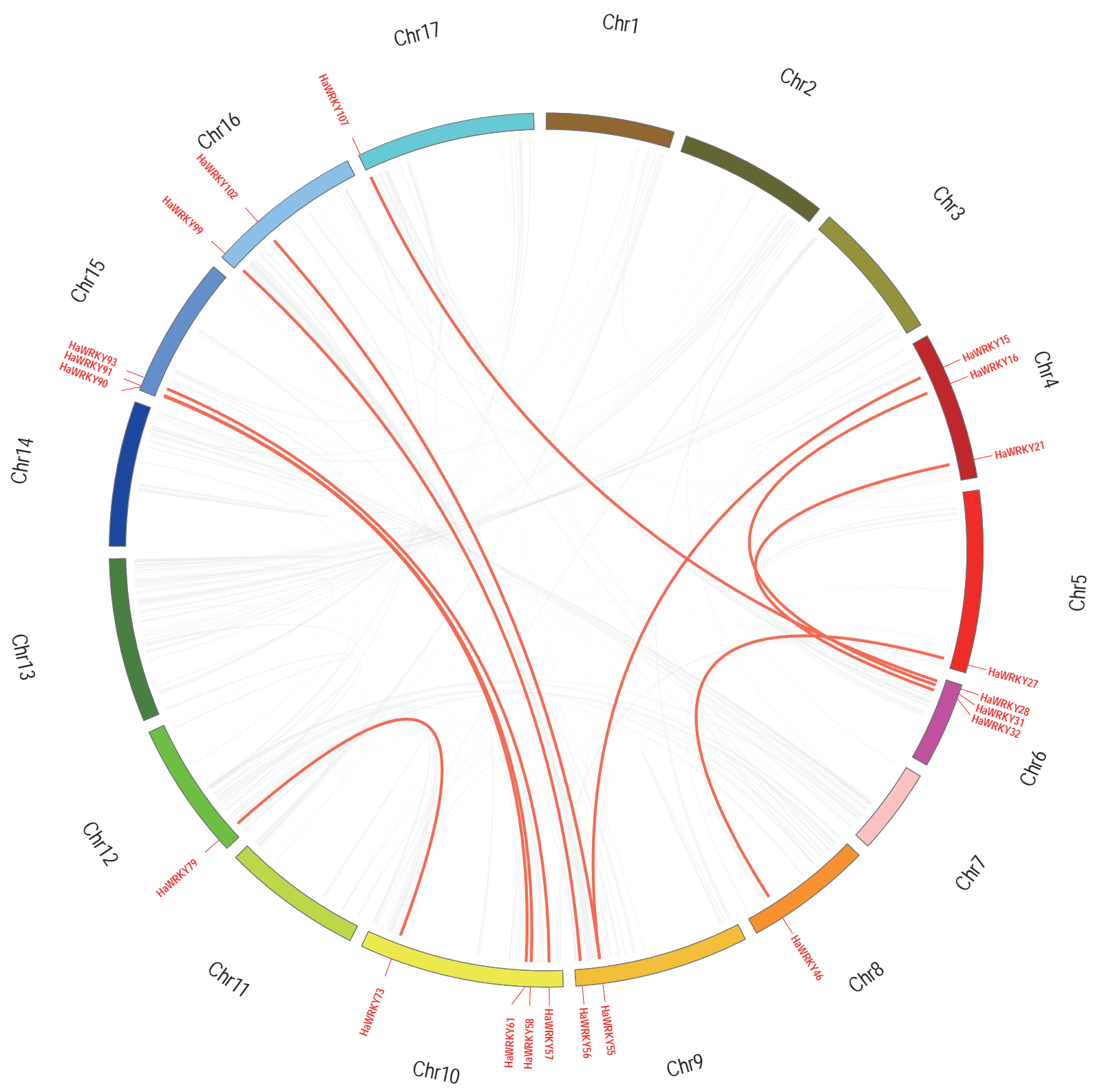

Supplement: Supplementary file 1 [file plants-08-00393-s001.zip › Figure S15/Figure S15.pdf]

*D.carota*

*C.cardunculus*

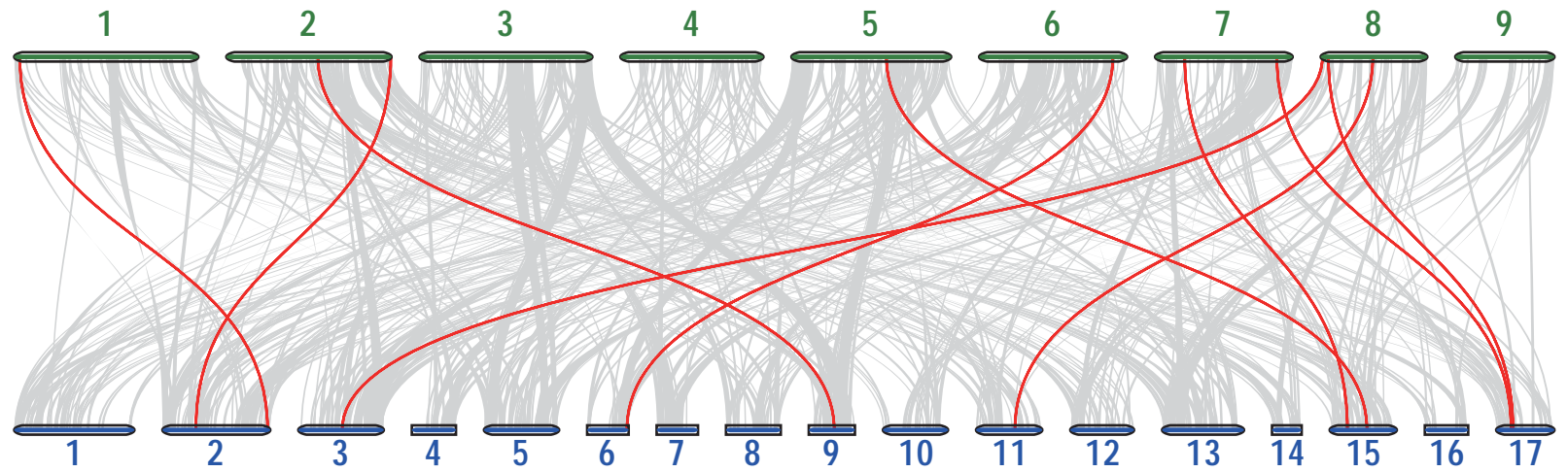

Supplement: Supplementary file 1 [file plants-08-00393-s001.zip › Figure S16/Figure S16.pdf]

*D.carota*

*H.annus*

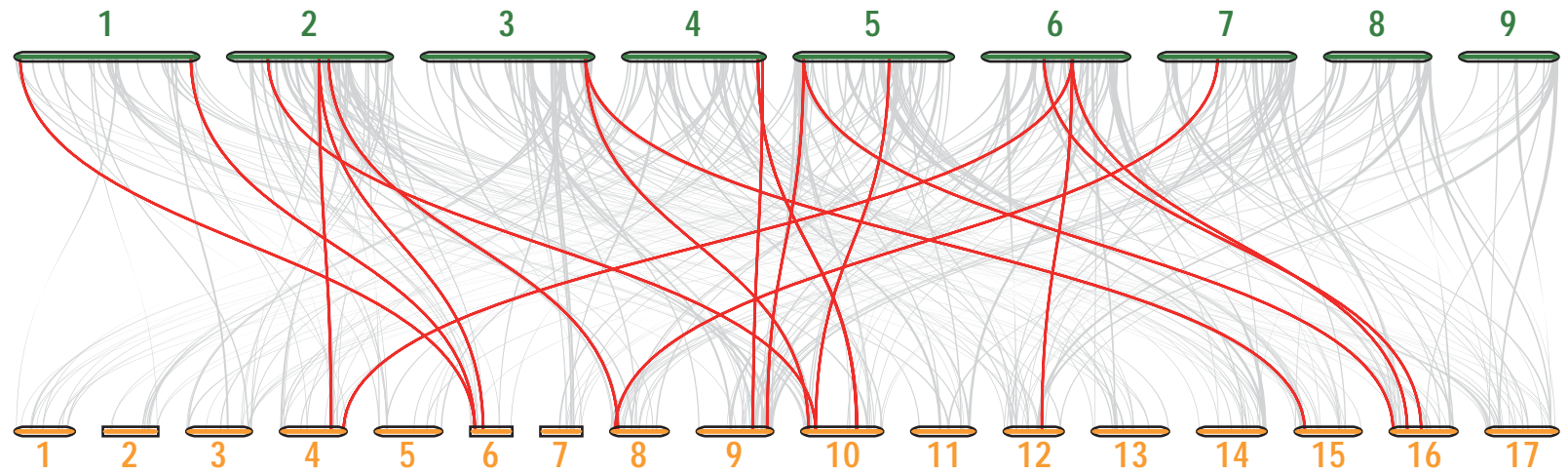

Supplement: Supplementary file 1 [file plants-08-00393-s001.zip › Figure S17/Figure S17.pdf]

*H. annuus*

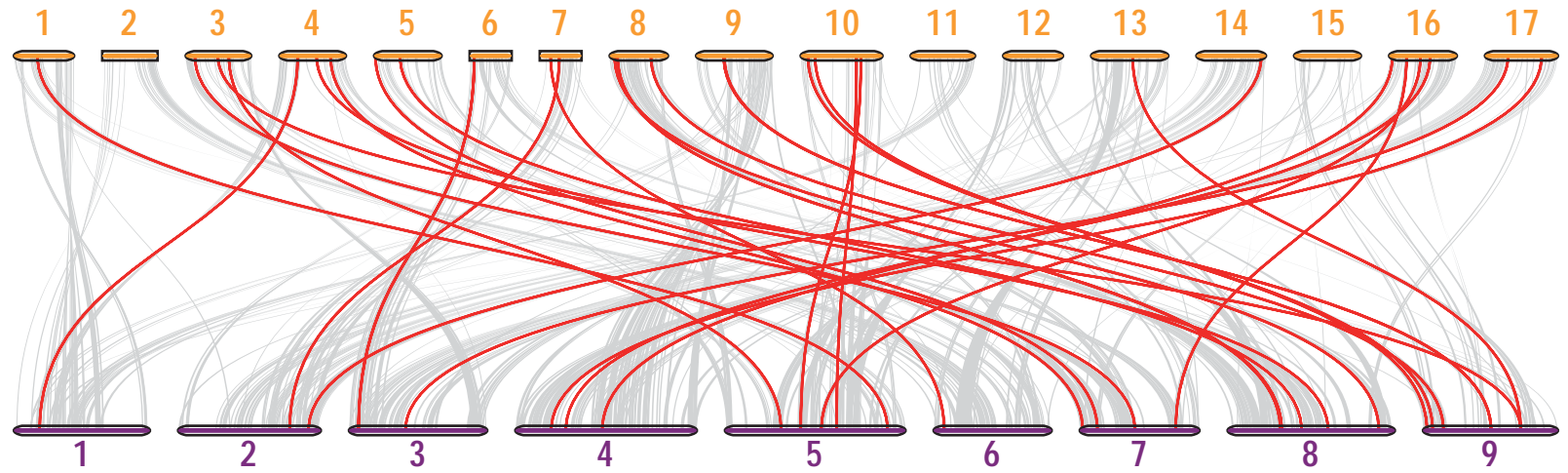

*L. sativa*

Supplement: Supplementary file 1 [file plants-08-00393-s001.zip › Figure S18/Figure S18.pdf]

*H.annus*

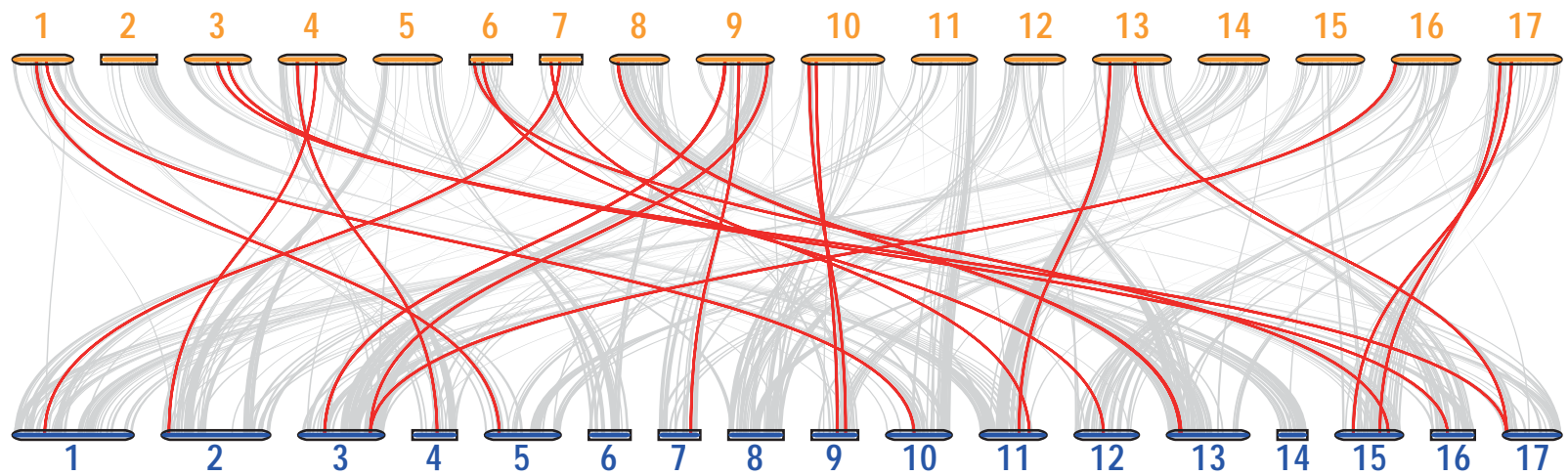

*C.cardunculus*

Supplement: Supplementary file 1 [file plants-08-00393-s001.zip › Figure S19/Figure S19.pdf]

Motif 1

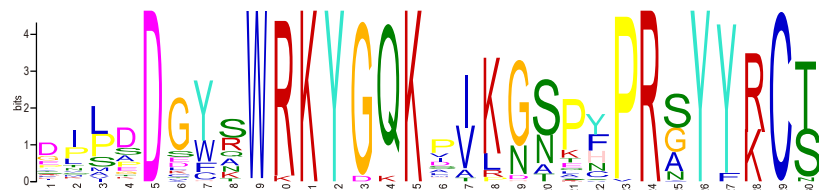

Motif 2

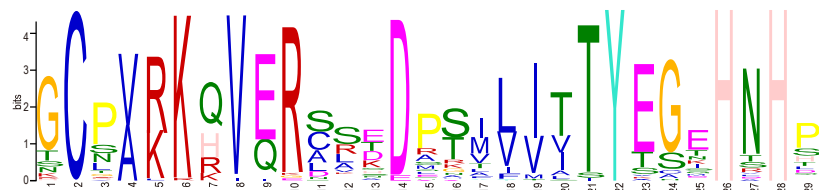

Motif 3

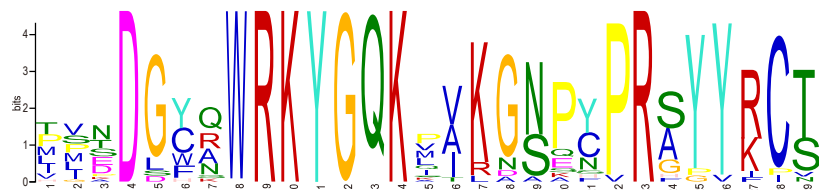

Motif 4

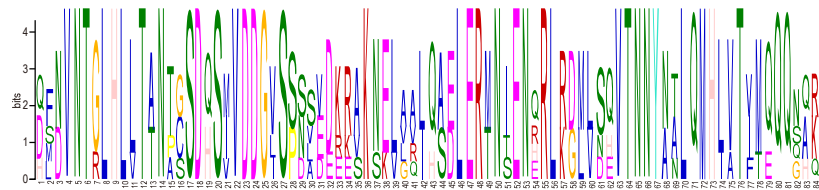

Motif 5

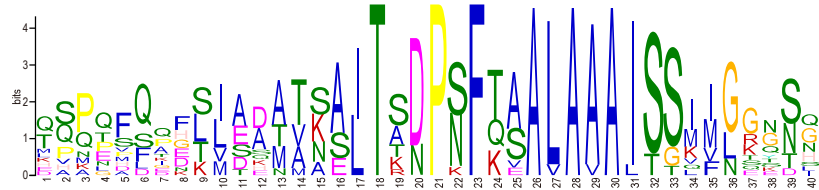

Motif 6

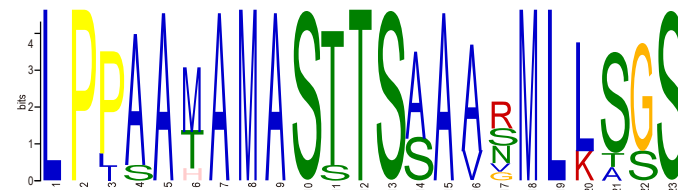

Motif 7

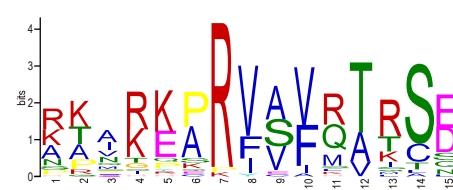

Motif 8

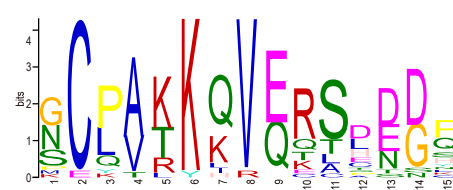

Motif 9

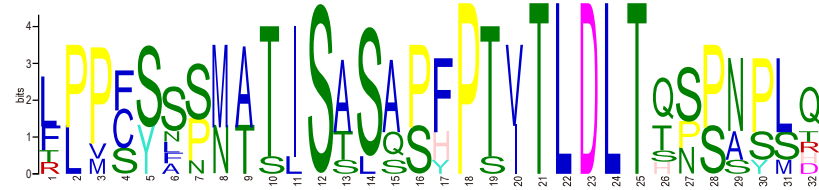

Motif 10

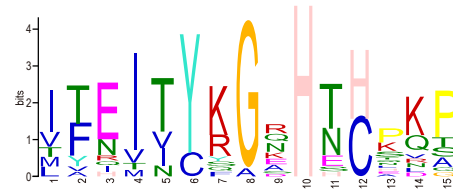

Supplement: Supplementary file 1 [file plants-08-00393-s001.zip › Figure S2/Figure S2a.pdf]

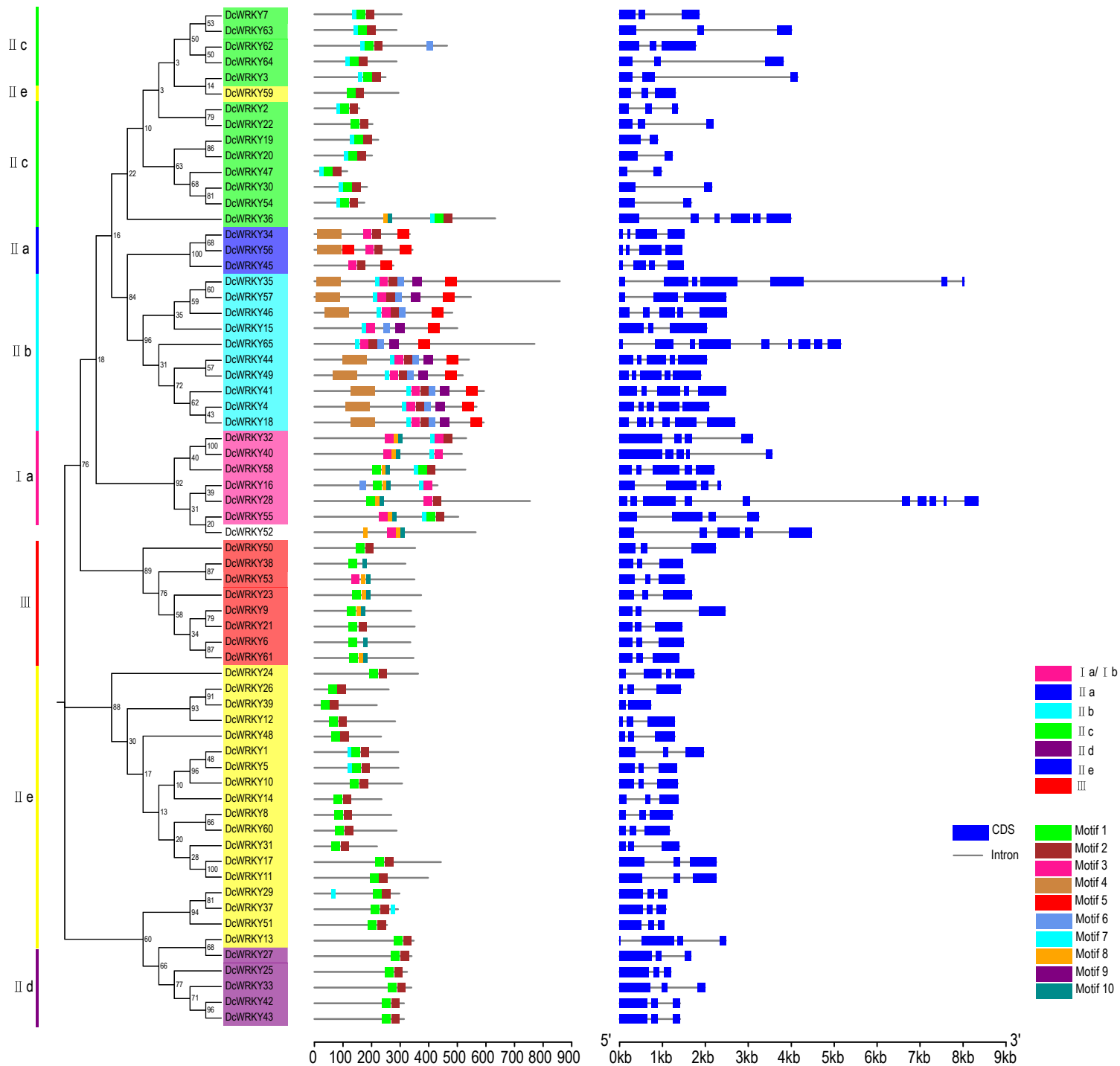

Supplement: Supplementary file 1 [file plants-08-00393-s001.zip › Figure S2/Figure S2b.pdf]

Motif 1

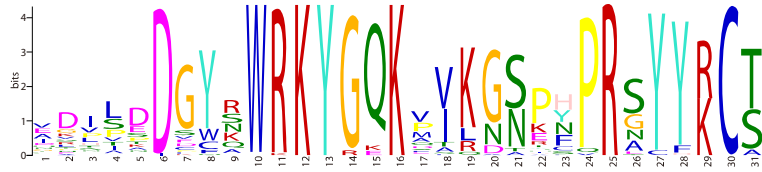

Motif 2

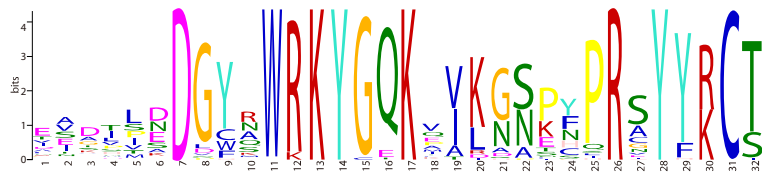

Motif 3

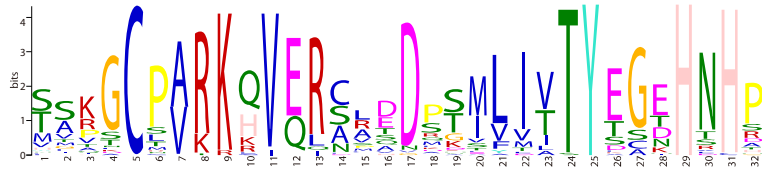

Motif 4

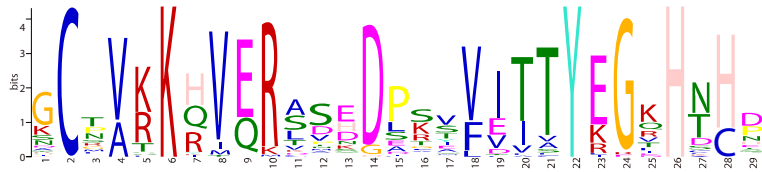

Motif 5

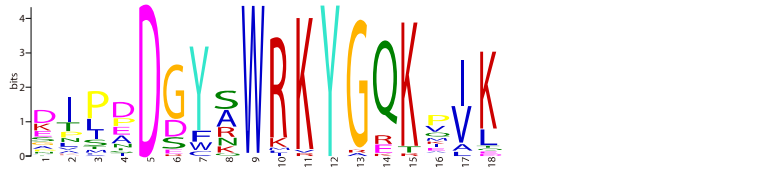

Motif 6

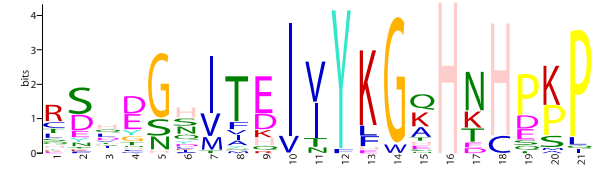

Motif 7

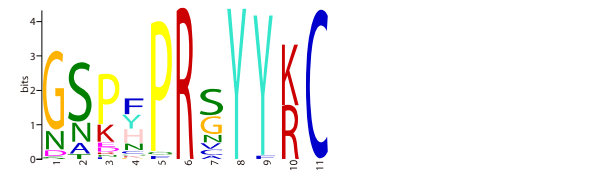

Motif 8

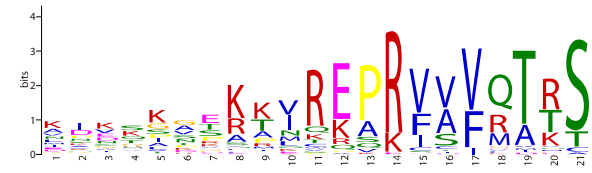

Motif 9

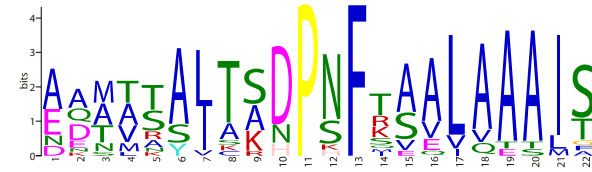

Motif 10

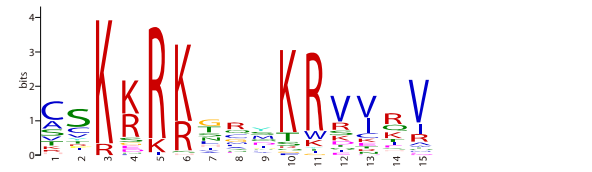

Supplement: Supplementary file 1 [file plants-08-00393-s001.zip › Figure S3/Figure S3a.pdf]

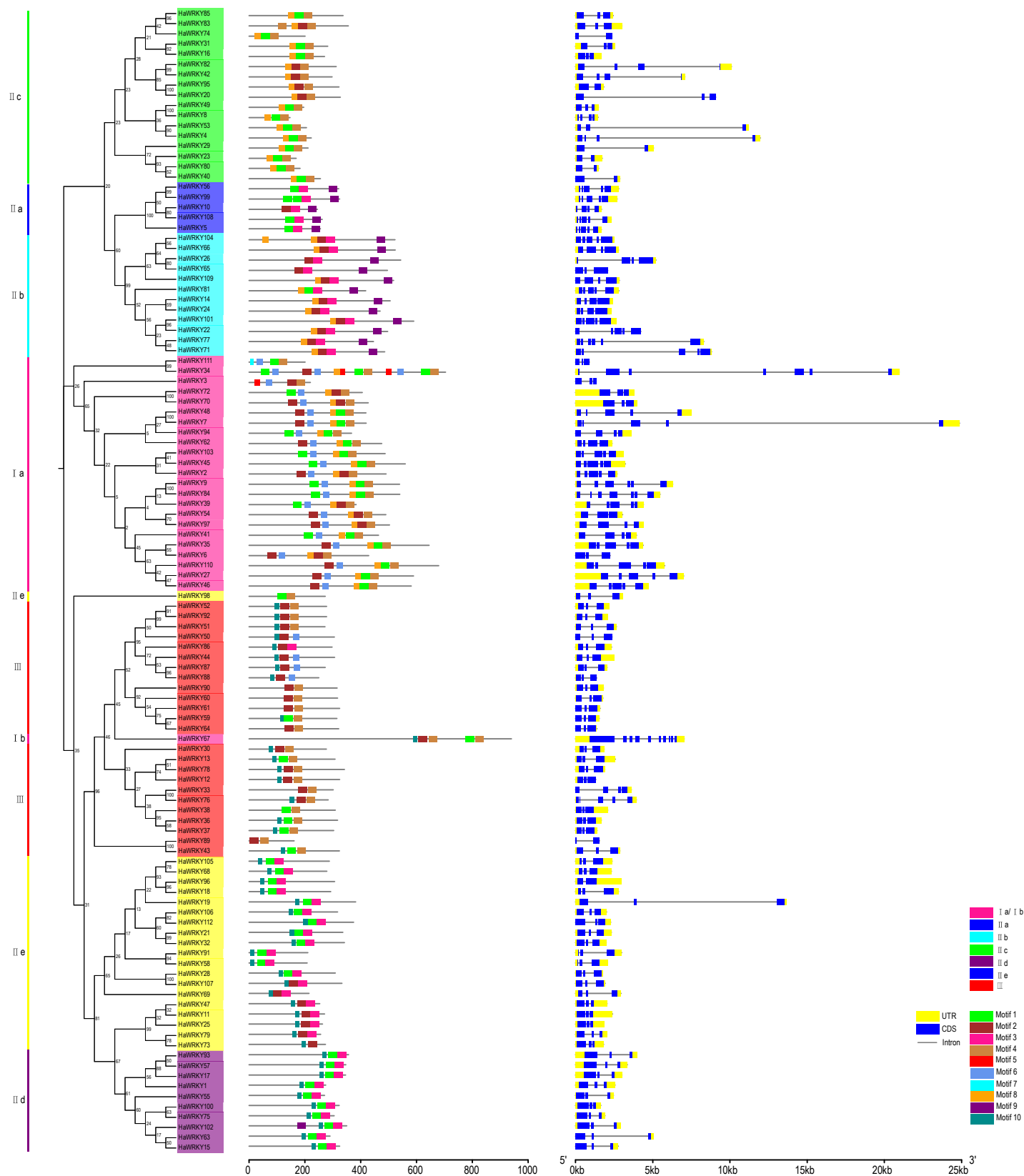

Supplement: Supplementary file 1 [file plants-08-00393-s001.zip › Figure S3/Figure S3b.pdf]

Motif 1

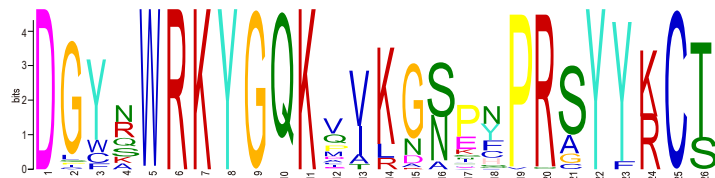

Motif 2

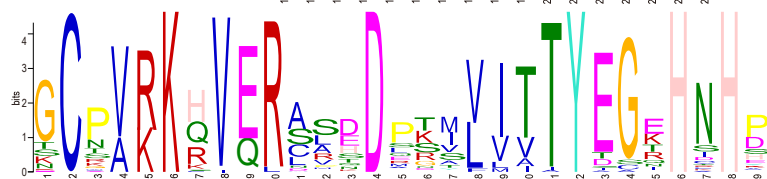

Motif 3

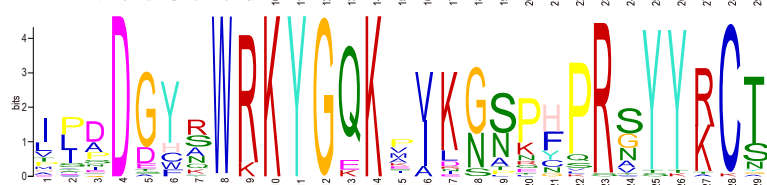

Motif 4

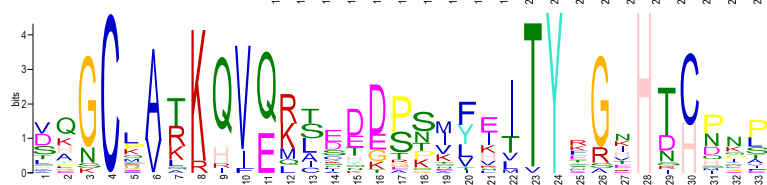

Motif 5

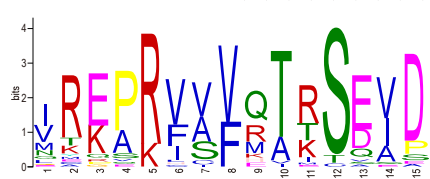

Motif 6

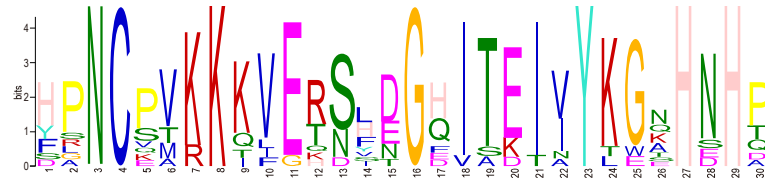

Motif 7

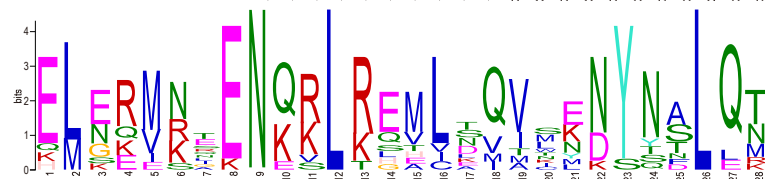

Motif 8

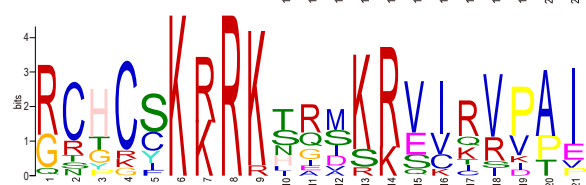

Motif 9

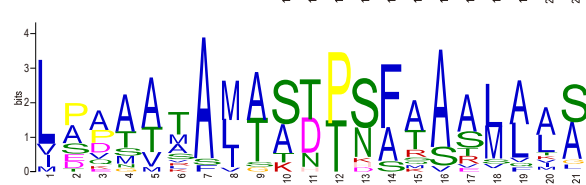

Motif 10

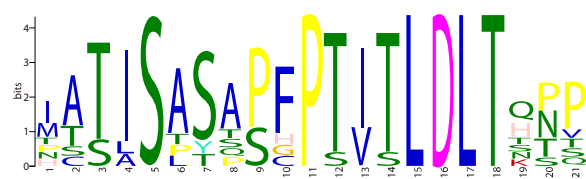

Supplement: Supplementary file 1 [file plants-08-00393-s001.zip › Figure S4/Figure S4a.pdf]

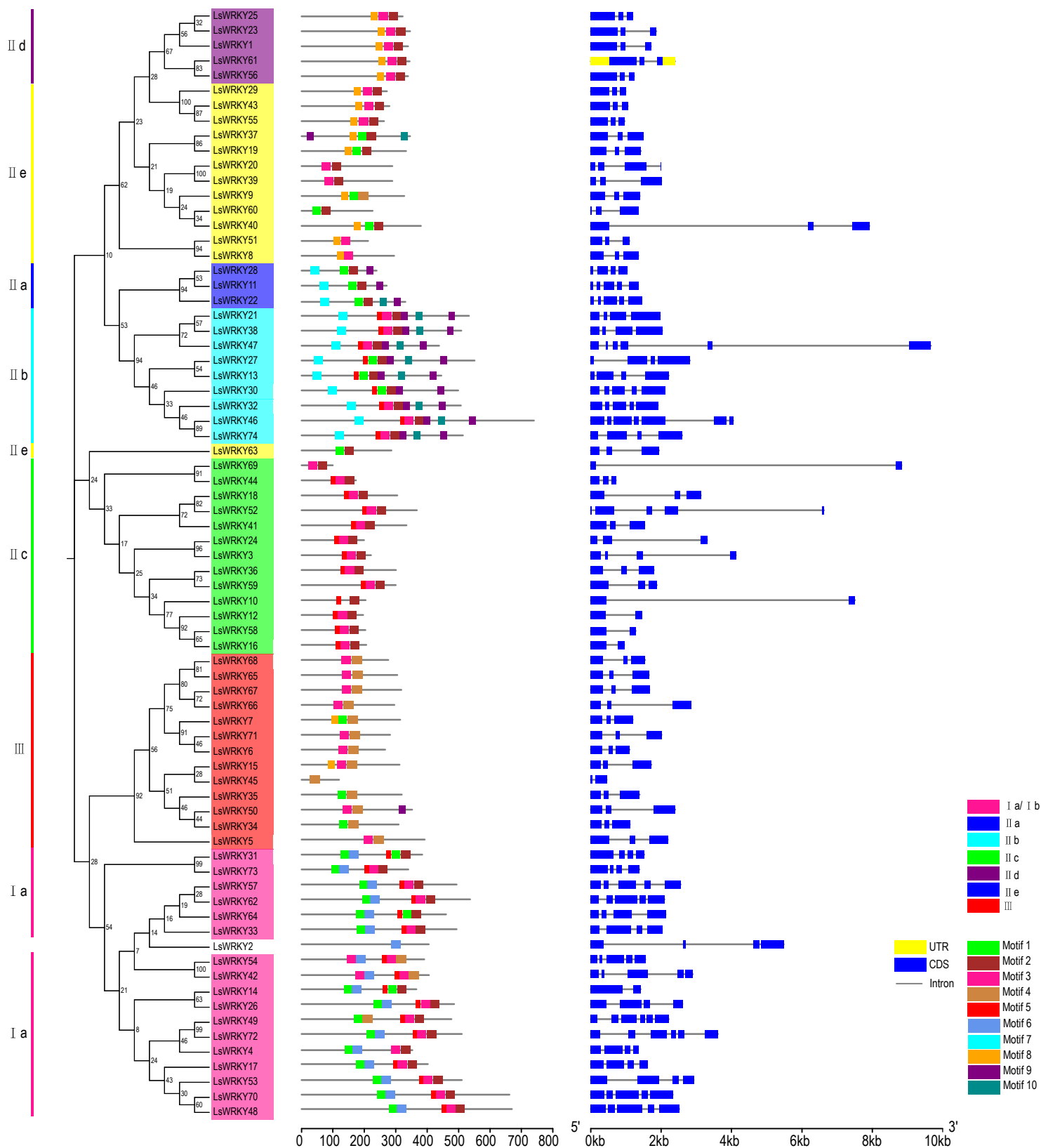

Supplement: Supplementary file 1 [file plants-08-00393-s001.zip › Figure S4/Figure S4b.pdf]

Motif 1

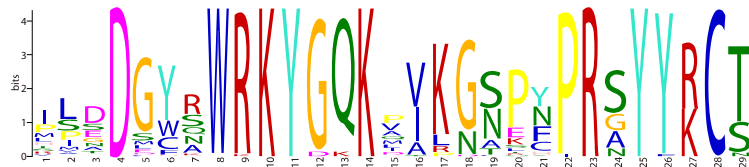

Motif 2

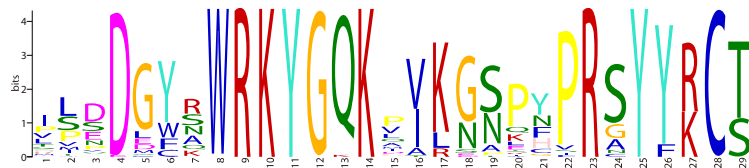

Motif 3

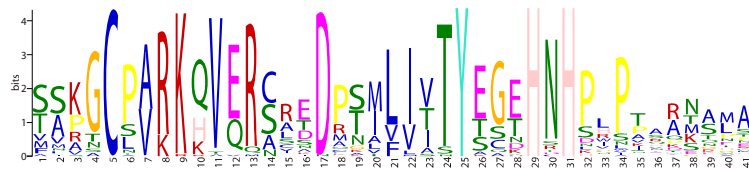

Motif 4

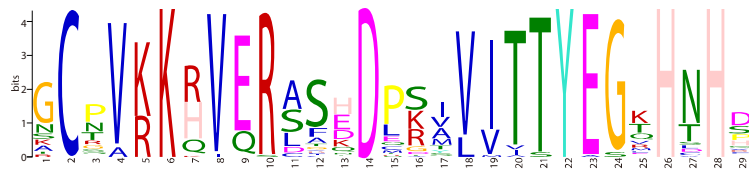

Motif 5

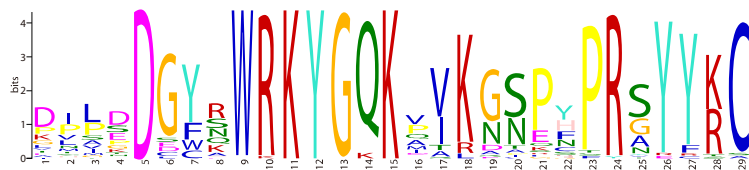

Motif 6

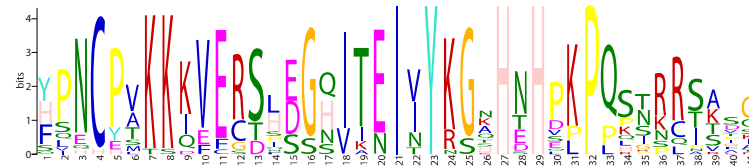

Motif 7

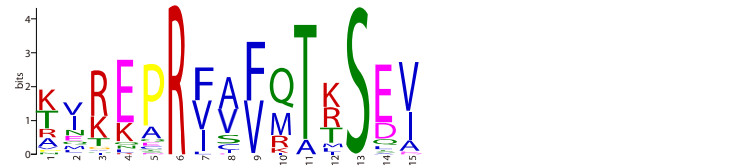

Motif 8

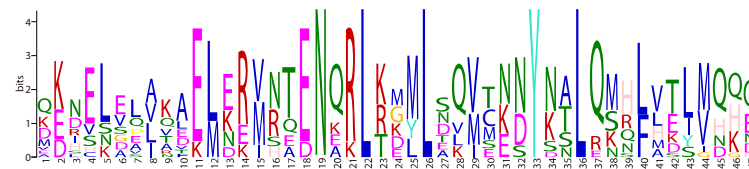

Motif 9

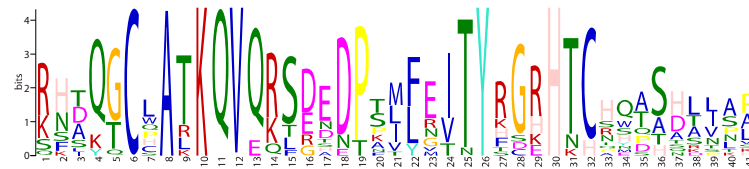

Motif 10

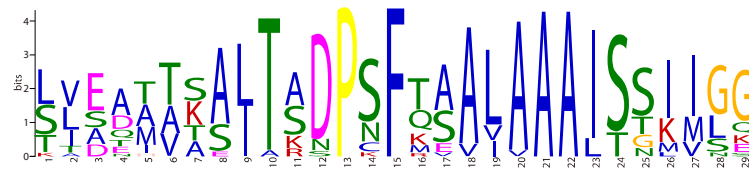

Supplement: Supplementary file 1 [file plants-08-00393-s001.zip › Figure S5/Figure S5a.pdf]

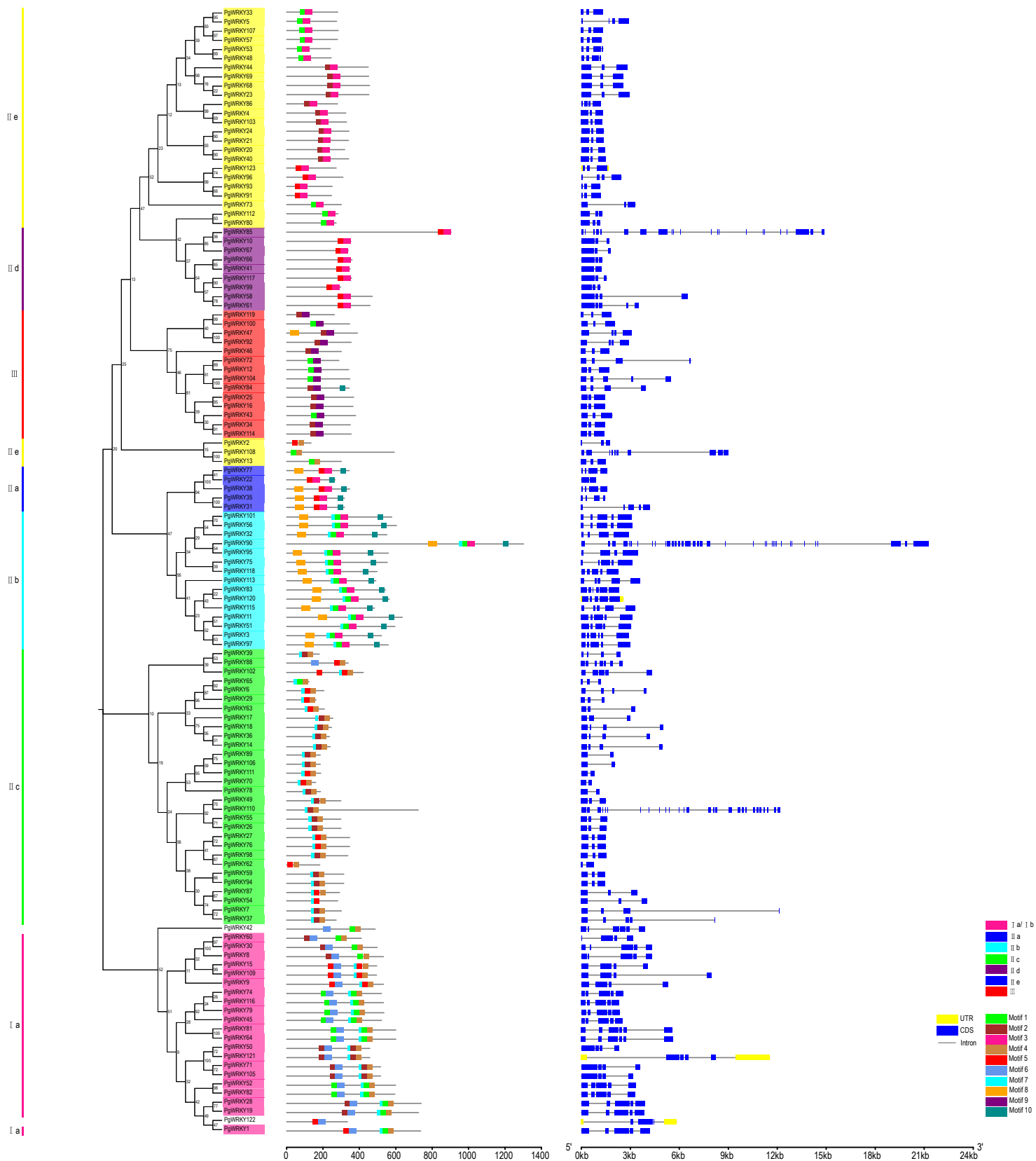

Supplement: Supplementary file 1 [file plants-08-00393-s001.zip › Figure S5/Figure S5b.pdf]

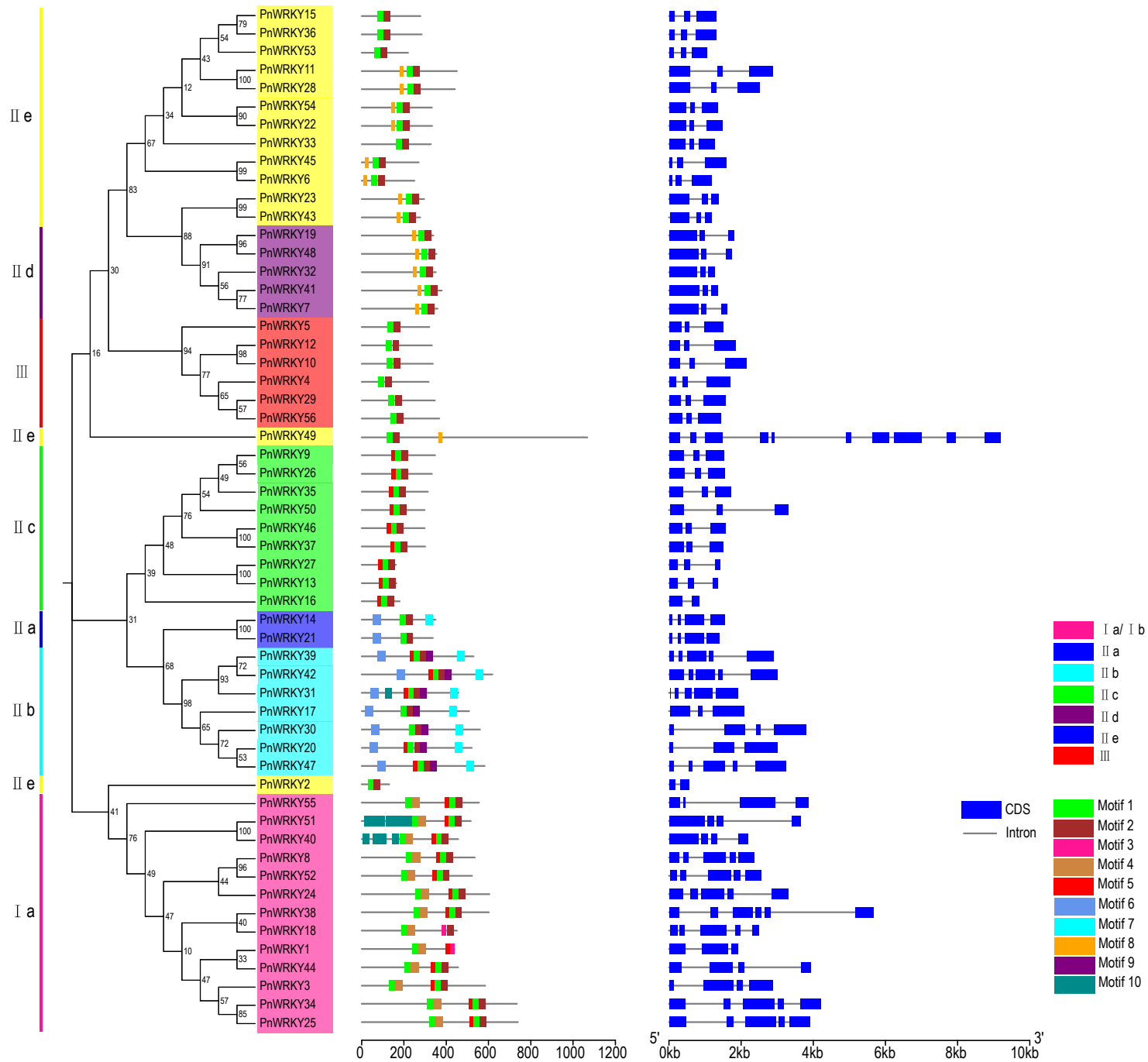

Supplement: Supplementary file 1 [file plants-08-00393-s001.zip › Figure S6/Figure S6b.pdf]

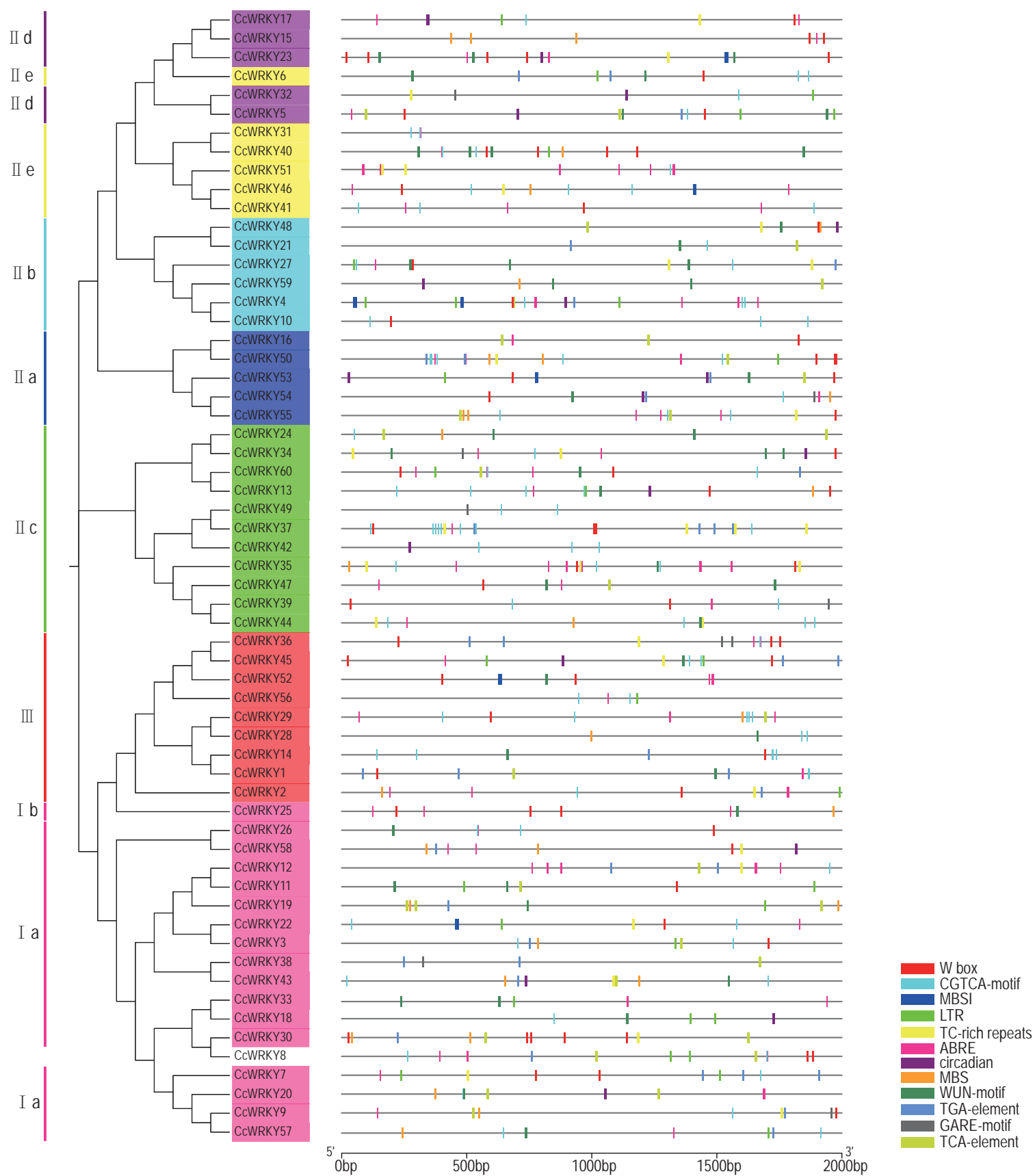

Supplement: Supplementary file 1 [file plants-08-00393-s001.zip › Figure S7/Figure S7.pdf]

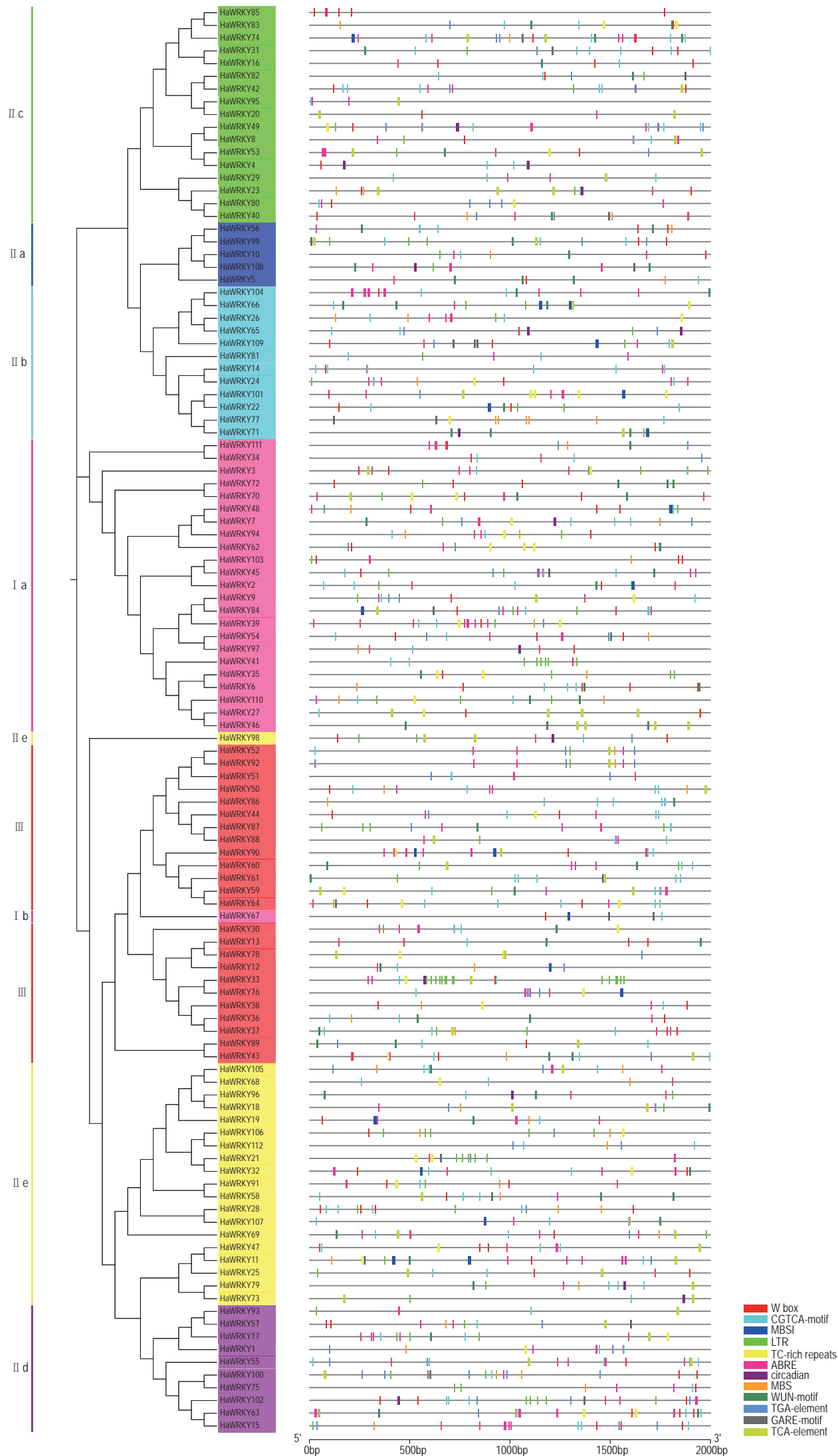

Supplement: Supplementary file 1 [file plants-08-00393-s001.zip › Figure S8/Figure S8.pdf]

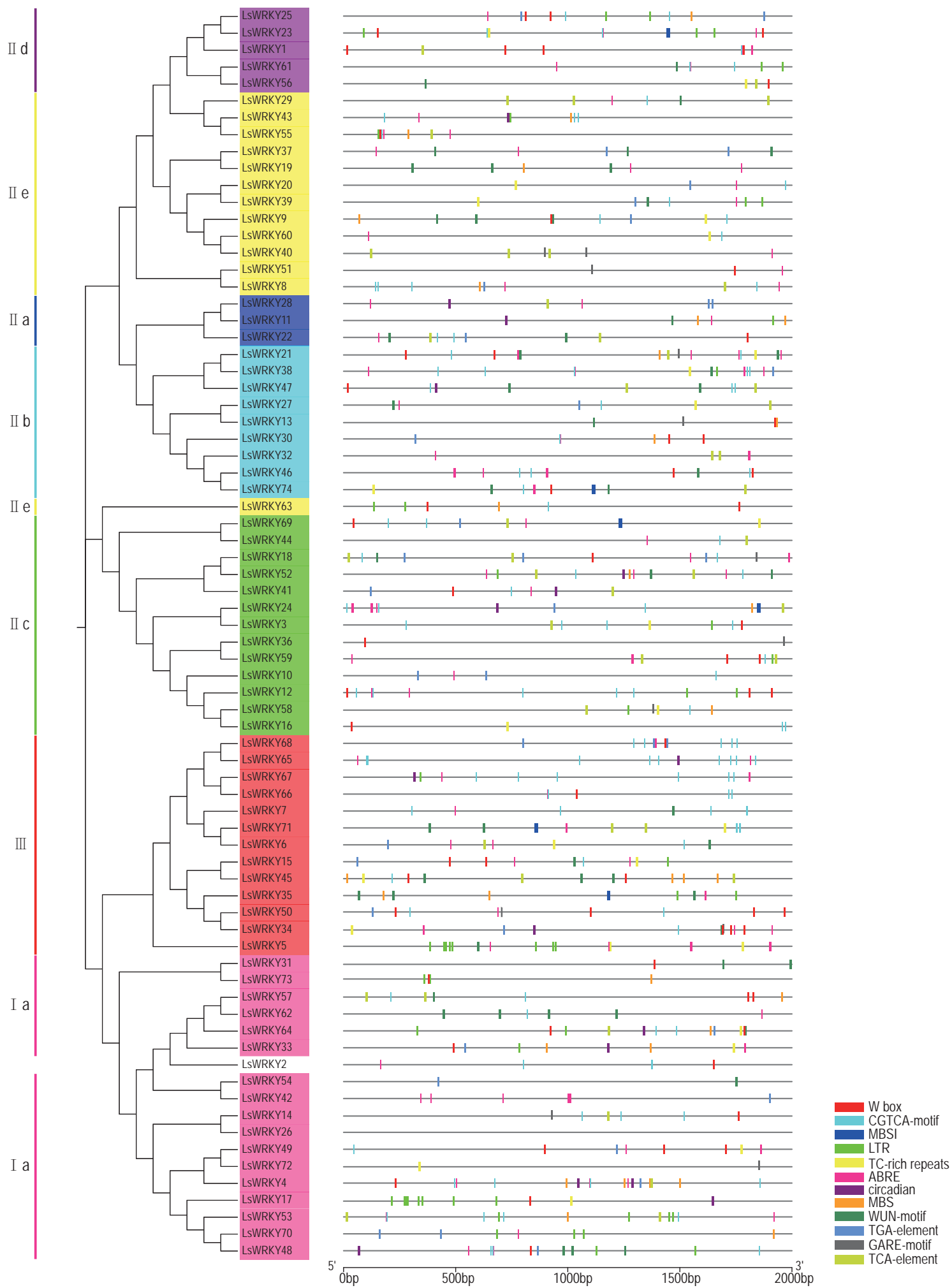

Supplement: Supplementary file 1 [file plants-08-00393-s001.zip › Figure S9/Figure S9.pdf]
