# Supplementary material for: Genome-Wide Identification of WRKY Transcription Factors in the Asteranae
Source: Plants (Basel). 2019 Oct 1;8(10):393. doi: 10.3390/plants8100393 (PMC6843914; doi:10.3390/plants8100393)
Supplement: Supplementary file 1 [file plants-08-00393-s001.zip › Figure S12/Figure S12.doc]

**Figure S12.** Distribution of *DcWRKY* genes on carrot chromosomes. Vertical bars represent chromosomes. The different groups of *DcWRKY* genes are color-coded with unclassifiable genes shown in black. Base-pair positions are indicated on the left.
