# Supplementary material for: Genome-Wide Identification of WRKY Transcription Factors in the Asteranae
Source: Plants (Basel). 2019 Oct 1;8(10):393. doi: 10.3390/plants8100393 (PMC6843914; doi:10.3390/plants8100393)
Supplement: Supplementary file 1 [file plants-08-00393-s001.zip › Figure S15/Figure S15.doc]

**Figure S15.** Synteny analysis of sunflower *HaWRKY* genes. Red lines indicate segmentally duplicated gene pairs, and gray lines connect syntenic blocks in the genome. Chromosomes 1–17 are represented by different colors.
