# Supplementary material for: Genome-Wide Identification of WRKY Transcription Factors in the Asteranae
Source: Plants (Basel). 2019 Oct 1;8(10):393. doi: 10.3390/plants8100393 (PMC6843914; doi:10.3390/plants8100393)
Supplement: Supplementary file 1 [file plants-08-00393-s001.zip › Figure S17/Figure S17.doc]

**Figure S17.** Synteny between genomes of carrot (*Daucus carota*) and sunflower (*Helianthus annuus*). Gray lines represent syntenic blocks between genomes, and red lines indicate syntenic *WRKY* gene pairs.
