# Supplementary material for: Genome-Wide Identification of WRKY Transcription Factors in the Asteranae
Source: Plants (Basel). 2019 Oct 1;8(10):393. doi: 10.3390/plants8100393 (PMC6843914; doi:10.3390/plants8100393)
Supplement: Supplementary file 1 [file plants-08-00393-s001.zip › Figure S4/Figure S4b.doc]

**Figure S4b.** Phylogenetic relationships, conserved motifs, and intron–exon structures of *LsWRKY* genes in lettuce. On the left is a phylogenetic tree of LsWRKY domains constructed by neighbor joining with 3,000 bootstrap replicates. LsWRKY conserved motifs are shown to the right of the tree. The 10 different conserved motifs are represented by different colors. *LsWRKY* gene intron–exon structures are shown on the far right.
