# Supplementary material for: Genome-Wide Identification of WRKY Transcription Factors in the Asteranae
Source: Plants (Basel). 2019 Oct 1;8(10):393. doi: 10.3390/plants8100393 (PMC6843914; doi:10.3390/plants8100393)
Supplement: Supplementary file 1 [file plants-08-00393-s001.zip › Figure S5/Figure S5b.doc]

**Figure S5b.** Phylogenetic relationships, conserved motifs, and intron–exon structures of *PgWRKY* genes in ginseng. On the left is a phylogenetic tree of PgWRKY domains constructed by neighbor joining with 3,000 bootstrap replicates. PgWRKY conserved motifs are shown to the right of the tree. The 10 different conserved motifs are represented by different colors. *PgWRKY* gene intron–exon structures are shown on the far right.
