# Supplementary material for: Genome-Wide Identification of WRKY Transcription Factors in the Asteranae
Source: Plants (Basel). 2019 Oct 1;8(10):393. doi: 10.3390/plants8100393 (PMC6843914; doi:10.3390/plants8100393)
Supplement: Supplementary file 1 [file plants-08-00393-s001.zip › Figure S8/Figure S8.doc]

**Figure S8.** *Cis*-acting elements in sunflower *HaWRKY* promoters. The distribution of 12 *cis*-acting elements in the 2,000bp upstream promoter are shown. The different types of *cis*-acting elements are represented by different colors as shown on the right. W box, WRKY binding site; CGTCA-motif, *cis*-acting regulatory element involved in the MeJA-responsiveness; MBS I, MYB binding site involved in flavonoid biosynthetic genes regulation; LTR, *cis*-acting element involved in low-temperature responsiveness; TC-rich repeats, *cis*-acting element involved in defense and stress responsiveness; ABRE, *cis*-acting element involved in the abscisic acid responsiveness; circadian, *cis*-acting regulatory element involved in circadian control; MBS, MYB binding site involved in drought-inducibility; WUN-motif, wound-responsive element; TGA-element, auxin-responsive element; GARE-motif, gibberellin-responsive element; TCA-element, *cis*-acting element involved in salicylic acid responsiveness
